# The current status of neglected tropical diseases in Japan: A scoping review

**DOI:** 10.1371/journal.pntd.0011854

**Published:** 2024-01-02

**Authors:** Yuriko Harada, Hanako Iwashita, Taeko Moriyasu, Sachiyo Nagi, Nobuo Saito, Mariko Sugawara-Mikami, Kota Yoshioka, Rie Yotsu, on behalf of Japan NTD Study Group

**Affiliations:** 1 Department of Hygiene and Public Health, Tokyo Women’s Medical University, Tokyo, Japan; 2 Office for Global Relations, Nagasaki University, Nagasaki, Japan; 3 Department of Parasitology, Institute of Tropical Medicine (NEKKEN), Nagasaki University, Nagasaki, Japan; 4 Department of Microbiology, Faculty of Medicine, Oita University, Oita, Japan; 5 West Yokohama Sugawara Dermatology Clinic, Kanagawa, Japan; 6 Department of Clinical Laboratory Science, Faculty of Medical Technology, Teikyo University, Tokyo, Japan; 7 School of Tropical Medicine and Global Health, Nagasaki University, Nagasaki, Japan; 8 Interfaculty Initiative in Planetary Health, Nagasaki University, Nagasaki, Japan; 9 Department of Tropical Medicine and Infectious Disease, Tulane School of Public Health and Tropical Medicine, New Orleans, Louisiana, United States of America; 10 Department of Dermatology, National Center for Global Health and Medicine, Tokyo, Japan; University of Heidelberg, GERMANY

## Abstract

Little attention has been paid to neglected tropical diseases (NTDs) in high-income countries and no literature provides an overview of NTDs in Japan. This scoping review aims to synthesize the latest evidence and information to understand epidemiology of and public health response to NTDs in Japan. Using three academic databases, we retrieved articles that mentioned NTDs in Japan, written in English or Japanese, and published between 2010 and 2020. Websites of key public health institutions and medical societies were also explored. From these sources of information, we extracted data that were relevant to answering our research questions. Our findings revealed the transmission of alveolar echinococcosis, Buruli ulcer, Chagas disease, dengue, foodborne trematodiases, mycetoma, scabies, and soil-transmitted helminthiasis as well as occurrence of snakebites within Japan. Other NTDs, such as chikungunya, cystic echinococcosis, cysticercosis, leishmaniasis, leprosy, lymphatic filariasis, rabies, and schistosomiasis, have been imported into the country. Government agencies tend to organize surveillance and control programs only for the NTDs targeted by the Infectious Disease Control Law, namely, echinococcosis, rabies, dengue, and chikungunya. At least one laboratory offers diagnostic testing for each NTD except for dracunculiasis, human African trypanosomiasis, onchocerciasis, and yaws. No medicine is approved for treatment of Chagas disease and fascioliasis and only off-label use drugs are available for cysticercosis, opisthorchiasis, human African trypanosomiasis, onchocerciasis, schistosomiasis, and yaws. Based on these findings, we developed disease-specific recommendations. In addition, three policy issues are discussed, such as lack of legal frameworks to organize responses to some NTDs, overreliance on researchers to procure some NTD products, and unaffordability of unapproved NTD medicines. Japan should recognize the presence of NTDs within the country and need to address them as a national effort. The implications of our findings extend beyond Japan, emphasizing the need to study, recognize, and address NTDs even in high-income countries.

## Author summary

Neglected tropical diseases (NTDs) are an umbrella term for 20 diseases and disease groups recognized by the World Health Organization. Although NTDs are usually considered to be a public health problem in low- and middle-income countries, people living in high-income countries could also be affected. In this scoping review, we aimed to summarize the latest evidence and information to understand the epidemiology of and public health response to NTDs in Japan. Japan officially reported a yearly average of 340 NTD cases between 2014 and 2017; however, we found that it does not accurately reflect the current status of NTDs in the country. Our review suggests that 15 of 20 NTDs can be considered to be important public health concerns. While echinococcosis, chikungunya, dengue, and rabies are under control by law, many other NTDs are largely neglected by Japan’s health systems and solely addressed by a limited number of physicians and researchers. We recommend that the Government of Japan should assume responsibility for enhancing surveillance, expanding access to diagnostics and medicines, and ensuring affordability of unapproved NTD drugs.

## Introduction

The World Health Organization (WHO) recognizes 20 diseases and disease groups as neglected tropical diseases (NTDs), which mainly affect people living in poverty [1]. NTDs accounted for approximately one percent of the global disease burden in 2012 [2] and at least 1.74 billion people require interventions against NTDs worldwide [1]. The United Nations’ Sustainable Development Goals (SDGs) include ending the epidemics of NTDs by 2030 as one of the global priorities [3].

Although NTDs are usually regarded as a public health problem in low- and middle-income countries, people living in high-income countries could be affected as well [4]. Hotez (2013) developed a concept of “blue marble health,” meaning that there is a paradoxical burden of NTDs among the poor living in wealthy countries [5]. Some underlying factors, including human migration [6] and climate change [7], are driving changes in the global distribution of NTDs. Researchers have investigated NTDs in high-income countries, for example in Italy [8, 9] and the United States [10]. High-income countries are no longer free from NTDs; Japan is no exception.

Little is known about the current status of NTDs in Japan. According to the Japan SDGs Action Platform run by the Ministry of Foreign Affairs of Japan, a yearly average of 340 NTD cases was reported between 2014 and 2017, which included cases of Buruli ulcer, cysticercosis, dengue, echinococcosis, foodborne trematodiases, leprosy, scabies, snakebite envenoming, and soil-transmitted helminthiases [11]. However, this is not a comprehensive case number because not all NTDs are reportable in Japan. To our knowledge, no literature has provided an overall situation of NTDs in Japan to date.

This scoping review aims to synthesize the latest evidence and information to understand epidemiology of and public health response to NTDs in Japan. To achieve this aim, the following guiding questions were addressed for each NTD: 1) What is the prevalence or incidence?; 2) How many cases are reported to the government bodies?; 3) Is the disease transmitted within Japan?; 4) Is any imported human case to Japan reported?; 5) Is there any human case surveillance implemented by the government bodies?; 6) Is there any control program implemented by government bodies?; 7) Is there any laboratory offering testing services for diagnosis?; and 8) Is there any vaccine or medicine available? Based on the findings, we also intend to develop recommendations to advance policy and research agenda for NTDs in Japan.

## Methods

**Fig 1:**
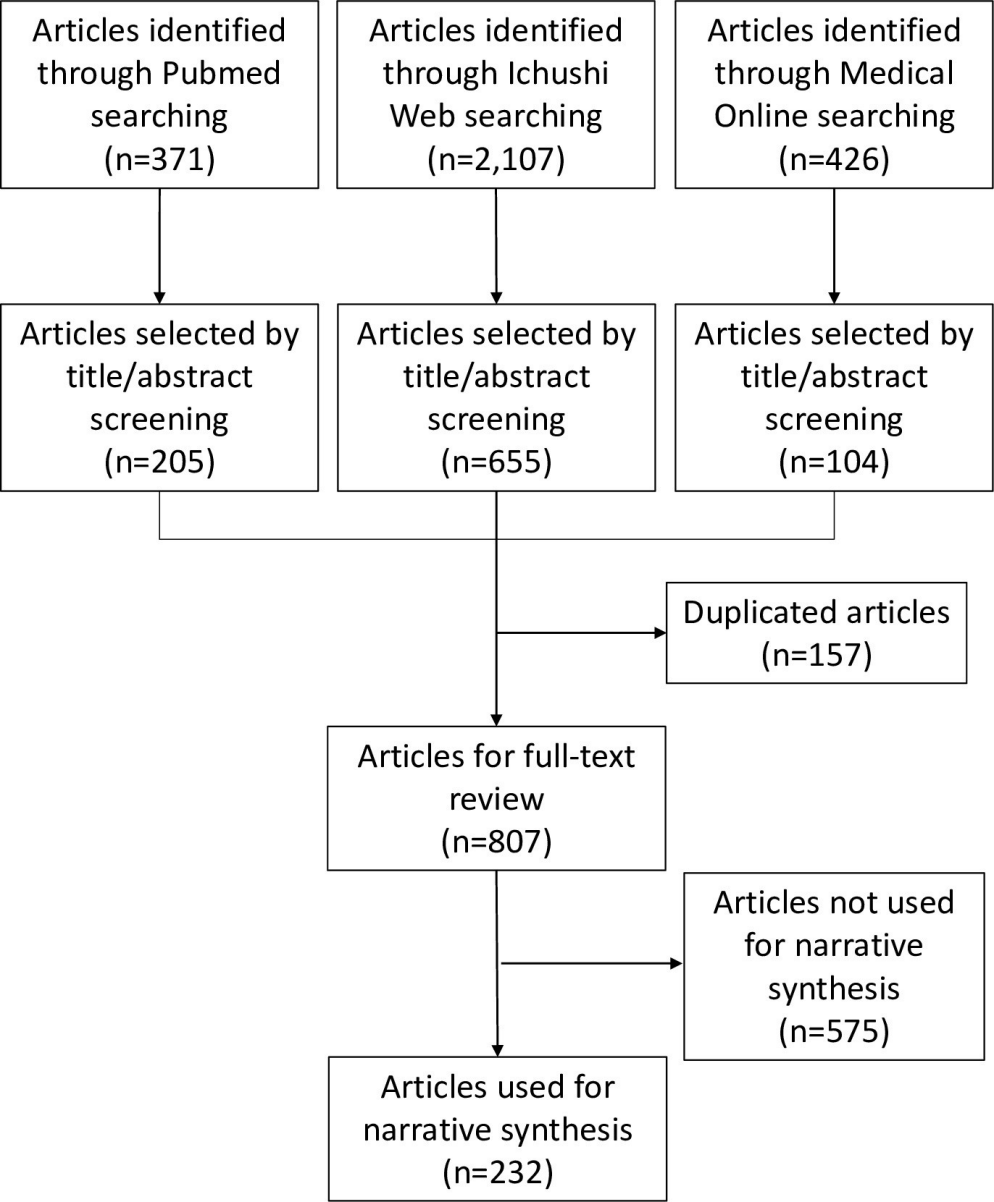
PRISMA ScR Flowchart.

Our paper takes the form of scoping review. A scoping review is a useful research tool when the purpose of the review is to identify the types of available evidence or to scope a body of literature in a given field [12]. Our aim of synthesizing knowledge and information regarding NTDs in Japan justifies our choice of performing a scoping review. In reporting this study, we followed the recommendations of PRISMA Extension for Scoping Reviews (PRISMA ScR) [13]. We described the PRISMA ScR Flowchart in Fig 1.

We developed a draft search protocol together including all authors of the Japan NTD Study Group. Two researchers independently tested the protocol by applying it to leishmaniasis and the other two researchers to scabies. We had a group discussion to compare the search results and revised the protocol until we reached a consensus. Once the protocol was established, one author was assigned to each NTD to collect data from the literature and websites. The same researcher synthesized the search results into a narrative. The narrative of each NTD was finally reviewed and revised by all authors. The following paragraphs describe our protocol in detail.

For our literature search, we used three online databases, namely, PubMed, Ichushi-Web, and Medical*Online. Ichushi-Web and Medical*Online are commonly used to search medical articles written in Japanese. Using the search strategy shown in S1 Document, we retrieved publications that met all of our inclusion criteria: 1) original research articles, clinical case reports, reviews, expert opinions, and commentaries that provide information about NTDs in Japan, 2) published in English or Japanese, and 3) published between January 1st, 2010 and December 31th, 2020. Then, we manually scanned the titles and abstracts of the retrieved articles and excluded articles that met any of our exclusion criteria: 1) an article focusing on the biology of pathogens, 2) an article focusing on product development, and 3) an article focusing on NTDs in countries other than Japan. Zotero (Corporation for Digital Scholarship, VA, USA) was used to store selected articles. As a data charting process, each researcher assessed the relevance of evidence in terms of our eight guiding questions. If the source of evidence included any information relevant to answering the guiding questions, we extracted that data for synthesis.

For the website search, we first browsed the Japanese government’s portal site [14] to identify laws framing responses to any NTDs, such as the Act on the Prevention of Infectious Diseases and Medical Care for Patients with Infectious Diseases (hereafter, the Infectious Diseases Control Law). When a disease is designated by the Infectious Diseases Control Law, that disease is subject to the national surveillance system. Second, we explored the Infectious Diseases Weekly Report [15], issued by the National Institute of Infectious Diseases (NIID) to identify notifications of NTDs between 2010 to 2020. Lastly, we collected information about the availability of vaccines, diagnostic testing, and medicines for NTDs in Japan. For vaccines, we searched the database of the Pharmaceuticals and Medical Devices Agency (PMDA) [16], a regulatory agency in Japan, to identify vaccine products approved in Japan. For diagnosis, the availability of diagnostic testing of any NTDs was searched based on the NIID’s Manual for the Detection of Pathogen [17] and an on-line directory named “Infectious Diseases Map for Advanced Progressive Laboratory Tests (IDMA)” [18], which is co-provided by the Japanese Association for Infectious Diseases and the Japanese Society for Clinical Microbiology. For medicines, we identified generic names of NTD medicines recommended by WHO using its Model List of Essential Medicine [19] or Fact Sheets [20], and then, checked whether that medicine has been approved by PMDA. We also looked into the website of the Research Group of Tropical Diseases and Parasitic Diseases [21] as this research group supplies some unapproved medicines for treatment of tropical diseases. In writing the Result section, we omitted citations to these websites to avoid redundancy.

For synthesis, each researcher used Table 1 to check whether she or he could collect data to answer the guiding questions. Then, the researcher summarized the data by constructing a narrative about epidemiology of and public health response to each NTD. All researchers read every narrative and revised it until we reached a consensus. The final narratives are presented in the Result section.

## Results

As Fig 1 shows, a total of 807 articles across 20 NTDs met our inclusion and exclusion criteria (see the numbers of selected articles for each disease in S1 Document). A full list of these 807 articles can be found in S2 Document. Among them, we used 232 articles for the narrative synthesis. In this Result section, we provided key findings from our review by disease area, following our guiding questions. Key findings are summarized in Table 1. A map of Japan showing the locations of prefectures mentioned in this review can be found in S3 Document.

**Table 1:** Summary of epidemiology of and response to neglected tropical diseases in Japan between 2010 and 2020.

NTD (WHO category)	NTD (sub-category)	Epidemiology				Response			
		Estimation of incidence or prevalence	Number of cases reported to NIID (2010–2020) [15]	Any evidence of domestic transmission?	Any evidence of imported cases?	Any surveillance of human cases by government bodies? [14]	Any control program by government bodies? [14]	Any laboratory offering confirmatory testing for diagnosis? [17, 18]	At least one medicine approved by PMDA for treatment? [16]
Buruli ulcer		n/a	52^a^	Yes [22, 23]	No	Yes	No	Yes	Yes (rifampicin, clarithromycin^b^, and levofloxacin^b^)
Chagas disease		3,000–4,500 prevalent cases [24]	-	Yes [24, 25]	Yes [26–31]	No	No	Yes	No
Cysticercosis		n/a	-	No	Yes	No	No	Yes	Yes (albendazole^b^ and praziquantel^b^)
Dengue/Chikungunya	Dengue	200–300 imported cases per year (More than 4,000 imported asymptomatic cases are expected.) [32]	2,755	Yes [33, 34]	Yes [35, 36]	Yes	Yes	Yes	-
	Chikungunya	n/a	142^c^	No	Yes [37, 38]	Yes	Yes	Yes	-
Dracunculiasis		n/a^d^	-	No	No	No	No	No	-
Echinococcosis	Alveolar echinococcosis	n/a	254	Yes [39–45]	Yes [46]	Yes	Yes	Yes	Yes (albendazole)
	Cystic echinococcosis	n/a	10	No	Yes [47–54]	Yes	Yes	Yes	Yes (albendazole)
Foodborne trematodiases	Clonorchiasis, Paragonimiasis	n/a	-	Yes [55–58]	Yes [56–60]	No	No	Yes	Yes (praziquantel)
	Fascioliasis	n/a	-	Yes [61]	Yes [62, 63]	No	No	Yes	No
	Opisthorchiasis	n/a^d^	-	No	No	No	No	Yes	Yes (praziquantel^b^)
Human African trypanosomiasis		n/a^d^	-	No	No	No	No	No	Yes (pentamidine^b^)
Leishmaniasis		n/a	-	No	Yes [64–70]	No	No	Yes	Yes (amphotericin B and paromomycin sulfate^b^)
Leprosy		Nearly zero new patients (among Japanese) [71]	41	No	Yes [71–75]	Yes	No	Yes	Yes (rifampicin, dapsone, and clofazimine)
Lymphatic filariasis		n/a	-	No	Yes [76, 77]	No	No	Yes	Yes (ivermectin^b^ and albendazole^b^)
Mycetoma	Mycetoma	n/a	-	Yes [78, 79]	No	No	No	Yes	Yes (itraconazlole^b^)
	Chromoblastomycosis	n/a	-	Yes [80–83]	Yes [81]	No	No	Yes	Yes (itraconazole)
	Sporotrichosis	n/a	-	Yes [84–87]	No	No	No	Yes	Yes (itraconazole, amphotericin B^b^, and potassium iodide)
	Paracoccidioidomycosis	n/a	-	No	Yes [88]	No	No	Yes	Yes (itraconazlole^b^)
Onchocerciasis		n/a^d^	-	No	No	No	No	No	Yes (ivermectin^b^)
Rabies		Zero incidence^e^ [89]	1	No	Yes [89]	Yes	Yes	Yes	-^f^
Scabies		80,000–150,000 incident cases per year [90]	-	Yes [91–98]	Yes [94]	No	No	Yes	Yes (ivermectin)
Schistosomiasis		n/a	-	No	Yes [99–107]	No	Yes	Yes	Yes (praziquantel ^b^)
Snakebites		1,000–3,000 incident cases per year [108–110]	-	Yes [111–127]	No	Yes^g^	No	-	Yes (Freeze-dried antivenoms^h^)
Soil-transmitted helminthiases	Ascariasis^i^	n/a	-	No^j^	No^j^	No	No	Yes	Yes (pyrantel and albendazole^b^)
	Trichuriasis	n/a	-	No	Yes [128]	No	No	Yes	Yes (mebendazole and albendazole^b^)
	Necatoriasis	n/a	-	No^j^	No^j^	No	No	Yes	Yes (pyrantel and albendazole^b^)
	Strongyloidiasis	At least 15 cases found per year [129]	-	Yes [130]	Yes [131]	No	No	Yes	Yes (ivermectin and albendazole^b^)
Trachoma		n/a^d^	-	No	No	No	No	Yes	Yes (azithromycin and tetracycline)
Yaws		n/a^d^	-	No	No	No	No	No	Yes (azithromycin^b^ and benzathine penicillin^b^)

Abbreviations

NIID: National Institute of Infectious Diseases

PMDA: Pharmaceuticals and Medical Devices Agency

Legend

-: Not applicable.

n/a: Data not available.

Notes

^a^ Data not available for 2018–2020.

^b^ For off-label use only.

^c^ Data not available for 2010.

^d^ The incidence and prevalence are probably zero since no evidence suggests either domestic transmission or importation.

^e^ Rabies is eliminated in Japan, but one imported case was exceptionally found in 2020.

^f^ Vaccines are approved by PMDA for pre- and post-exposure prophylaxis but rabies immune globulins are unapproved.

^g^ Surveillance of habu bites in Kagoshima and Okinawa only.

^h^ Yamakagashi antivenom is not approved by PMDA.

^i^ Not including *Ascaris suum* infections.

^j^ *Ascaris* and *Necator* infections are found in Japan [132, 133] but it is not clear where and when the transmission occurred.

### Buruli ulcer

#### Epidemiology

Buruli ulcer is an infectious disease caused by *Mycobacterium ulcerans*. The route of infection is still unknown [134] and human-to-human transmission has not been reported globally [135]. In Japan, the first case was found in 1979 in which a 19-year-old female with no travel history was diagnosed with the infection of *M. ulcerans* subsp. *shinshuense* (Mikoshiba et al. (1982) cited by [22]). Since then, a total of 75 cases have been reported to the Leprosy Research Center (LRC) at NIID by the end of 2020, with an average of one to three cases per year (Table 2). A concentration of cases has been observed in the central western region, especially in Okayama Prefecture. As with the first case, all reported cases have been ulcerated cases caused by *M. ulcerans* subsp. *shinshuense* [136, 137]. This subspecies differs from those identified in West Africa and Australia where the disease is also reported and is a domestic strain to Japan or possibly to some parts of Asia [135]. Cases diagnosed in Japan from 1980 to 2010 showed no evidence of patient contact with an aquatic environment [22] and the route of transmission remains unclear in Japan, as also the case in other parts of the world. Further investigation is needed to understand the route of transmission of *M. ulcerans* in Japan.

**Table 2:** Number of NTD cases reported to the National Institute of Infectious Diseases between 1999 and 2020.

Year	1999	2000	2001	2002	2003	2004	2005	2006	2007	2008	2009	2010	2011	2012	2013	2014	2015	2016	2017	2018	2019	2020
Buruli ulcer						1	1	1	3	2	5	9	10	4	10	7	4	2	6			
Chikungunya													10	10	14	16	17	14	5	4	49	3
Dengue	9	18	50	52	32	49	74	58	89	104	93	244	113	221	249	341	293	342	245	201	461	45
Echinococcosis, alveolar	6	20	13	8	20	25	18	19	23	23	26	19	22	15	19	28	27	27	29	18	27	23
Echinococcosis, cystic	1	2	2	2	1	1	2	1	2	1	1	0	3	2	2	0	0	0	1	1	1	0
Leprosy	19	14	13	16	8	12	6	7	12	7	2	4	5	3	4	5	7	3	2	3	5	4
Rabies	0	0	0	0	0	0	0	2	0	0	0	0	0	0	0	0	0	0	0	0	0	1

Source: National Institute of Infectious Diseases. We used the following webpages: https://www.niid.go.jp/niid/ja/ydata/10067-report-ja2019-20.html (for chikungunya, dengue, echinococcosis, and rabies), https://www.niid.go.jp/niid/ja/leprosy-m/1841-lrc/1707-expert.html (for leprosy), and https://www.niid.go.jp/niid/ja/bu-m/1842-lrc/1692-buruli.html (for Buruli ulcer). Exceptionally, data for the year 2020 were retrieved from NIID’s annual report (https://www.niid.go.jp/niid/images/idsc/idwr/IDWR2020/idwr2020-52-53.pdf). Since this 2020 report made no distinction between alveolar and cystic echinococcosis, we assumed that all cases were alveolar echinococcosis.

#### Public health response

No legal framework exists to respond to Buruli ulcer. Surveillance of Buruli ulcer is led by the LRC/ NIID, while there is no mandate issued by the government for the conduct. There could be under-reporting as the reporting of the disease is not mandatory, and the reporting only happens when attending physicians are aware of the reporting system. LRC compiles data and reports to WHO.

In Japan, the diagnosis of Buruli ulcer is based on the following criteria: 1) cutaneous manifestation compatible with Buruli ulcer, such as deep ulceration, necrotic tissue, presence of undermining, 2) a histopathological examination of the skin showing necrosis with limited inflammatory cells and/or presence of acid-fast bacilli, and 3) a positive result of a polymerase chain reaction (PCR) test detecting the *M. ulcerans*-specific IS2404 gene. PCR test is available at LRC as an administrative diagnostic test, and they receive samples nationwide. Mycobacterial culture is also recommended. However, it may take months to culture *M. ulcerans* and the results are often unavailable at the time of making treatment decisions; it is sometimes useful in examining the drug susceptibility.

Among two medicines recommended by WHO, rifampicin is approved in Japan for treatment of Buruli ulcer. The other medicine, clarithromycin, is also approved by PMDA but is available only for off-label use. In addition, levofloxacin is often added to the regimen, which is also available only for off-label use.

### Chagas disease

#### Epidemiology

Chagas disease is caused by a parasite, *Trypanosoma cruzi*. In Japan, Chagas disease is mainly found among immigrants from endemic countries, such as Brazil, Bolivia, and Peru. The number of Latino immigrants living in Japan has increased after Latin Americans of Japanese origin were allowed to work and stay in Japan in 1990 [138]. Nearly 230,000 Latin Americans nowadays reside in Japan [25], and it is estimated that 3,000–4,500 people are infected with *T. cruzi* [24].

*Triatoma rubrofasciata*, a potential vector of *T. cruzi*, is distributed among the Nansei Islands in Okinawa and Kagoshima Prefectures, but the risk of *T. cruzi* transmission by this vector species is considered low [139, 140]. The parasite can be spread via congenital transmission, blood transfusion, and organ transplantation [141–143], which are the potential transmission route for Chagas disease in Japan.

Several cases of *T. cruzi* infection have been reported among Latino immigrants living in Japan [26, 27], including two cases in which patients were treated with benznidazole [28–31], one case in which a pacemaker was implanted for Chagas cardiomyopathy [144], and two cases of death from Chagas disease [145, 146]. It is noteworthy that two cases of congenital transmission were also reported in Japan [24, 25] and both cases were treated with benznidazole [26, 31, 147, 148].

The Japanese Red Cross Society revealed that 3 out of 18,076 (0.017%) at-risk blood donors, defined as those who were born or raised in Latin America, those whose mothers were born or raised in Latin America, or those with a travel history to Latin America, were positive for *T. cruzi* antibody [149]. The first *T. cruzi*-infected blood donor was found in 2013 [138, 150]. Another *T. cruzi*-infected donor found in 2014 had previously provided blood products to 11 recipients but the transfusional transmission of *T. cruzi* has not been confirmed to date in Japan [25, 149, 151, 152]. No case of transmission by organ transplantation has been reported to date [24].

#### Public health response

There is no legal framework for responding to Chagas disease. The Japanese Red Cross Society currently runs a screening program targeting all at-risk blood donors [140]. In addition, a non-profit organization provides weekly screening at the Consulate-General of Brazil in Tokyo [25]. Laboratory testing for the diagnosis of Chagas disease is available at Saitama Medical University (https://ccidr-saitama.com/en/clinical-research-2/). None of the therapeutics, benznidazole nor nifurtimox, is approved by PMDA. Both therapeutics can be obtained from WHO, according to the Research Group of Tropical Diseases and Parasitic Diseases’ website.

### Dengue and chikungunya

#### Epidemiology

Dengue and chikungunya are febrile diseases caused by viruses that are transmitted to humans mainly by two mosquito species: *Aedes aegypti* and *Aedes albopictus. A. aegypti* has not established its life cycle in Japan, but there is a risk that this mosquito species can be imported into Japan [153]. *A. albopictus* is the main vector in Japan and its habitat has spread into the northern prefectures, probably due to global warming [154].

In Japan, more than 100 dengue cases have been reported to NIID each year since 2010, with the highest number of 461 in 2019 (Table 2). As only symptomatic cases are detected and reported to NIID, the actual number of infections, including asymptomatic cases, was estimated to be 20 times higher than the number of reported cases [32]. Dengue is largely an imported disease in Japan, as shown by one fatal case in 2016 with a person who returned from the Philippines [35]. From 1945 to 2013, no domestic transmission was confirmed [36], except for a case in which a nurse was infected by a needlestick injury in 1992 [155]. In 2013, a case of possible domestic transmission was reported, in which a German traveler was diagnosed with dengue fever after she visited Japan [156, 157]. In 2014, an outbreak of dengue fever occurred in Tokyo [33]. This outbreak resulted in the confirmation of 162 cases among visitors to public parks, such as Yoyogi Park in Tokyo [34]. Serotype 1 dengue virus was detected from mosquitoes collected in Yoyogi Park as well as from patients who had visited the park [158–161]. The virus was considered to have originated from Southeast Asia or China [162].

Between 2011 and 2020, a total of 142 chikungunya cases have been reported to NIID (Table 2). To date, all reported cases were imported from outside Japan, such as Sri Lanka, Malaysia, Angola, Indonesia, the Philippines, Tonga, the Commonwealth of Dominica, Colombia, and Cuba [37, 38]. Case reports described how chikungunya was imported to Japan, including two cases of Japanese siblings who visited the Cook Islands in 2015 [163], a case of a traveler from Angola to Japan in 2016 [164], a case of a Japanese returning traveler from India [165], a case of a Japanese woman who worked in the Commonwealth of Dominica in 2014 [166], and a case of a Japanese returning traveler from Jamaica [167]. The Narita Airport quarantine station also reported three cases among travelers from Southeast Asia in 2013 [168].

#### Public health response

Dengue and chikungunya are covered by the Infectious Disease Control Law and any confirmed cases should be immediately reported to the NIID. The Quarantine Law requires quarantine stations at ports and airports to offer medical examinations and blood tests for the detection of dengue and chikungunya to travelers who present symptoms and risks of having been bitten by mosquitoes. The Ministry of Health, Labor, and Welfare issued a guideline in 2015 for the prevention of mosquito-borne infections [169]. This guideline defines the roles of stakeholders, including the national and prefectural governments. The NIID also published a manual for local governments to conduct epidemiological surveys and mosquito control [169]. When a case of domestic dengue transmission was confirmed in Okinawa Prefecture in 2019, the prefectural government responded immediately following these guidelines and successfully prevented further transmission [170].

NIID has published laboratory procedure manuals for both diseases and laboratory tests are available at the NIID and Prefectural Institutes of Public Health [171, 172]. In addition, diagnosis of dengue is available at medical facilities listed by the Japanese Society of Travel and Health (http://jstah.umin.jp/03posttravel/index.htm). Dokkyo Medical University’s Saitama Medical Center also offers testing for chikungunya.

### Dracunculiasis

#### Epidemiology

Dracunculiasis, or Guinea-worm disease, is caused by the parasitic worm, *Dracunculus medinensis*. In recent years, human cases have been reported only from some African countries. Our review found no evidence of dracunculiasis in Japan.

#### Public health response

No legal framework covers dracunculiasis. Laboratory services to support diagnosis of dracunculiasis are not available in Japan.

### Echinococcosis

#### Epidemiology

Human echinococcosis involves two diseases: cystic echinococcosis caused by *Echinococcus granulosus* and alveolar echinococcosis caused by *Echinococcus multilocularis*. Between 2010 and 2019, a total of 10 and 231 cases were reported to NIID for the infection of *E. granulosus* and *E. multilocularis*, respectively. The reporting rates are relatively stable over the last 20 years for both infections (Table 2).

In Japan, cystic echinococcosis is considered to be an imported disease [47–49]. The infection of *E. granulosus* was confirmed in a Japanese female who might have been infected in the United Kingdom [50] and immigrants from Afghanistan [51], China [47], Nepal [52, 53], and Peru [54]. While *E. granulosus* seems to be continuously being introduced into Japan by imported cattle [173], there is no evidence that this parasite has established a transmission cycle infecting humans within Japan.

In contrast, *E. multilocularis* infection is endemic to the northern island of Hokkaido Prefecture. Between 1924–1926, foxes infected with *E. multilocularis* were artificially introduced to Hokkaido for rodent control by mankind [174]. Since the first human case was found in 1936, the infection has spread over Hokkaido by the 1980s [175]. The majority of human cases have occurred in Hokkaido, with some exceptions found in other prefectures [39]. Several clinical cases are well documented, including Japanese adults living in Hokkaido [40–42], a pediatric case in Hokkaido in which the patient died of liver failure [43], adult cases found in Chiba and Kanagawa Prefectures who had traveled to Hokkaido [44, 45], and a Bolivia-born female with a history of traveling to Hokkaido who was co-infected by *E. granulosus* and *E. multilocularis* [46]. In addition to foxes, the animal infection has also been reported in rats [176], horses [177, 178], pigs [39], dogs [179–181], a zoo-raised Diana monkey [182], and a flying squirrel [183]. The further spread of *E. multilocularis* from Hokkaido to other areas due to the travel of infected humans and animals is a significant concern in Japan. NIID considers that *E. multilocularis* has established its life cycle among wild dogs in some areas of Aichi Prefecture, but no human case has been yet reported [184].

#### Public health response

Echinococcosis is covered by the Infectious Diseases Control Law and any infected cases should be immediately reported to NIID. The Hokkaido local government has implemented an echinococcus control program, combining health education, screening for human echinococcosis, vector control, and improvement of clean water supply, which is considered to be key to preventing an outbreak of human infections in Hokkaido Prefecture [175]. NIID published a manual of the diagnostic procedure to detect and report echinococcus infections [185]. According to this manual, differential diagnosis is available at the NIID and the Hokkaido Institute of Public Health. For treatment, the WHO-recommended albendazole is approved by PMDA.

### Foodborne trematodiases

#### Epidemiology

Foodborne trematodiases are caused by intakes of fish, crabs, or vegetables contaminated by the larvae of trematode. WHO recognizes four foodborne trematodiases: clonorchiasis, opisthorchiasis, fascioliasis, and paragonimiasis. Of these, clonorchiasis, fascioliasis, and paragonimiasis are endemic in Japan. Imported cases of opisthorchiasis have been reported in the past, but we found no literature for this review.

Clonorchiasis is caused by *Clonorchis sinensis* parasitizing the bile ducts in the liver. In Japan, *C. sinensis* used to be widely distributed all over the country except in Hokkaido Prefecture. The number of cases has fallen drastically after the 1970s as the population of the intermediate host, *Parafossarulus* snails, declined; however, the domestic transmission is not completely contained [55]. Sporadic imported cases and a few domestic cases are reported to date in Japan [56, 57].

Fascioliasis is caused by *Fasciola hepatica* and *Fasciola gigantica*. *Fasciola* spp. is primarily found in the ungulates, such as cattle, sika deer, and wild boars. The prevalence of fascioliasis in beef cattle has gradually decreased from 18.6% in 1964 to 0.06% in 2010; however, *Fasciola* spp. is still detected in cattle produced all over Japan [186]. A few cases of human fascioliasis have been reported, and there are risks of domestic transmission among small-scale cattle ranchers and their neighbors. The consumption of vegetables contaminated with metacercaria could be a source of *Fasciola* spp. infections in Japan [61]. In addition, some imported cases of human fascioliasis from Vietnam and China have been reported [62, 63].

Paragonimiasis is caused by an infection with *Paragonimus* spp. in the human lungs. *P. westermani* and *P. miyazakii* are widely distributed in all regions of Japan except in Hokkaido Prefecture [187]. About 50 cases of paragonimiasis continue to be reported annually [188]. *Paragonimus* spp. is transmitted by the consumption of inadequately cooked freshwater crabs [187, 189] or meat of wild boar and sika deer [190–193]. Some basins in Chiba Prefecture are identified with a high risk of domestic transmission as more than 90% of freshwater crabs were found with *P. westermani* [194]. In contrast to the decreasing number of cases among Japanese nationals, the number of cases among migrants living in Japan is increasing [58–60].

#### Public health response

Under the Food Sanitation Act, foodborne trematodiases are reportable to health centers as food poisoning events, but the food poisoning statistics do not count them as separate diseases. Confirmatory diagnosis is made by morphological identification of eggs but in Japan, diagnosis is based on a combination of imaging with X-rays and MRI, parasite-specific antibody tests, and molecular confirmation. Serological tests of foodborne trematodiases are available at Miyazaki University and Aichi Medical University. The Department of Parasitology of NIID offers both serological and molecular tests. Praziquantel is approved by PMDA for treatment of clonorchiasis and paragonimiasis. It can also be used for treatment of opisthorchiasis as an off-label drug. Triclabendazole is recommended by WHO for treatment of fascioliasis and paragonimiasis but is not approved by PMDA. Triclabendazole is stored by the Research Group of Tropical Diseases and Parasitic Diseases for clinical trials.

### Human african trypanosomiasis

#### Epidemiology

Human African trypanosomiasis, or sleeping sickness, is caused by the parasite *Trypanosoma brucei*. Transmitted by tsetse flies, this disease is endemic in sub-Saharan African countries. Our review found no evidence of human African trypanosomiasis in Japan. Even if people infected with *T. brucei* come to Japan from the endemic regions, the risk of transmission is considered low because no tsetse flies have been found in the country [195].

#### Public health response

No legal framework is available for human African trypanosomiasis. The Japanese Red Cross Society does not take blood donations from those who have lived in Africa and have a history of the disease. No institution offers diagnostic testing. Among six WHO-recommended drugs, namely, pentamidine, suramin, melarsoprol, eflornithine, nifurtimox, and fexinidazole, only pentamidine is approved by PMDA for off-label use. Suramin, melarsoprol, and eflornithine can be obtained from WHO, according to the Research Group of Tropical Diseases and Parasitic Diseases.

### Leishmaniasis

#### Epidemiology

Leishmaniasis is caused by infection with *Leishmania* parasites, which are transmitted by sandfly species. In Japan, a small number of imported cases of cutaneous and mucocutaneous leishmaniasis have been documented. These cases have been found in three groups, including Japanese nationals who came back from overseas, such as Venezuela [64], Burkina Faso [65], and Brazil [66]; Japanese descendants who had lived overseas, such as in Bolivia [67] and Brazil [68]; and non-Japanese nationals who came to Japan, such as a student from Sri Lanka [69], a child from Syria [64], and an adult from Ethiopia [70]. In 2006 and 2007, two cases of canine leishmaniasis were found in dogs that used to live in Italy [196]. One of the vector species, *Phlebotomus squamirostris*, has been found in Japan [197]. Despite the continuous importation of the parasites and the presence of vectors within the country, there is no evidence of domestic transmission of *Leishmania* parasites to humans.

#### Public health response

No legal framework responds to leishmaniasis. Diagnosis and treatment of leishmaniasis have occurred occasionally, only when experienced physicians could suspect the infection, particularly seeing immigrants or returnees with skin lesions. Aichi Medical University Hospital offers parasitological tests for leishmaniasis. Among five medicines listed by the WHO, namely, amphotericin B, meglumine antimoniate, miltefosine, paromomycin, and sodium stibogluconate, only amphotericin B is approved by PMDA for treatment of leishmaniasis. Paromomycin sulfate is also approved by PMDA but is available for off-label use only. Miltefosine and sodium stibogluconate can be privately imported [21].

### Leprosy

#### Epidemiology

Leprosy, caused by *Mycobacterium leprae*, is nearly eliminated in Japan. Since 2008, the annual number of newly diagnosed cases has stayed below 10 (Table 2). While we found no evidence of recent domestic transmission, cases of leprosy continue to be reported. LRC reported seven new cases among Japanese nationals from 2010 to 2020 [71]. More than half of the new Japanese cases are reported from Okinawa Prefecture [198], where a few relapsed cases are also found every year [199]. Case reports describe conjugal infection among a couple [200], relapsed leprosy developing drug resistance [201], and leprous neuropathy [202]. Sequelae of leprosy are also documented, such as skin cancers formed at chronic ulcers [203, 204], intractable ulcers complicated by diabetes and the sequelae of leprosy [205], visual dysfunction [206, 207], and dysmasesis and dysphagia due to facial paralysis [208].

Alongside the domestic cases, there are also imported cases from endemic countries [72]. LRC reported a total of 38 new cases of foreign nationals from 2010 to 2020, which is 84% of the total number of new cases reported during the same period [71]. For example, three foreign nationals, including a Filipino, a Paraguayan, and a United State soldier born in Micronesia, were found with leprosy in Japan [73–75]. A Brazilian who had been treated for leprosy in Brazil 16 years ago was diagnosed with relapsed leprosy in Japan [209].

#### Public health response

Leprosy control in Japan has a history of over 100 years. The Leprosy Prevention Law was first established in 1907, then revised twice, and repealed in 1996 [210, 211]. The main approach of this law was to isolate patients in national leprosy sanatoria to break the chain of transmission, while the current scientific evidence tells us that this was not necessary, especially after the introduction of antibiotic treatment in the early 1950s. Between 1909 and 2010, a cumulative total of 56,575 patients were admitted to the 13 national sanatoria [212]. Although the law became a dead letter around 1970 [210], 1,718 persons still live in sanatoria as of 2016 [213]. The long-lasting isolation policy severely affected patients’ dignity and *raison d’etre* as human beings [214]. Even after their reintegration into society, ex-patients often face stigma and discrimination, and therefore many preferred to remain in sanatoria [215–217]. The patient associations continue to fight for the recovery of deprived human rights [213].

Currently, there is no legal framework for leprosy control, in terms of newly diagnosed cases. There are two existing laws aiming to restore the honor of those affected by the isolation policy and discrimination. LRC functions as a referral center for newly diagnosed cases and compiles data and reports to WHO. The LRC also provides administrative laboratory testing for the diagnosis of leprosy. Three WHO-recommended medicines, namely, rifampicin, dapsone, and clofazimine (multi-drug therapy; MDT), are approved by PMDA. The Japanese Leprosy Association publishes guidelines for diagnosis and treatment [218, 219]. Abolishment of the Leprosy Prevention Law in 1996 allowed any dermatologist to provide medical care to leprosy patients [220]; however, diagnosis of leprosy-related conditions tends to delay because of the lack of knowledge about leprosy among healthcare providers [72], no follow-up system for ex-patients, or patients’ hesitation to consult local physicians [204]. Current challenges include providing care to the small number of new, relapsed, or imported cases, as well as, addressing sequelae or complications among ex-patients [221].

### Lymphatic filariasis

#### Epidemiology

Lymphatic filariasis is a mosquito-borne disease caused by *Wuchereria bancrofti*, *Brugia malayi*, and *Brugia timori*. This disease was endemic in the Kyushu Region, Okinawa Archipelago, and Ogasawara Islands of Tokyo but was eliminated in the 1970s by large-scale community screening, awareness campaigns, chemotherapy of the infected people with diethylcarbamazine (DEC), and vector control [222]. This set of interventions was a new strategy at the time, which is now adapted by endemic countries worldwide. In recent years, we only experience, on a yearly average, one or two imported cases among migrants or travelers [76, 77].

#### Public health response

No legal framework responds to lymphatic filariasis. Serological and molecular testing is available at Aichi Medical University. One of the three WHO-recommended medicines, DEC, is approved by PMDA for treatment of lymphatic filariasis. The other two WHO-recommended medicines; ivermectin and albendazole, are approved by PMDA but are available for off-label use only.

### Mycetoma, chromoblastomycosis and other deep mycoses

In line with the WHO’s NTD road map [1], this section addresses four diseases: mycetoma, chromoblastomycosis, sporotrichosis, and paracoccidioidomycosis.

#### Epidemiology

Mycetoma is a chronic, granulomatous infectious disease and can be etiologically classified into two subgroups: eumycetoma caused by fungus and actinomycetoma caused by bacteria. About 20 pathogens are known to cause mycetoma [223]. Our review identified two cases of mycetoma which were caused by bacteria belonging to the *Nocardia* genra [78, 79]. *Nocardia brasiliensis* is the most common causative agent of cutaneous nocardiosis [79]. In Japan, mycetoma cases tend to be reported as bacterial or fungal infections rather than defining them as mycetoma because diagnosis of mycetoma largely relies on the identification of causative pathogens by molecular methods. Indeed, among 88 cases of *N. brasiliensis* infection found from 1995 to 2013, only 16 cases (18%) were reported as mycetoma [79]. This reporting practice seems to obscure the epidemiological understanding of mycetoma in Japan. Imported cases have not been reported for the study period.

Chromoblastomycosis is a fungal infectious disease often triggered by trauma. In Japan, *Fonsecaea monophora* is considered to be the most common causative agent [224]. Our review found case reports of *F. monophora* infection [80–82] as well as *F. nubica* infection [83]. While chromoblastomycosis is largely transmitted within Japan, an imported case from the Philippines was also found in 2008 [81]. Recently, 10 to 20 cases of chromoblastomycosis or phaeohyphomycosis have been reported every year [224].

Sporotrichosis is known to be associated with farming and gardening since its causative fungus can be found in soil or plants. Causative pathogens are morphologically similar fungi, including *Sporothrix schenckii*, *S. globosa*, and other *S*. spp. In Japan, a molecular study revealed that most of the clinical strains (291/300) previously identified as *S. schenckii* were actually *S. globosa* [225]. The guideline for treatment of fungus infections, published by the Japanese Dermatological Association, describes *S. globosa* as the main causative agent of sporotrichosis [224]. Our literature search identified 16 domestically transmitted cases [84–86]. Geographically, most of the cases were found in the river basins of the Kanto and Kyushu Regions [87]. This disease is commonly observed on the upper extremities in adults and on the face in children, although the number of pediatric cases is decreasing [224]. The number of newly diagnosed cases peaked in the 1980s and has gradually declined due to urbanization and a decrease in the number of farmers [226], with an average of approximately 10 cases per year since 2010 [224].

Paracoccidioidomycosis is caused by *Paracoccidioides brasiliensis*. The number of paracoccidioidomycosis cases increased rapidly in the 1990s, mainly among Brazilians coming to Japan, but has decreased since 2000 [227]. Between 1961 and 2015, 21 cases were reported and this disease is recognized as one of the imported fungal infections in Japan [88].

#### Public health response

No legal framework responds to mycetoma, chromoblastomycosis, sporotrichosis, and paracoccidioidomycosis. At the moment of this writing, IDMA provides a list of 17 laboratories. Among them, the Medical Mycology Research Center at Chiba University is known as a specialized diagnostic facility (http://www.pf.chiba-u.ac.jp/). Treatment of mycetoma depends on the causative pathogens. WHO does not specify any drug but itraconazole, which is described as treatment of eumycetoma in the WHO’s NTD road map [1]. Itraconazole is also included in the WHO Model Lists of Essential Medicines for treatment of chromoblastomycosis, sporotrichosis, and paracoccidioidomycosis. In Japan, itraconazole is approved by PMDA for treatment of chromoblastomycosis and sporotrichosis. In addition, amphotericin B and potassium iodide are recommended by the WHO Model Lists for treatment of sporotrichosis. Both medicines are approved by PMDA while amphotericin B is available for off-label use only. The Japanese Dermatological Association has a guideline for treatment of fungal infections published in 2019 [224].

### Onchocerciasis

#### Epidemiology

Onchocerciasis, also commonly known as river blindness, is caused by a parasite called *Onchocerca volvulus*. *O. volvulus* is transmitted by repeated bites from black flies (*Simulium* spp.). In Japan, no cases of onchocerciasis caused by *O. volvulus*, including imported cases, have been reported to date. In contrast, several human cases of zoonotic onchocerciasis, caused by *Onchocerca dewittei japonica*, have been reported in the last 20 years [228–232]. The terminal hosts of *O. dewittei japonica* are domestic animals, such as cattle and horses, as well as wild animals, such as wild boar and Japanese deer.

#### Public health response

No legal framework responds to onchocerciasis. No hospitals and laboratories offer diagnostic testing. Ivermectin, a medicine recommended by WHO for treatment of onchocerciasis, is approved by PMDA but for off-label use only.

### Rabies

#### Epidemiology

In Japan, there were large epidemics of rabies before the 1940s [89]. Rabies was successfully eliminated by 1957 and, as Table 2 shows, only a few cases have been reported in recent years: one imported case in 1970 and two cases in 2006 with travel histories abroad [233]. In 2020, one Filipino worker developed rabies in Japan, who was previously bitten by a dog back in the Philippines [234].

One study showed that the incidence of animal bites among Japanese expatriates and travelers in Thailand was 1.7 and 43.1 per 1,000 person-months, respectively [235]. Certain numbers of Japanese travelers are treated in Japan for possible rabies exposure abroad [236, 237]. The majority of exposures occurred in Asia due to dog or cat bites. One study showed that only 13.0% - 16.7% of travelers bitten by animals abroad received rabies immunoglobulin (RIG) before they returned to Japan [238].

#### Public health response

Three laws, the Rabies Prevention Law, the Domestic Animal Infectious Diseases Control Law, and the Infectious Diseases Control Law, frame prevention and control of human and animal rabies, such as registration and vaccination of domestic dogs, required quarantine of susceptible imported animals, and planning of science-based national actions. Notification of human rabies is mandatory. The government published a guideline for rabies control in 2001 and revised it in 2013 [239]. More about government rabies control is described elsewhere [240]. Laboratory diagnostic tests for rabies confirmation are available at NIID, Oita University, and Kagoshima University. RIG is not available in Japan. Two human rabies vaccines are approved by PMDA, namely, Rabipur (GSK) and Inactivated Tissue Culture Rabies Vaccine (KM Biologics CO., Ltd.).

There have been ongoing discussions about the mandatory annual vaccination of domestic dogs. The current vaccination schedule can provide dogs with protective antibody levels [241]. Although the official data report coverage of over 70%, the true coverage could be lower because of unregistered and unvaccinated dogs. While a web-based survey suggested a high prevalence of immunity among domestic dogs [242], two small-scale studies suggested low protection levels in impounded dogs [243] and juvenile dogs [244]. Furthermore, some researchers argue for the discontinuation of the mandatory vaccination of domestic dogs based on several pieces of evidence. The risk of the re-introduction of a rabid dog to Japan is remarkably low [245, 246]. Even when a rabid dog enters Japan, it is unlikely to cause large outbreaks [247], partly because the probability for a dog to have contact with other dogs is low in Japan [248]. In that situation, the implementation of mandatory vaccination was shown to be economically inefficient [249, 250].

### Scabies

#### Epidemiology

Scabies is a skin infestation caused by a scabies mite or *Sarcoptes scabiei* var. *hominis*. In Japan, scabies outbreaks have been regularly reported. Most outbreaks occur in hospitals, nurseries, and care homes [91–98]. There is no nationwide epidemiological data, but a report estimated the annual number of cases to be between 80,000 and 150,000 [90].

#### Public health response

No legal framework responds to scabies. No mandatory reporting system has been established but hospitals and facilities are encouraged to report outbreaks to local health authorities. The Japanese Dermatological Association published the latest guideline for the diagnosis and treatment of scabies in 2015 [251]. The accuracy of diagnosis depends on dermatologists’ ability to identify *S. scabiei* using dermoscopy or microscopy. Among WHO-recommended medicines, only ivermectin is approved by PMDA for treatment of scabies. Benzyl benzoate and permethrin are not approved by PMDA. Benzyl benzoate has been used for a long time for treatment of scabies in Japan, although this unapproved medicine is not listed as the first-line topical treatment [251]. Besides, phenothrin lotion and topical sulfur preparation are approved by PMDA in Japan.

### Schistosomiasis

#### Epidemiology

*Schistosoma japonicum*, one of the causative agents of schistosomiasis, was once endemic to Japan but its transmission to humans has been considered to be interrupted since 1996 [252]. In recent years, some obsolete cases have been reported with egg embolism and old granuloma, as a consequence of past infections [253–255]. Imported cases of schistosomiasis have been reported mainly among Asian immigrants and Japanese travelers infected in endemic countries, such as China [99], the Philippines [100–104], and some African countries [105–107]. Schistosomes are continuously brought to Japan; however, the risk of domestic schistosome transmission is considerably low because the vector snail of *S. japonicum*, *Oncomelania hupensis nosophora*, is distributed only in Kofu Basin and Obitsu River Basin [252, 256–258] and vectors of other schistosomes have not been found in Japan.

#### Public health response

No legal framework responds to schistosomiasis. Serological testing for diagnosis of *S. japonicum* is available at Miyazaki University upon request from physicians (http://www.med.miyazaki-u.ac.jp/parasitology/detail.htm). The WHO-recommended first-line drug, praziquantel, is approved by PMDA, but it is available for off-label use only.

### Snakebites

#### Epidemiology

Among approximately 20 venomous snakes inhabiting Japan [259, 260], *Gloydius blomhoffii* (or mamushi) is the most epidemiologically important viper. Annually, 1,000–3,000 cases of mamushi bites are reported, including approximately 10 fatal cases [108–110]. The mortality rate of mamushi bites is estimated to be 0.07–0.20% [110, 261]. *Rhabdophis tigrinus* (or yamakagashi) is also widely distributed in Japan. While less than one case of yamakagashi bites is annually recorded on average, the in-hospital mortality rate can be 10–11% [262, 263]. *Gloydius tsushimaensis* only inhabits Tsushima Island in Nagasaki Prefecture, where 72 cases of snake bites by this species were reported between 2005 and 2018, including one death [264]. In the Nansei Islands in Okinawa and Kagoshima Prefectures, five species belonging to the *Protobothrops* genus (or habu) are responsible for snake bites. Okinawa Prefecture alone recorded around 100 cases of habu bites annually between 2012 and 2014, but no fatal case was reported [265–267]. A rare case of snake bite by an illegally-kept eastern green mamba (*Dendroaspis angusticeps*) was also reported [268].

Case reports describe various clinical conditions caused by mamushi bites, including death [111], acute renal failure [112–114], cerebral infarction [115], osteomyelitis [116], thrombocytopenia [117], compartment syndrome [118], diplosia [119], and limited range of motion in hand fingers [120]. Some pediatric cases of mamushi bites are also reported [121–125]. Other case reports describe yamakagashi bites causing disseminated intravascular coagulation [126] and habu bites inducing acute compartment syndrome [127]. Snakebites are a serious public health concern in Japan.

#### Public health response

No legal framework responds to snakebites. There is no national surveillance system, except for Okinawa and Kagoshima Prefectures reporting the number of habu bites. In Japan, no diagnostic kits are available for clinicians to confirm snake-venom poisoning, requiring clinicians to make a clinical diagnosis including the identification of snake species [269]. Japan Snake Center (https://www.snake-center.com/diagnosis) offers consultations to health care providers. For treatment, no snake antivenom is specified by WHO. Freeze-dried antivenoms for mamushi and habu bites are approved by PMDA. Yamakagashi antivenom is available only for clinicians participating in a clinical study [270]. Many local governments publish lists of hospitals that store mamushi antivenom. Despite the availability of antivenoms, whether and when to administer antivenoms has not been standardized in Japan [271]. After a review article concluded that antivenom is the definitive therapy for severe cases [269], some agree that antivenoms should be administered to severe cases [117] or as early as possible [272], but others argue that it is difficult to establish a consistent treatment approach [273]. In addition, several case reports suggest that traditional herbal medicines were effective in some local lesions [274–277]. There is no consensus among physicians about treatment of snakebites in Japan.

### Soil-transmitted helminthiases

#### Epidemiology

Soil-transmitted helminthiases are a group of chronic intestinal diseases caused by several species of helminths. This group includes ascariasis, ancylostomiasis, necatoriasis, trichuriasis, and strongyloidiasis. The annual cases of soil-transmitted helminthiases have decreased in the last decades, except for strongyloidiasis, which has been constantly found in Japan [129, 278].

Strongyloidiasis is mainly caused by *Strongyloides stercoralis*, which is transmitted from the contaminated soil to humans through the skin. Distinctive characteristics of this parasite are autoinfection that can persist and replicate within a host for decades. It potentially leads to life-threatening events such as hyperinfection syndrome and disseminated strongyloidiasis, particularly in immunocompromised patients. In Japan, strongyloidiasis cases are reported nationwide but are more likely to be found in people over 50 years of age, especially in subtropical areas of Okinawa and Kagoshima Prefectures [129]. The prevalence in endemic areas is estimated to reach 18.7% [130]. At least 15 strongyloidiasis cases were estimated to be diagnosed per year in Japan [129].

Strongyloidiasis has been reported particularly among patients with other diseases and conditions, including adult human T-cell lymphotropic virus type 1 infection, meningitis, solid organ malignancy, rheumatic diseases, type 2 diabetes mellitus, steroid use, and organ or bone marrow transplant [279–285]. These cases suggest that the use of immunosuppressive drugs in asymptomatic carriers of *S. stercoralis* may be associated with hyperinfection syndrome and disseminated strongyloidiasis. An imported case from Cambodia is documented in which the patient was co-infected with strongyloidiasis and extrapulmonary tuberculosis [131].

Ascariasis, ancylostomiasis, necatoriasis, and trichuriasis, were endemic to Japan but the transmission of these helminths to human beings was considered to be almost eliminated by 1975 [286]. Nowadays, infections with these helminths are sporadically reported, including presumably imported cases [128] and a case of multiple infections with *Ascaris lumbricoides*, *Trichuris trichiura*, and *Necator americanus* in an 84-year-old woman [132]. A clinical testing facility continues to detect a few ascariasis cases between 2000 and 2018 [133], but it is unknown whether these cases indicate recent *Ascaris* transmission. In the late 2010s, a school-based study detected no infection in five prefectures, suggesting that transmission has become rare even in the areas where high prevalence was previously reported [278], but it is not clear whether transmission of *A. lumbricoides*, *Ancylostoma duodenal*, *N. americanus*, and *T. trichiura* is eliminated in Japan.

#### Public health response

No legal framework responds to soil-transmitted helminthiases. The Tokyo Health Service Association (https://www.yobouigaku-tokyo.or.jp/) has offered parasite screening tests to long-term overseas residents upon their return to Japan since 1987 [287]. Fecal examinations for the diagnosis of *A. lumbricoides*, *T. trichiura*, *A. duodenale*, and *N. americanus* infections are widely accessible at local hospitals and clinics, particularly through commercial laboratories. As for WHO-recommended medicines, pyrantel is approved by PMDA for treatment of ascariasis, ancylostomiasis, and necatoriasis. Mebendazole and ivermectin are also approved for treatment of trichuriasis and strongyloidiasis, respectively. Albendazole is approved but available for off-label use only. Levamisole is not approved in Japan.

### Taeniasis and cysticercosis

#### Epidemiology

Taeniasis is an intestinal infection caused by three species of zoonotic cestode: *Taenia solium*, *Taenia saginata*, and *Taenia asiatica*. Only *T. solium* causes serious conditions known as neurocysticercosis. This review focused on cysticercosis caused by *T. solium*. *T*. *solium* is not considered to be transmitted to humans in Japan because most of the cysticercosis cases are imported or related to travel histories to endemic areas including Nepal, India, and Cambodia [288–293]. However, a literature review [294] identified 39 cysticercosis cases that were found between 1994 and 2010, including seven Japanese nationals without oversea travel histories. This evidence suggests that *T. solium* domestic transmission cannot be ruled out. The possibility of *T. solium* domestic transmission is further supported by the outbreak of human taeniasis due to *T. asiatica* in Tokyo in 2010, as *T. solium* and *T. asiatica* share the same transmission pathways, such as raw pork meat consumption [295].

#### Public health response

*T. solium* infection is reportable as food poisoning under the Food Sanitation Act. Cysticercosis is not covered by the Infectious Diseases Control Law. In Japan, all domestic animals, such as pigs and cows going into the food chain are mandatorily inspected before harvesting (carcass inspection) under the Slaughterhouse Act and the Act on Domestic Animal Infectious Diseases Control. Molecular confirmation of histopathologic specimens is highly useful for the diagnosis of cysticercosis and is available in NIID. Neurocysticercosis is usually suspected due to neurological symptoms, abnormal findings in brain imaging, and the travel history to endemic areas. Serological tests are available at Miyazaki University. The WHO-recommended two anthelmintics, namely, albendazole and praziquantel, are approved by PMDA but available for off-label use only. Niclosamide is not available in Japan.

### Trachoma

#### Epidemiology

Trachoma is a disease of the eye caused by infection with the bacteria, *Chlamydia trachomatis*. No case report was found in our review. Trachoma was frequently reported in the 1950s and 1960s and, to our best knowledge, the last case was reported in 1984 [296]. Meanwhile, other forms of infection with *C. trachomatis*, such as chlamydial conjunctivitis, chlamydial infection of the genital tract, and chlamydia pneumonia are commonly reported in Japan. For instance, a sentinel survey reported 144−887 cases of genital infections by *C. trachomatis* between 2010 and 2018 [297].

#### Public health response

No legal framework responds to trachoma. Laboratory tests for diagnosing infection with *C. trachomatis*, such as PCR or antigen tests, are widely available in Japan. The WHO-recommended antibiotics, azithromycin, and tetracycline are approved by PMDA.

### Yaws

#### Epidemiology

Yaws is a chronic bacterial disease caused by infection with *Treponema pallidum* susbp. *pertenue*. Our review found no evidence of yaws in Japan.

#### Public health response

No legal framework responds to yaws. No hospitals and laboratories offer to test for yaws. However, serological testing of yaws can be substituted by testing for syphilis, and this is widely available in Japan. Azithromycin and benzathine penicillin are recommended by WHO for treatment and are approved by PMDA but available for off-label use only.

## Discussion

To our knowledge, this is the first study that summarized the current epidemiology of and public health responses to the 20 NTDs in Japan. In recent years, NTDs are barely regarded as contemporary public health problems in Japan because we experience only a limited number of cases and/or in limited settings as shown in this study. For several NTDs, such as lymphatic filariasis, rabies, schistosomiasis, leprosy, trachoma, and some soil-transmitted helminthiases, this has been largely a result of our successful disease control programs during the 20th century [240, 298–303]. In addition, Japan has undergone substantial economic development and social changes during this time, which in part, may have contributed to controlling NTDs within the country. However, our review showed evidence that we still have cases of NTDs and the burden from this set of diseases in Japan. For instance, alveolar echinococcosis, Chagas disease, chromoblastomycosis, dengue, foodborne trematodiases (excluding opisthorchiasis), scabies, and strongyloidiasis have been transmitted within Japan as well as imported from other countries recently (Table 1).

In the context of global health, high-income countries such as Japan are expected to play the role of donor countries to combat NTDs in low- and middle-income countries [304, 305]. Japan and other high-income countries should recognize the need to address NTDs within their own countries in addition to their global roles. With globalization and the rapid movement of people, cases of NTDs can be seen anywhere, and thus, it is indeed becoming more and more of a global problem than ever before. NTD patients in high-income countries, whether a locally acquired or an imported case, should not be left behind in the global efforts to end NTDs.

Hotez pointed out that NTDs affect the poor living in wealthy countries [5]. While we did not find any study analyzing the distribution of NTD burdens by income levels in Japan, we found that some diseases mainly affect immigrants, who tend to live with economic vulnerabilities. For example, Chagas disease almost exclusively affects Latino communities in Japan. Similarly, cystic echinococcosis, cysticercosis, leishmaniasis, leprosy, lymphatic filariasis, and schistosomiasis are largely observed among immigrants. These findings provide the critical implication that Japan should pay additional attention to imported NTDs. As we embrace globalization in Japan, we are seeing a growing number of foreign residents in the country. Just in the past decade, it increased from 2.0 million to 2.7 million between 2012 and 2021 [306]. Addressing the health needs of immigrants is important to build an equitable health system since immigrants in Japan tend to experience barriers to health care and poorer health outcomes [307, 308].

Not surprisingly, we found a paucity of epidemiological data, even for the NTDs that are domestically transmitted in Japan, such as foodborne trematodiases and mycetoma (Table 1). With the currently available data, it is not possible to understand the accurate burden of NTDs. The government reported that the average number of NTD patients in Japan was 340 per year through the Japan SDG Action Platform [11]. However, our study showed that this number seems to be grossly underestimated. There were at least 80,000 people who required treatment for scabies alone each year [90]. More population-level surveillance data along with clinical case reports are needed to better understand the epidemiology of NTDs in Japan. Furthermore, surveillance of NTDs in a high-income country like Japan may help improve the understanding of the situation of NTDs in other countries. For example, while laboratory testing for anti-microbial resistance is unavailable in many leprosy endemic countries, a recent epidemiological study in Japan could report cases of anti-microbial resistant strains of *M. leprae* coming from the Philippines [303]. Likewise, a case of Buruli ulcer with the same strain as that of Japan (*M. ulcerans* subsp. *shinshuense*) was reported from the Netherlands in an immigrant from China [309]. *M. ulcerans* subsp. *shinshuense* is currently known as a strain domestic to Japan, but this case report presented the possibility that it may be a strain affecting more countries in Asia. These examples underpin the importance and the need for more global surveillance of NTDs, involving high-income countries.

The levels of public health response to NTDs highly varied by disease in Japan (Table 1). For surveillance, mandatory reporting is established only for echinococcosis, rabies, dengue, and chikungunya under the Act on the Prevention of Infectious Diseases. Two prefectures have set regulations to count snakebites by habu. There is no law or regulation for the other NTDs to be reported to government agencies. For control programs by government bodies, national schemes exist for rabies, dengue, and chikungunya. Echinococcosis is controlled by the Hokkaido Prefectural Government. For diagnosis, we could identify at least one laboratory that offers confirmatory testing for all NTDs except for dracunculiasis, human African trypanosomiasis, onchocerciasis, and yaws. Despite the availability of laboratories, physicians’ capacities to suspect NTDs and send samples to laboratories would require strengthening. In terms of the availability of medicines, none of the WHO-recommended medicines is approved in Japan for Chagas disease and fascioliasis. For other diseases, at least one of the WHO-recommended medicines is approved; however, drugs for cysticercosis, opisthorchiasis, human African trypanosomiasis, onchocerciasis, schistosomiasis, and yaws are available only for off-label use and patients can be required to pay all costs of treatment and related services.

Based on the findings from this review, we developed some recommendations on a way forward to advance the research and control agenda for each NTD (Table 3). A group discussion was used to discuss and reach a consensus. The discussion did not involve any assessment of the ethical, economic, and operational aspects of these recommendations, and thus, we do not intend to provide instruction about which recommendations should be prioritized. In addition to these disease-specific recommendations, we also identified three policy issues across disease areas as discussed below.

**Table 3:** Authors’ recommendations to advance research or control agenda for NTDs in Japan.

NTD	Recommendations
Buruli ulcer	♦ Educate dermatologists and other physicians from relevant fields to promote early diagnosis and treatment. Diagnosis of Buruli ulcer is available in Japan but requires physicians’ ability to suspect the disease from the skin conditions. ♦ Expand a physician-researcher network to improve reporting of Buruli ulcer. The current reporting system is managed by the Leprosy Research Center of the NIID and the expert group but is based on voluntary reporting from physicians leading to under-reporting.
Chagas disease	♦ Investigate access to diagnosis and treatment in Japan. Two unapproved drugs, benznidazole and nifurtimox, can be obtainable from WHO, but whether patients have access to them is not clearly understood. ♦ Establish screening programs to detect infected persons before they develop symptoms. For instance, serological testing can be integrated into workplace medical checkups or prenatal checkups in municipalities with large Latino populations.
Cysticercosis	♦ Educate physicians to suspect cysticercosis when immigrants from endemic areas present central nervous system infections. Domestic transmission can be triggered by infected international travelers or agricultural workers. ♦ Maintain the mandatory inspection of meat.
Dengue and chikungunya	♦ Monitor outbreaks of dengue and a new case of domestic transmission of chikungunya. ♦ Pay attention to the trend of imported cases from overseas and continue to monitor mosquitoes in Japan to prevent new outbreaks.
Echinococcosis	♦ Investigate why transmission of *E. multilocularis* to humans is evidenced only in Hokkaido. The risk of its spread into the main islands was investigated in 2003 [310] but needs to be updated.
Foodborne trematodiases	♦ Establish a monitoring system to detect hot spots of clonorchiasis, fascioliasis, and paragonimiasis, for instance, by regular stool tests for school children in areas known to be endemic in the past. Since the transmission patterns of these diseases have varied due to increased immigration, changes in dietary habits, and heavy rains/flooding, it is necessary to monitor possible epidemic areas to understand the risks.
Leishmaniasis	♦ Educate physicians to address sporadically imported cases. ♦ Consider adopting topical paromomycin for treatment of cutaneous leishmaniasis [311] to expand treatment options.
Leprosy	♦ Educate and organize dermatologists to manage leprosy patients. The repeal of the Leprosy Prevention Law in 1996 allowed patients to be managed at general hospitals or clinics, but dermatologists in general are not experienced in managing leprosy patients. ♦ Surveillance of leprosy is continued at the LRC/NIID and the expert group, without any legal basis. The LRC can consider establishing integrated surveillance of skin NTDs that exist in Japan, to ensure the sustainability of the leprosy surveillance program.
Lymphatic filariasis	♦ Investigate whether patients found in Japan have access to treatment.
Mycetoma, chromoblastomycosis, and other deep mycoses	♦ Educate dermatologists to promote timely diagnosis. ♦ Build a consensus among clinical researchers to report mycetoma, chromoblastomycosis, sporotrichosis, and paracoccidioidomycosis following international definitions of these diseases.
Rabies	♦ Consider revising the mandatory annual vaccination of domestic dogs, as this strategy is suggested to be no more cost beneficial. The risk of having rabies introduced to and spread in Japan is considerably low. ♦ Secure access to rabies immune globulins (RIG). Since RIG is not approved in Japan, it should probably be provided through a research group.
Scabies	♦ Establish outbreak surveillance and control systems, since outbreaks of scabies have been regularly observed in Japan. ♦ Evaluate the efficacy and safety of crotamiton and benzyl benzoate for treatment of scabies and consider obtaining PMDA approval.
Schistosomiasis	♦ Raise awareness and educate physicians to promote diagnosis of schistosomiasis, since obsolete cases and imported cases have been regularly found in Japan. ♦ Consider obtaining PMDA approval of praziquantel for treatment of schistosomiasis, as this drug is only available for off-label use.
Snakebites	♦ Accumulate clinical cases to get the currently proposed treatment guideline updated and accepted by the medical community. Although mamushi and habu antivenoms are approved by PMDA, there is no consensus in the medical community on whether and when to administer antivenoms. ♦ Guarantee government support for the production of yamakagashi antivenom. Yamakagashi antivenoms are voluntarily produced by a research group at the Japan Snake Center. ♦ Establish information systems at all prefectural governments to collect and publish the number of mamushi bites, just as Okinawa and Kagoshima Prefectures do for habu bites. Although mamushi bites are frequently reported from all over Japan, there is no national surveillance system. ♦ Collect data about the use of mamushi antivenoms to understand the demand for sustainable production and storage of antivenoms.
Soil-transmitted helminthiases	♦ Consider testing for Strongyloides infections to avoid hyper-infection syndrome and disseminated strongyloidiasis, particularly in patients over 50 years of age with a history of residence in Kagoshima and Okinawa Prefectures who will be treated with immunosuppressive drugs, anticancer drugs, or steroids.

Note: We have no specific recommendations for dracunculiasis, human African trypanosomiasis, onchocerciasis, trachoma, and yaws. We consider that these diseases are not Japan’s priority because we did not find any evidence of domestic transmission nor imported cases.

First, we argue that some NTDs require a legal basis for better public health response. Our review suggests that the government provides organized responses to the NTDs that are already covered by the Infectious Disease Control Law (Table 1). This law classifies infectious diseases into five categories based on each disease’s infectivity and severity. It then defines the roles of stakeholders to organize reporting, prevention, and healthcare provision. In general, diseases that are considered highly infective or severe, such as Ebola virus disease, tuberculosis, and cholera, are classified into Class I, II, or III. Other infectious diseases whose infectivity or severity are not considered so problematic are classified into Class IV or V (Table 4). Although NTDs are not highly infective or severe diseases, we argue that some NTDs are yet eligible for Class IV or V, as Table 4 explains. Establishing a legal basis for these NTDs can be a critical step to advancing their surveillance and control in Japan. In addition, snakebite envenoming would need similar legal support outside of the Infectious Disease Control Law.

**Table 4:** Infectious Disease Control Law and NTDs that can be designated by this law.

How are diseases classified by the law?				Which NTD can fit the law’s classification?	
Class	Definition of the class by the law^1^	Examples of diseases already designated by the law	Interpretation of classification by the Ministry of Health, Labour, and Welfare^2^	NTDs to be designated	Rationale
Class IV	[A]ny known infectious disease [. . .] which is transmissible to human beings through animals or animal corpses, food or drink, clothing, bedding, or other physical items and which is likely to affect the health of citizens (Article 6- [5]-(xi))	Hepatitis E, hepatitis A, yellow fever, Q fever, rabies, anthrax, avian influenza, botulism, malaria, and tularemia	Diseases that are not classified into Class I, II, or III [i.e., infectivity and severity of illness is relatively low] and that are mainly transmitted to humans via animals and other items.	Scabies	Mites are transmissible to human beings through clothing and bedding. Scabies is widely spread in Japan and its outbreaks are regularly reported. More than 80,000 incident cases per year are estimated. The government should standardize and guide response to scabies outbreaks, such as mite control.
Class V	[A]ny known infectious disease (excluding Class IV Infectious Diseases) [. . .] which is likely to affect the health of citizens (Article 6- [6]-(ix))	Influenza, viral hepatitis, cryptosporidiosis, acquired immunodeficiency syndrome, genital chlamydia infection, syphilis, measles, and methicillin-resistant Staphylococcus aureus infection	Diseases whose surveillance data need to be provided to the public and healthcare workers.	Buruli ulcer	It is transmitted within Japan, but its surveillance is voluntarily conducted by NIID. Dermatologists should be informed for better case findings.
				Chagas disease	3,000–4,500 infected people are estimated to live in Japan, with a few cases of mother-to-child transmission. No surveillance is in place. The public and healthcare workers should be informed, particularly in Latino communities.
				Clonorchiasis	Imported and domestic cases are confirmed in Japan but no surveillance is in place. The public and healthcare workers should be informed, particularly in areas where domestic transmission has been found.
				Cysticercosis	Cases are reported among immigrants and Japanese travelers. Domestic transmission cannot be ruled out. No surveillance is active for this disease. The public and healthcare workers should be informed for better case findings.
				Fascioliasis	This disease is endemic in Japan with some imported cases found. No surveillance is in place. The public and healthcare workers should be informed, particularly in areas where domestic transmission has been found.
				Leprosy	Imported cases and cases from past infections are being found in Japan. Its surveillance is voluntarily conducted by NIID. Dermatologists should be informed for better case findings.
				Mycetoma, chromoblastomycosis, and sporotrichosis	These diseases are endemic in Japan. No surveillance is in place. The public and healthcare workers should be informed for better case findings, particularly in epidemic areas.
				Paragonimiasis	This disease is endemic in Japan with some imported cases found. No surveillance is in place. The public and healthcare workers should be informed, particularly in areas where domestic transmission has been found.
				Strongyloidiasis	Imported and domestic cases are confirmed in Japan. At least 15 cases are found per year, but no surveillance is in place. Healthcare workers, particularly those who attend to immunosuppressed patients, should be informed about better treatment.

1. English translation of the articles is extracted from the Japanese Law Translation database (http://www.japaneselawtranslation.go.jp/) provided by the Ministry of Justice, Japan.

2. Reference material presented at the third meeting of the Health Sciences Council (Taskforce for Infectious Diseases) held on March 14, 2014. Available from https://www.mhlw.go.jp/file/05-Shingikai-10601000-Daijinkanboukouseikagakuka-Kouseikagakuka/0000040509.pdf

Next, we propose that the government should be more responsible for securing the provision of NTD-related health products that are not commercially available in Japan. For instance, diagnostics for Chagas disease, foodborne trematodiases, lymphatic filariasis, and schistosomiasis are produced or purchased by researchers doing investigations on each disease. Similarly, the yamakagashi antivenom is produced only by a research group at the Japanese Snake Center. In addition, medicines for Chagas disease and fascioliasis are not approved in Japan, although they are listed as essential medicines by WHO. Currently, the Research Group of Tropical Diseases and Parasitic Diseases stores triclabendazole for fascioliasis. These NTD products can become unavailable when research funds are discontinued. The government can allocate a budget for securing NTD products, or more preferably, can create a new office to address this problem within public institutions, such as the National Institute of Infectious Diseases or the National Center of Global Health and Medicine.

Finally, we emphasize the importance of addressing the unaffordability of treatment using unapproved drugs. In Japan, treatment of Chagas disease and fascioliasis requires the use of unapproved drugs (Table 1). Unapproved drugs are not covered by health insurance and, in the worst case, patients can be required to fully pay for all health services received. To ensure patient affordability, obtaining PMDA approval is the standard way so that these drugs can be covered by insurance. However, this option can be possible only when manufacturers make decisions to apply for PMDA approval. Alternatively, patients can receive unapproved drugs for free when they are enrolled in clinical trials. Although the main purpose of a clinical trial is not to protect patients from financial risks, this seems to be the only realistic way to ensure the affordability of unapproved medicines. At the time of this writing, a clinical trial of triclabendazole for fascioliasis is recruiting patients [312]. Launching clinical trials would also be recommended for benznidazole and nifurtimox.

Our review involves several limitations. First, our search strategy may have been unable to find some relevant publications, particularly when the Japanese databases register papers using different disease names than the controlled terms that we adopted as search terms. Second, NTD cases are likely to be underdiagnosed in Japan, which leads to an underestimation of the burden of NTDs. Even if diagnosed, cases usually remain undocumented when medications are not available. Third, our review may involve our own bias. Each author had more knowledge and expertise in one or a few disease areas, which could influence the literature search and writing processes. To minimize this potential bias, we developed the search protocol together. Each author read through the manuscript and any disagreements were resolved through group discussion. Lastly, our discussions of public health response assessed the availability of laboratory testing and medicines, but we could not fully assess whether they are accessible to patients. Further research on access to NTD health technologies in Japan is required.

In conclusion, our review found that 15 out of 20 NTDs can be considered important public health concerns in Japan. Among these 15 NTDs, some are transmitted within Japan and others are imported from endemic countries. Japan’s health systems address only a portion of 15 NTDs, particularly five diseases that are targeted by the Infectious Disease Control Law. NTD control often requires support from the public sector. Strengthening government support for nationwide surveillance of NTDs as well as for ensuring the availability and affordability of NTD-related health technologies, can be a critical step to advancing NTD control in Japan. Our illustration of NTDs in Japan also implies that even high-income countries should study, recognize, and address NTDs to reduce health disparities within their own countries.

## Supporting information

S1 Document Results of literature search with three databases. (XLSX)

S2 Document List of selected articles by diseases. (DOCX)

S3 Document Map of Japan showing geographical locations of prefectures and regions that are mentioned in this review. (DOCX)
