# Supplementary material for: The current status of neglected tropical diseases in Japan: A scoping review
Source: PLoS Negl Trop Dis. 2024 Jan 2;18(1):e0011854. doi: 10.1371/journal.pntd.0011854 (PMC10786391; doi:10.1371/journal.pntd.0011854)
Supplement: S2 Document — (DOCX) [file pntd.0011854.s002.docx]

Supplementary Document 2: List of selected articles by diseases

**Buruli ulcer (12)**

1. Hasumi J, Hukazawa C, Hoshino T, Hamada H, Ohkusu K. A case of cervical skin ulcers by *Mycobacterium ulcerans* subsp. *shinshuense*. The Journal of Pediatric Infectious Disease and Immunology. 2012;23(4):383–8.

2. Kabata D, Tanizaki H, Arakawa A, Tanioka M, Takakura S, Okusu K, et al. A case of Buruli ulcer caused by *Mycobacterium ulcerans* subsp. *shinshuense*. The Japanese Journal of Dermatology. 2011;121(14):3337–42.

3. Luo Y, Degang Y, Ohtsuka M, Ishido Y, Ishii N, Suzuki K. Detection of *Mycobacterium ulcerans* subsp. *shinshuense* DNA from a water channel in familial Buruli ulcer cases in Japan. Future Microbiol. 2015;10(4):461–9.

4. Nakanaga K, Hoshino Y, Yotsu RR, Makino M, Ishii N. Nineteen cases of Buruli ulcer diagnosed in Japan from 1980 to 2010. J Clin Microbiol. 2011 Nov;49(11):3829–36.

5. Nakanaga K, Yotsu RR, Hoshino Y, Suzuki K, Makino M, Ishii N. Buruli ulcer and mycolactone-producing mycobacteria. Jpn J Infect Dis. 2013;66(2):83–8.

6. Ohtsuka M, Kikuchi N, Yamamoto T, Suzutani T, Nakanaga K, Suzuki K, et al. Buruli ulcer caused by *Mycobacterium ulcerans* subsp *shinshuense*: a rare case of familial concurrent occurrence and detection of insertion sequence 2404 in Japan. JAMA Dermatol. 2014 Jan;150(1):64–7.

7. Sugawara M, Ishii N, Nakanaga K, Suzuki K, Umebayashi Y, Makigami K, et al. Exploration of a standard treatment for Buruli ulcer through a comprehensive analysis of all cases diagnosed in Japan. J Dermatol. 2015 Jun;42(6):588–95.

8. Umebayashi Y, Manabe M, Nakanaga K, Ishii N. A case report of BU in Akita Prefecture. Practical Dermatology. 2013;35(7):669–72.

9. Wakai S, Yajima M. Histopathological examination of Buruli ulcer (Mycobacterium ulcerans disease) in surgically removed skin. Japanese Journal of Leprosy. 2011;80(3):269–74.

10. Yotsu RR, Murase C, Sugawara M, Suzuki K, Nakanaga K, Ishii N, et al. Revisiting Buruli ulcer. J Dermatol. 2015 Nov;42(11):1033–41.

11.Yotsu RR, Nakanaga K, Hoshino Y, Suzuki K, Ishii N. Buruli ulcer and current situation in Japan: A new emerging cutaneous Mycobacterium infection. The Journal of Dermatology. 2012 Jul;39(7):587–93.

12. Yotsu RieR. The viewpoint of Skin NTDs (neglected tropical diseases) and the response in Japan. Japanese Journal of Clinical Dermatology. 2018;72(5):31–6.

**Chagas disease (27)**

1. Coura JR. The main sceneries of Chagas disease transmission. The vectors, blood and oral transmissions--a comprehensive review. Mem Inst Oswaldo Cruz. 2015;110(3):277–82.

2. Gascon J, Bern C, Pinazo MJ. Chagas disease in Spain, the United States and other non-endemic countries. Acta Trop. 2010;115(1–2):22–7.

3. Gascon J, Vilasanjuan R, Lucas A. The need for global collaboration to tackle hidden public health crisis of Chagas disease. Expert Rev Anti Infect Ther. 2014;12(4):393–5.

4. Hirayama K. Preparedness for Chagas disease. Nihon Iji Shimpo (Japan Medical Journal). 2014;(4723):47.

5. Hirayama K. Tropical diseases in Japan - trypanosomiasis and leishmaniasis. Nihon Iji Shimpo (Japan Medical Journal). 2019. p. 22–3.

6. Ikeno Y, Miuara S, Maeda T, Imai K, Sayama Y, et al. A case of chronic Chagas disease patient who was treated with benznidazole and kept remission for five years. Vol. 30, Nihon Rinsyo Kiseichu Gakkaishi (Clinical Parasitology). 2019. p. 62–4.

7. Ikeno Y, Miura S, Sayama Y, Fukushima F, Sakio H, et al. A case of Chagas disease patient who was treated with benznidazole. Vol. 26, Nihon Rinsyo Kiseichu Gakkaishi (Clinical Parasitology). 2015. p. 90–2.

8. Imai K. Chagas disease. Rinsho To Biseibutsu (Clinical Microbiology). 2014;41(4):353–7.

9. Imai K, Maeda T, Sayama Y, Mikita K, Fujikura Y, Misawa K, et al. Mother-to-child transmission of congenital Chagas disease, Japan. Emerg Infect Dis. 2014;20(1):146–8.

10. Imai K, Maeda T, Sayama Y, Osa M, Mikita K, Kurane I, et al. Chronic Chagas disease with advanced cardiac complications in Japan: Case report and literature review. Vol. 64, Parasitology International. 2015. p. 240–2.

11. Imai K, Misawa K, Osa M, Tarumoto N, Sakai J, Mikita K, et al. Chagas disease: a report of 17 suspected cases in Japan, 2012-2017. Vol. 47, Tropical Medicine and Health. 2019. p. 1–5.

12. Kato Y, Kano S, Ohnishi K. Infectious diseases: progress in diagnosis and treatment. Topics: III. Emerging and re-emerging infectious diseases; 4. Emerging parasitic diseases. Nihon Naika Gakkai Zasshi. 2012;101(11):3162–7.

13. Maeda T, Nanun M, Sayama Y, Misawa K, Imai K, et al. Two cases of Chagas disease with benznidazole treatment. Vol. 24, Rinsho To Biseibutsu (Clinical Microbiology). 2013. p. 33–6.

14. Miura S. Infectious diseases - Chagas disease and its current situation in Japan. Nihon Iji Shimpo (Japan Medical Journal). 2018;(4928):55–6.

15. Miyanaga D, Tama N, Amaya N, Sarasawa K, Ikeda H, Fukuoka Y, et al. A case of Chagas cardiomyopathy with severe heart failure and ventricular tachycardia. Shinzo (Heart). 2019;51(9):932–8.

16. Momose S. A case of blood donation by a person positive to Chagas disease. Rinsho Kensagaku Zasshi (Medical Technology). 2014;42(8):766–9.

17. Momose S. Safety measures for blood products. Vol. 44, Kansensyo (The Infection). 2014. p. 97-102,91-92.

18. Nagumo M, Maeda T, Imai K, Misawa K, Kei M, Fujikura Y, et al. A case report of congenital Chagas’ disease in Japan. Journal of the National Defense Medical College. 2013;38(3):225–30.

19. Nara T. Chagas disease. Igaku No Ayumi (Journal of Clinical and Experimental Medicine). 2015;253(1):119–23.

20. Nara T. Globalization, global warming, and infectious disease control - vector-borne disease, Chagas disease. Vol. 70, Shonika Rinsho (Japanese Journal of Pediatrics). 2017. p. 2267–71.

21. Ono T, Miyahira Y. Current parasitic, insect and animal-borne diseases: 1- imported parastic diseases among children. Shonika Rinsho (Japanese Journal of Pediatrics). 2012;65(3):360–8.

22. Satoh F, Tachibana H, Hasegawa I, Osawa M. Sudden death caused by chronic Chagas disease in a non-endemic country: Autopsy report. Vol. 60, Pathology International. 2010. p. 235–40.

23. Sayama Y, Furui Y, Takakura A, Ishinoda M, Matsumoto C, Taira R, et al. Seroprevalence of Trypanosoma cruzi infection among at-risk blood donors in Japan. Transfusion. 2019;59(1):287–94.

24. Schmunis GA, Yadon ZE. Chagas disease: a Latin American health problem becoming a world health problem. Acta Trop. 2010;115(1–2):14–21.

25. Shintaku M, Takeda S, Miura S, Yutani C, Tsutsumi Y. Chronic Chagastic cardiomyopathy associated with membranoproliferative glomerulonephritis: Report of an autopsy case. Pathol Int. 2020;70(1):47–52.

26. Taira R. Cost-benefit of testing for transfusion-based infectious diseases. Ketsueki Zigyo (Journal of the Society for Japanese Blood Programme). 2018;41(1):73–5.

27. Yasukawa K. Blood donor screening for Trypanosoma cruzi infection in Japan. Transfusion. 2014;54(3):745–6.

**Dengue and chikungunya**

*Dengue (157)*

1. Aoki Y. Case of a 13-year-old girl with dengue fever who developed the disease after participating in an overseas training project [in Japanese]. 11. 2017;2:89–92.

2. Arima Y, Matsui T, Shimada T, Ishikane M, Kawabata K, Sunagawa T, et al. Ongoing local transmission of dengue in Japan, August to September 2014. Western Pac Surveill Response J. 2014 Dec;5(4):27–9.

3. Chaves LF. Globally invasive, withdrawing at home: Aedes albopictus and Aedes japonicus facing the rise of Aedes flavopictus. Int J Biometeorol. 2016 Nov;60(11):1727–38.

4. Churrotin S, Kotaki T, Sucipto TH, Ahwanah NLF, Deka PT, Mulyatno KC, et al. Dengue Virus Type 1 Strain Isolated in Indonesia Shows a Close Phylogenetic Relation with the Strains That Caused the Autochthonous Dengue Outbreak in Japan in 2014. Jpn J Infect Dis. 2016 Sep 21;69(5):442–4.

5. Eiji K. Prevention of mosquito-borne infectious diseases - malaria, dengue fever, chikungunya fever, Zika fever. Shoni Naika. 2017;49(6):813–7.

6. Fukui S, Uehara Y, Fukui-Watanabe Y, Matsuda N, Hisaoka T, Naito T. Laboratory features of the acute-phase of viral infections, including dengue fever. Nihon Byouin Sougousinryou Igakukai Zasshi (Journal of Hospital General Medicine). 2017;12(1):1–6.

7. Fukusumi M, Arashiro T, Arima Y, Matsui T, Shimada T, Kinoshita H, et al. Dengue Sentinel Traveler Surveillance: Monthly and Yearly Notification Trends among Japanese Travelers, 2006-2014. PLoS Negl Trop Dis. 2016 Aug;10(8):e0004924.

8. Furuichi M, Makie T, Honma Y, Isoda T, Miyake S. Laboratory-confirmed dengue fever and chikungunya fever cases at the Narita Airport Quarantine Station in 2013. Jpn J Infect Dis. 2015;68(2):142–4.

9. Furuya H. Estimation of reproduction number and probable vector density of the first autochthonous dengue outbreak in Japan in the last 70 years. Environ Health Prev Med. 2015 Nov;20(6):466–71.

10. Furuya H. Estimating Vector-borne Viral Infections in the Urban Setting of the 2020 Tokyo Olympics, Japan, Using Mathematical Modeling. Tokai J Exp Clin Med. 2017 Dec 20;42(4):160–4.

11. Goto K, Hatakeyama S, Okamoto K, Kitazawa T, Abe K, Moriya K, et al. Dengue hemorrhagic fever in an adult traveler returning to Japan. Intern Med. 2012;51(13):1779–82.

12. Hadano Y, Shirano M, Goto T. Travel-related illness at a tertiary care hospital in Osaka, Japan. Int J Gen Med. 2016;9:355–9.

13. Hamada A, Yamaguchi Y. Preventive measures against dengue fever [in Japanese]. BMSA journal (The Journal of Biomedical Science and Biosafety). 2014;26(1):26–30.

14. Haruki K. Imported infectious diseases: Dengue fever, Malaria [in Japanese]. Dokkyo Journal of Medical Sciences. 2015;42(3):239–45.

15. Hasebe F. Dengue fever, Japanese encephalitis, West Nile fever [in Japanese]. Rinsyo to Biseibutsu. 2014 Jan;41(1):65–71.

16. Hasegawa C. Imported infectious disease [in Japanese]. Gendai Igaku. 2016;64(1):41–7.

17. Hashimoto T, Kutsuna S, Maeki T, Tajima S, Takaya S, Katanami Y, et al. A Case of Dengue Fever Imported from Burkina Faso to Japan in October 2016. Jpn J Infect Dis. 2017 Nov 22;70(6):675–7.

18. Hensyubu. Dengue fever, end the war in accordance with the basics that have remained unchanged for 70 years. 2014 Sep;(4716):9.

19. Hensyubu. Expanding Dengue Fever Transmission. Nihon Iji Shimpo (Japan Medical Journal). 2014 Sep;(4717):14–5.

20. Higa Y, Maekawa Y, Minagawa K, Muto A, Sasaki Hi, Kasai S. Investigation of mosquito density [in Japanese]. Medical Entomology and Zoology. 2020;71(2):73–8.

21. Hirata K, Ogawa T, Fujikura H, Ogawa Y, Hirai N, Nakagawa-Onishi T, et al. Characteristics of health problems in returned overseas travelers at a tertiary teaching hospital in a suburban area in Japan. J Infect Chemother. 2018 Aug;24(8):682–5.

22. Hitani A, Yamaya W, To M, Kano I, Hosono-Honda N, Takasaki T, et al. A Case of Dengue Fever and Subsequent Long-lasting Depression Accompanied by Alopecia in a Japanese Traveler Returning from Bali, Indonesia [in Japanese]. Kansenshogaku Zasshi (The Journal of the Japanese Association for Infectious Diseases). 2015;89(2):279–82.

23. Ishikawa H, Shimogawara R. Risk Assessment of Dengue Autochthonous Infections in Tokyo during Summer, Especially in the Period of the 2020 Olympic Games. Jpn J Infect Dis. 2019 Nov 21;72(6):399–406.

24. Ishikawa H, Shimogawara R, Fueda K. How Did the Dengue Fever Outbreak Progress in Yoyogi Park, Tokyo, in 2014?-Evaluation Based on a Mathematical Model [in Japanese]. Nihon Eiseigaku Zasshi. 2017;72(1):55–65.

25. Ishikawa T, Ikeda H, Okamoto N, Kodama M, Akiyama K, Okamoto Y. Two cases of autoshothonous and imported dengue infection in children [in Japanese]. Showa Gakushi Kaishi. 2017;77(4):455–61.

26. Izumida T, Sakata H, Nakamura M, Hayashibara Y, Inasaki N, Inahata R, et al. A False Positive Dengue Fever Rapid Diagnostic Test Result in a Case of Acute Parvovirus B19 Infection. Intern Med. 2016;55(10):1379–82.

27. Jeong YE, Lee WC, Cho JE, Han MG, Lee WJ. Comparison of the Epidemiological Aspects of Imported Dengue Cases between Korea and Japan, 2006-2010. Osong Public Health Res Perspect. 2016 Feb;7(1):71–4.

28. Jin T, Ono Y, Shigesawa T, Koike Y, Endo A, Nakamura M, et al. A case of dengue fever episode in Japan, without traveling abroad [in Japanese]. Sapporo city general hospital. 2015 Mar;74(2):265–70.

29. Kaburagi Y, Kaetsu A, Kobori S, Udno Hi, Kikuchi K, Tomari K, et al. Dengue Fever Infection Confirmed with an Epidemiological Survey and Genetic Analysis: The Case was Suspected as SFTS Based on the Primary First Information [in Japanese]. Kansenshogaku Zasshi (The Journal of the Japanese Association for Infectious Diseases). 2016;90(1):88–91.

30. Kajimoto Y, Kitajima T. Clinical Management of Patients with Dengue Infection in Japan: Results from National Database of Health Insurance Claims. Am J Trop Med Hyg. 2020 Jan;102(1):191–4.

31. Kajimoto Y, Kitajima T. Patient and National Economic Burden of Dengue in Japan: Results from Japanese National Claims Database. Am J Trop Med Hyg. 2020 Jun;102(6):1237–43.

32. Kanai Hi, Fujikawa Hi, Sato Hi, Takei Y, Sato T. A female case of dengue fever experienced in a pediatric primary care setting [in Japanese]. Shonika Shinryo (The Journal of Pediatric Practice). 2014;77(6):827–30.

33. Kasai S. Introduction:background of the emergency drill [in Japanese]. Medical Entomology and Zoology. 2020;71(2):65–6.

34. Kasai S, Ugajin T, Shimizu I, Muto A, Sasaki Hi, Ikeda F, et al. Outline of an emergency drill for controlling the mosquito vector of dengue fever held at Shinjuku Gyoen National Garden in 2019 [in Japanese]. Medical Entomology and Zoology. 2020;71(2):67–71.

35. Kato Y. Ebola Epidemic and Dengue Fever in Ending West Africa. INFECTION CONTROL. 2015;24(12):41–4.

36. Katsuta Y. Survey of religious officials’ awareness of mosquito control measures for dengue fever, chikungunya fever, and other vector mosquitoes (in Japanese). Nihon Iji Shimpo (Japan Medical Journal). 2015 Aug;(4764):44–9.

37. Kawada I, Sakamaki F. Two Cases of Dengue Fever after a Family Trip to Thailand [in Japanese]. Kansenshogaku Zasshi (The Journal of the Japanese Association for Infectious Diseases). 2018;92(1):61–4.

38. Kawashima S, Kumagai N, Matsuki T, Kamimura M, Nakayama M, Kanno J, et al. A case of dengue fever with hemorrhage [in Japanese]. Shonika Rinsho (Japanese Journal of Pediatrics). 2014;67(10):1655–9.

39. Kobayashi D, Murota K, Fujita R, Itokawa K, Kotaki A, Moi ML, et al. Dengue Virus Infection in Aedes albopictus during the 2014 Autochthonous Dengue Outbreak in Tokyo Metropolis, Japan. Am J Trop Med Hyg. 2018 May;98(5):1460–8.

40. Kobayashi D, Sasaki T, Isawa H. Detection of Aedes-borne viruses from field-caught mosquitoes and consideration for establishment of persistent DENV transmission cycles in Japan [in Japanese]. Medical Entomology and Zoology. 2020;71(2):85–90.

41. Kobayashi K, Onishi K. Dengue fever [in Japanese]. Shonika Rinsho (Japanese Journal of Pediatrics). 2013;66(7):1493–8.

42. Kobayashi M. Dengue fever: Japan Joins Southeast Asia [in Japanese]. BMSA journal (The Journal of Biomedical Science and Biosafety). 2014;26(4):1–2.

43. Kobayashi M. Mosquito-borne Infectious Diseases: Focus on Dengue Fever [in Japanese]. Dokkyo Journal of Medical Sciences. 2015;42(3):179–85.

44. Kobayashi M, Komagata O, Yonejima M, Maekawa Y, Hirabayashi K, Hayashi T, et al. Retrospective search for dengue vector mosquito Aedes albopictus in areas visited by a German traveler who contracted dengue in Japan. Int J Infect Dis. 2014 Sep;26:135–7.

45. Kojima G. Autochthonous dengue fever imported to England from Japan, 2014. Emerg Infect Dis. 2015 Jan;21(1):182–4.

46. Kondo M, Akachi S, Goto H, Umaoka A, Yamanaka K, Mizutani H. Case of dengue fever missed at the initial screening of an endemic area but developed later in Japan. J Dermatol. 2017 Jul;44(7):847–8.

47. Konishi E. Dengue and Dengue Hemorrhagic Fever and Current Status of Vaccine Development (in Japanese). Nihon Iji Shimpo (Japan Medical Journal). 2012 Jun;(4599):48–9.

48. Konishi E, Kuno G. In memoriam: Susumu Hotta (1918-2011). Emerg Infect Dis. 2013 May;19(5):843–4.

49. Kori M, Awano N, Inomata M, Kuse N, Tone M, Yoshimura H, et al. The 2014 autochthonous dengue fever outbreak in Tokyo: A case series study and assessment of the causes and preventive measures. Respir Med Case Rep. 2020;31:101246.

50. Kurita N, Tada Y, Fukushima S, Yoshikawa M, Hamada A. Level of Knowledge Regarding Dengue Fever and Zika Virus Infection Among the Japanese Travellers [in Japanese]. Kansenshogaku Zasshi (The Journal of the Japanese Association for Infectious Diseases). 2018;92(6):863–8.

51. Kusama Y, Ito K, Tajima S, Kutsuna S. A pediatric case of imported dengue hemorrhagic fever in Japan. J Gen Fam Med. 2017 Dec;18(6):414–7.

52. Kusumi E. Prevention of mosquito-borne infectious diseases - malaria, dengue fever, chikungunya fever, Zika fever [in Japanese]. Jpn J Pediatr Med. 2017;49(6):813–7.

53. Kutsuna S. Clinical Symptoms and Testing/Diagnosis of Dengue Fever to Understand in Preparation for an Epidemic [in Japanese]. Medical Technology. 2015 Jun;43(6):587–91.

54. Kutsuna S. Domestic outbreak of dengue fever. Medical Practice. 2016;33(1):96–8.

55. Kutsuna S. The background of the dengue epidemic in Japan, 2014 and the future countermeasure [in Japanese]. Rinsho to Virus. 2016;44(1):37–9.

56. Kutsuna S. Diagnosis of the dengue fever [in Japanese]. Igaku No Ayumi (Journal of Clinical and Experimental Medicine). 2017;263(13):1229–33.

57. Kutsuna S. Differences of clinical presentations among mosquito-borne diseases, especially in dengue fever, chikungunya fever, and zika virus infection [in Japanese]. Rinsho to Virus. 2017;44(5):221–6.

58. Kutsuna S. Rapid diagnostic test of dengue fever [in Japanese]. Rinsyo to virus. 2017;45(3):102–4.

59. Kutsuna S. Dengue fever [in Japanese]. Sanka to Fujinka. 2018;8(83):955–9.

60. Kutsuna S. Imported infectious disease: Dengue fever [in Japanese]. Medical Practice. 2019;36:282–6.

61. Kutsuna S, Hayakawa K, Kato Y, Fujiya Y, Mawatari M, Takeshita N, et al. Comparison of clinical characteristics and laboratory findings of malaria, dengue, and enteric fever in returning travelers: 8-year experience at a referral center in Tokyo, Japan. J Infect Chemother. 2015 Apr;21(4):272–6.

62. Kutsuna S, Kato Y, Moi ML, Kotaki A, Ota M, Shinohara K, et al. Autochthonous dengue fever, Tokyo, Japan, 2014. Emerg Infect Dis. 2015 Mar;21(3):517–20.

63. Kutsuna S, Kato Y, Ohmagari N. Nineteen cases of autochthonous dengue fever, Tokyo, Japan, 2014 [in Japanese]. Medical Entomology and Zoology. 2015;66(4):207–9.

64. Kuwahara N, Toma T, Miyaji I. Survey study on prevention of imported infectious diseases and dengue fever among Japanese tourists visiting Bali and risk management against infectious diseases [in Japanese]. Nihon Toko igakkai ( Journal of the Japanese Society of Travel and Health). 2010;4(1):13–8.

65. Lee JS, Farlow A. The threat of climate change to non-dengue-endemic countries: increasing risk of dengue transmission potential using climate and non-climate datasets. BMC Public Health. 2019 Jul 11;19(1):934.

66. Liu Y, Lillepold K, Semenza JC, Tozan Y, Quam MBM, Rocklöv J. Reviewing estimates of the basic reproduction number for dengue, Zika and chikungunya across global climate zones. Environ Res. 2020 Mar;182:109114.

67. Manni M, Guglielmino CR, Scolari F, Vega-Rúa A, Failloux AB, Somboon P, et al. Genetic evidence for a worldwide chaotic dispersion pattern of the arbovirus vector, Aedes albopictus. PLoS Negl Trop Dis. 2017 Jan;11(1):e0005332.

68. Matsui T, Kinoshita N, Maeki T, Kutsuna S, Nakamura K, Nakamoto T, et al. Dengue Virus Type 2 Infection in a Traveler Returning from Saudi Arabia to Japan. Jpn J Infect Dis. 2019 Sep 19;72(5):340–2.

69. Matsumoto T. Lessons Learned from the Dengue Fever Epidemic [in Japanese]. Medical Technology. 2014;42(12):1181.

70. Meng Ling Moi. Dengue Fever [in Japanese]. Rinsho byori The Japanese journal of clinical pathology. 2016;(64):1033–43.

71. Meng Ling Moi. Dengue fever [in Japanese]. Shonika Rinsho (Japanese Journal of Pediatrics). 2017;(70):2148–53.

72. Miki S, Lee WC, Lee MJ. A Comparative Study of the Trends of Imported Dengue Cases in Korea and Japan 2011 - 2015. J Clin Med Res. 2017 Jul;9(7):650–3.

73. Miyashiro Y, Endo M, Kosugi S, Takara K, Nakahashi H, Kin Y, et al. Case Report: A case of severe dengue presenting with rhabdomyolysis [in Japanese]. Nihon Naika Gakkai Zasshi (The Journal of the Japanese Society of Internal Medicine). 2017;107(6):1090–4.

74. Miyata N, Yoshimura Y, Tachikawa N, Amano Y, Sakamoto Y, Kosuge Y. Cavity Forming Pneumonia Due to Staphylococcus aureus Following Dengue Fever. Am J Trop Med Hyg. 2015 Nov;93(5):1055–7.

75. Mizuno Y, Kato Y, Kano S, Takasaki T. Imported malaria and dengue fever in returned travelers in Japan from 2005 to 2010. Travel Med Infect Dis. 2012 Mar;10(2):86–91.

76. Moi ML. Dengue and chikungunya: Then & Now [in Japanese]. Jikken igaku. 2015;33(17):32–8.

77. Moi ML, Omatsu T, Tajima S, Lim CK, Kotaki A, Ikeda M, et al. Detection of dengue virus nonstructural protein 1 (NS1) by using ELISA as a useful laboratory diagnostic method for dengue virus infection of international travelers. J Travel Med. 2013 Jun;20(3):185–93.

78. Moi ML, Takasaki T, Kotaki A, Tajima S, Lim CK, Sakamoto M, et al. Importation of dengue virus type 3 to Japan from Tanzania and Cote d’Ivoire. Emerg Infect Dis. 2010 Nov;16(11):1770–2.

79. Morita K. Dengue Fever: A Tropical Infectious Disease Creeping into Japan [in Japanese]. Kansensyo Dojo. 2014 Sep;3(3):41–4.

80. Morita K. Dengue fever [in Japanese]. Seijinbyo to Seikatusyukanbyou. 2016;46(11):1389–93.

81. Morita K. Up-to-date imported infectious disease. Topics:V. Dengue fever:current situation and trends [in Japanese]. Nihon Naika Gakkai Zasshi (The Journal of the Japanese Society of Internal Medicine). 2016;105(11):2140–5.

82. Morita K. Afferent infection diseases/dengue [in Japanese]. Nihon Shounika Gakkai Zasshi (The journal of the Japan Pediatric Society). 2017;(53):60–4.

83. Morita K. Virus: Dengue fever [in Japanese]. Nihon Iji Shimpo (Japan Medical Journal). 2019;(4976):24–6.

84. Mutho Y, Moriya A, Yasui Y, Saito N, Takasaki T, HIramatsu S. Two Cases of Dengue Virus Type 2 (DENV-2) Infection in a Japanese Couple Returning from the Maldives during the 2018 Dengue Outbreak. Japanese Journal of Infectious Diseases. 2020;(73):58–60.

85. Nakamura N, Arima Y, Shimada T, Matsui T, Tada Y, Okabe N. Incidence of dengue virus infection among Japanese travellers, 2006 to 2010. Western Pac Surveill Response J. 2012 Apr;3(2):39–45.

86. Nakano K. Future risk of dengue fever to workforce and industry through global supply chain. Mitig Adapt Strateg Glob Chang. 2018;23(3):433–49.

87. Nakayama E, Kotaki A, Tajima S, Kawada M, Miura K, Gemma A, et al. Two different dengue virus strains in the Japanese epidemics of 2014. Virus Genes. 2016 Oct;52(5):722–6.

88. Nguyen Minh Tuan, Suzuki K. Dengue: An re-emerging infectious disease in the world [in Japanese]. Nippon Rinsho. 2021;79(2):235–47.

89. Ngwe Tun MM, Muta Y, Inoue S, Morita K. Persistence of Neutralizing Antibody Against Dengue Virus 2 After 70 Years from Infection in Nagasaki. Biores Open Access. 2016;5(1):188–91.

90. Ohmagari N. Intensive care for emerging infectious diseases-Ebola and Dellgue-[in Japanese]. Nippon Rinsho. 2016;74(2):252–6.

91. Ohnishi K. Needle-stick dengue virus infection in a health-care worker at a Japanese hospital. J Occup Health. 2015;57(5):482–3.

92. Ohnishi T, Murada T, Kishino Y, Homma T, Kusumoto Z, Yamamoto M. Two Japanese citizens infected with different types of dengue virus after travel to the Philippines[in Japanese]. Syowagakushikaishi. 2017;77(2):215–9.

93. Okada K, Morita R, Egawa K, Hirai Y, Kaida A, Shirano M, et al. Dengue Virus Type 1 Infection in Traveler Returning from Tanzania to Japan, 2019. Emerg Infect Dis. 2019 Sep;25(9):1782–4.

94. Oki M, Yamamoto T. Simulation of the probable vector density that caused the Nagasaki dengue outbreak vectored by *Aedes albopictus* in 1942. Epidemiol Infect. 2013 Dec;141(12):2612–22.

95. Oki Y, Kitazawa K, Honda A, Senda A, Kobayashi H, Arahata Y. Case of a 13-year-old girl with dengue fever who developed the disease after participating in an overseas training project. Nihon Toko igakkai (Journal of the Japanese Society of Travel and Health). 2017;11(2):89–92.

96. Onishi K. Dengue Virus Infection - Clinical Aspects - [in Japanese]. Shonika Rinsho (Japanese Journal of Pediatrics). 2015 Jan;68(1):13–8.

97. Oosako H. Classification and Ecology of Mosquito and Research of Mosquitoes of Japanese Encephalitis Virus in Kumamoto [in Japanese]. Jui Eikigaku Zasshi (Journal of Veterinary Epidemiology). 2015;19(1):4–6.

98. Quam MB, Sessions O, Kamaraj US, Rocklöv J, Wilder-Smith A. Dissecting Japan’s Dengue Outbreak in 2014. Am J Trop Med Hyg. 2016 Feb;94(2):409–12.

99. Ruiling Z, Tongkai L, Dezhen M, Zhong Z. Genetic characters of the globally spread tiger mosquito, Aedes albopictus (Diptera, Culicidae): implications from mitochondrial gene COI. J Vector Ecol. 2018 Jun;43(1):89–97.

100. Saijo M. Emerging and Re-emerging Virus Infections in Japan: Dengue Fever, Zika Virus Disease, and SFTS [in Japanese]. Yakugaku Zasshi. 2020;140(7):895–9.

101. Saito M, Tamayose M, Miyagi K, Takaragawa H, Tateyama M, Tadano M, et al. Serologic and Virologic Studies of an Imported Dengue Case Occurring in 2014 in Okinawa, Japan. Jpn J Infect Dis. 2016;69(1):60–5.

102. Saito N, Kitashouji E, Kojiro M, Furumoto A, Morimoto K, Morita K, et al. A Case of Clinically Mild Encephalitis!encephalopathy with a Reversible Splenial Lesion due to Dengue Fever [in Japanese]. Kansenshogaku Zasshi (The Journal of the Japanese Association for Infectious Diseases). 2015;89(4):465–9.

103. Sakamoto N. Domestic outbreak of dengue fever [in Japanese]. Nihon Naika Gakkai Zasshi (The Journal of the Japanese Society of Internal Medicine). 2014;103(11):2653–6.

104. Sakudo A, Onodera T, Shintani H, Ikuta K. Dengue virus presence and surveillance in Okinawa (Review). Exp Ther Med. 2012 Jan;3(1):15–7.

105. Sasaki T, Higa Y, Bertuso AG, Isawa H, Takasaki T, Minakawa N, et al. Susceptibility of Indigenous and Transplanted Mosquito Spp. to Dengue Virus in Japan. Jpn J Infect Dis. 2015;68(5):425–7.

106. Sato R, Hamada N, Kashiwagi T, Imamura Y, Hara K, Nishimura M, et al. Dengue Hemorrhagic Fever in a Japanese Traveler with Pre-existing Japanese Encephalitis Virus Antibody. Trop Med Health. 2015 Jun;43(2):85–8.

107. Sawabe K. Autochthonous dengue outbreak in Japan after a blank of 70 years and the future prediction of such cases [in Japanese]. Medical Entomology and Zoology. 2015;66(4):203–5.

108. Sawabe K. The 44th Scientific Meeting:Perspectives of Internal Medicine;Virology for physician~recent advance in viral infection and treatment~; 4. Viral disease commonly observed in outpatient clinic;2)Mosquito-borne infectious diseases, such as Dengue fever, and its vector mosquitoes [in Japanese]. Nihon Naika Gakkai Zasshi (The Journal of the Japanese Society of Internal Medicine). 2017;106(3):444–50.

109. Sawabe K. A series of the studies on countermeasure for arthropod-borne infectious diseases in Japan -Focus on highly pathogenic avian influenza and dengue outbreaks in Japan-[in Japanese]. Medical Entomology and Zoology. 2020;71(1):1–13.

110. Sawabe K, Isawa H, Hoshino K, Sasaki T, Roychoudhury S, Higa Y, et al. Host-feeding habits of Culex pipiens and Aedes albopictus (Diptera: Culicidae) collected at the urban and suburban residential areas of Japan. J Med Entomol. 2010 May;47(3):442–50.

111. Schmidt TL, Chung J, Honnen AC, Weeks AR, Hoffmann AA. Population genomics of two invasive mosquitoes (Aedes aegypti and Aedes albopictus) from the Indo-Pacific. PLoS Negl Trop Dis. 2020 Jul;14(7):e0008463.

112. Schmidt-Chanasit J, Emmerich P, Tappe D, Gunther S, Schmidt S, Wolff D, et al. Autochthonous dengue virus infection in Japan imported into Germany, September 2013. Euro Surveill. 2014 Jan 23;19(3).

113. Schwarz NG, Kreuels B, Stark K, Frank C, Schmidt-Chanasit J. Authors reply: diagnosis of a single imported dengue case who had travelled to Japan - how serious is it for travellers? Euro Surveill. 2014 Feb 27;19(8):20716.

114. Seki N, Iwashita Y, Moto R, Kamiya N, Kurita M, Tahara N, et al. An autochthonous outbreak of dengue type 1 in Tokyo, Japan 2014 [in Japanese]. Nihon Koshu Eisei Zasshi(JAPANESE JOURNAL OF PUBLIC HEALTH). 2015;62(5):238–50.

115. Senda A, Sakuntabhai A, Inaida S, Teissier Y, Matsuda F, Paul RE. Estimating Frequency of Probable Autochthonous Cases of Dengue, Japan. Emerg Infect Dis. 2018 Sep;24(9):1705–8.

116. Shimada T, Kanayama A, Matsui T, Kawabata K, Fukusumi M, Arima Y, et al. Epidemiology of autochthonous dengue virus infection in Japan in 2014 and preparing for future vector-borne disease outbreaks [in Japanese]. Medical Entomology and Zoology. 2016;67(1):39–41.

117. Shimizu H, Tsukiji jun. Emerging and re-emerging infectious diseases are landing in Japan in increasing numbers [in Japanese]. Emergency Care. 2015;28(12):10–6.

118. Shinohara K, Kutsuna S, Kato Y, Yamamoto K, Fujiya Y, Mawatari M. Retrospective Analysis of the Final Diagnosis of the Suspected Cases of Autochthonous Dengue Fever [in Japanse]. Kansenshogaku Zasshi (The Journal of the Japanese Association for Infectious Diseases). 2017;91(6):930–5.

119. Siengsanan-Lamont J, Blacksell SD. A Review of Laboratory-Acquired Infections in the Asia-Pacific: Understanding Risk and the Need for Improved Biosafety for Veterinary and Zoonotic Diseases. Trop Med Infect Dis. 2018 Mar 26;3(2).

120. Stoney RJ, Esposito DH, Kozarsky P, Hamer DH, Grobusch MP, Gkrania-Klotsas E, et al. Infectious diseases acquired by international travellers visiting the USA. J Travel Med. 2018 Aug 1;25(1).

121. Sugimoto M, Haseyama M, Ishida Y, Yoshida S, Kamiya K. Evaluation of nonstructural 1 protein rapid test for dengue virus at the Narita Airport quarantine station, Japan. Jpn J Infect Dis. 2011;64(2):169–70.

122. Sunahara T. Extremely High Biting Densities of Aedes albopictus (Skuse) (Diptera: Culicidae) at a University Campus in Nagasaki, Japan. Jpn J Infect Dis. 2019 Nov 21;72(6):368–73.

123. Suzuki T, Kutsuna S, Taniguchi S, Tajima S, Maeki T, Kato F, et al. Dengue Virus Exported from Côte d’Ivoire to Japan, June 2017. Emerg Infect Dis. 2017 Oct;23(10):1758–60.

124. Tada Y, Umemura S, Higure H, Hamada A. Survey of Japanese citizens on dengue fever preparedness and knowledge level [in Japanese]. Nihon Toko igakkai ( Journal of the Japanese Society of Travel and Health). 2015;9(1):16–9.

125. Tajima S, Nakayama E, Kotaki A, Moi ML, Ikeda M, Yagasaki K, et al. Whole Genome Sequencing-Based Molecular Epidemiologic Analysis of Autochthonous Dengue Virus Type 1 Strains Circulating in Japan in 2014. Jpn J Infect Dis. 2017 Jan 24;70(1):45–9.

126. Tajima S, Takasaki T. Dengue fever [in Japanese]. Nippon Rinsho. 2016;74(12):2042–6.

127. Takamatsu Y, Morita K. Dengue vaccine [in Japanese]. Rinsyo to Biseibutsu. 2014;41(6):75–82.

128. Takasaki T. Imported dengue fever/dengue hemorrhagic fever cases in Japan. Trop Med Health. 2011 Dec;39(4 Suppl):13–5.

129. Takasaki T. Domestic dengue epidemic in Japan, 2014 [in Japanese]. Virus. 2014;65(1):115–8.

130. Takasaki T. Yellow fever and Dengue fever [in Japanese]. BMSA journal (The Journal of Biomedical Science and Biosafety). 2014;26(3):26–9.

131. Takasaki T. After 70 Years : The Return of Dengue Fever [in Japanese]. Nihon Iji Shimpo (Japan Medical Journal). 2014 Oct;(4719):51–51.

132. Takasaki T. Dengue fever: Virus and vector mosquitoes [in Japanese]. Shonika Rinsho (Japanese Journal of Pediatrics). 2015 Jan;68(1):7–12.

133. Takasaki T. Dengue fever [in Japanese]. Igaku No Ayumi (Journal of Clinical and Experimental Medicine). 2015 Apr;254(1):69–74.

134. Takasaki To. Mosquito-borne Infectious Disease Control. Medicament News. 2016;(2232).

135. Takasaki T. Will dengue fever continue to be endemic in the country? [in Japanese]. Child Health. 2019;22(4):21–4.

136. Tanikawa T, Watanabe T, Motoki M, Shimizu I, Kasai S. Insecticide spray training at Shinjuku Gyoen National Garden in preparation for autochthonous dengue fever [in Japanese]. Medical Entomology and Zoology. 2020;71(2):79–83.

137. TANIKAWA T, YAMAUCHI M, ISHIHARA S, TOMIOKA Y, KIMURA G, TANAKA K, et al. Operation note on dengue vector control against Aedes albopictus in Chiba City, Japan, where an autochthonous dengue case was confirmed in September 2014. Medical entomology and zoology. 2015 May 23;66:31–3.

138. Tarumoto N, Abe Y, Yamaguchi T, Takasaki T, Kurane I, Maesaki S. Dengue fever as an acute febrile disease after overseas travel: a report of two cases. Jpn J Infect Dis. 2011;64(2):163–4.

139. Tatsumi Y, Murano T. Awareness Education on Emergency Response and Dengue Prevention: Experiences of Videoconferencing in Kuala Lumpur [in Japanese]. Nihon Toko igakkai ( Journal of the Japanese Society of Travel and Health). 2018;12(2):87–9.

140. Tsuboi M, Kutsuna S, Maeki T, Taniguchi S, Tajima S, Kato F, et al. Dengue Virus Type 2 in Travelers Returning to Japan from Sri Lanka, 2017. Emerg Infect Dis. 2017 Nov;23(11):1931–3.

141. Tsuda Y. Evaluation of risk of human infection with dengue virus based on entomological surveys conducted during the dengue outbreak in Yoyogi area, Tokyo 2014 [in Japanese]. Medical Entomology and Zoology. 2015;66(4):211–7.

142. Tsuda Y, Maekawa Y, Ogawa K, Itokawa K, Komagata O, Sasaki T, et al. Biting Density and Distribution of Aedes albopictus during the September 2014 Outbreak of Dengue Fever in Yoyogi Park and the Vicinity of Tokyo Metropolis, Japan. Jpn J Infect Dis. 2016;69(1):1–5.

143. Ueno R, Nishiura H. Letter to the editor: diagnosis of a single imported dengue case who had traveled to Japan - how serious is it for travelers? Euro Surveill. 2014 Feb 27;19(8):20715.

144. Ujiie M, Moi ML, Kobayashi T, Takeshita N, Kato Y, Takasaki T, et al. Dengue virus type-3 infection in a traveler returning from Benin to Japan. J Travel Med. 2012 Jul;19(4):255–7.

145. Watanabe H. The circumstance of dengue vaccine [in Japanese]. Nihon Naika Gakkai Zasshi (The Journal of the Japanese Society of Internal Medicine). 2019;108(5):962–3.

146. Yaita K, Kurashige T, Katou M, Takahama S, Hara Y. A case of domestic hepatitis A infection referred as suspected dengue fever after travel to Japan. Nihon Toko igakkai ( Journal of the Japanese Society of Travel and Health). 2019;12(2):77–80.

147. Yaita K, Sakai Y, Iwahashi J, Masunaga K, Hamada N, Watanabe H. Post-Travel Consultations in a Regional Hub City Hospital, Japan. Intern Med. 2016;55(7):739–43.

148. Yamamoto SP, Kasamatsu Y, Kanbayashi D, Kaida A, Shirano M, Kubo H, et al. Dengue Virus in Traveler Returning to Japan from the Democratic Republic of the Congo, 2015. Jpn J Infect Dis. 2019 Nov 21;72(6):426–8.

149. Yamashita A, Sakamoto T, Sekizuka T, Kato K, Takasaki T, Kuroda M. DGV: Dengue Genographic Viewer. Front Microbiol. 2016;7:875.

150. Yanagisawa N, Wada K, Spengler JD, Sanchez-Pina R. Health preparedness plan for dengue detection during the 2020 summer Olympic and Paralympic games in Tokyo. PLoS Negl Trop Dis. 2018 Sep;12(9):e0006755.

151. Yano K. Dengue fever [in Japanese]. CDC guideline news. 2014;23(5):9.

152. Yoshida S, Fukasawa youhei, Masuda T, Sugatani M, Katori S, Tani T, et al. A case of dengue fever [in Japanese]. Shonika Rinsho (Japanese Journal of Pediatrics). 2013 Mar;66(3):465–8.

153. Yoshikawa M, Higure H, Hamada A. Survey of Japanese Residents in Singapore on Dengue Fever [in Japanese]. Nihon Toko igakkai ( Journal of the Japanese Society of Travel and Health). 2015;9(1):20–5.

154. Yoshikawa M, Miyake M. Survey of Japanese residents in Singapore on sources of information on infectious disease outbreaks and prevention of dengue fever [in Japanese]. Nihon Toko igakkai ( Journal of the Japanese Society of Travel and Health). 2017;11(1):15–21.

155. Yoshimura Y, Sakamoto Y, Amano Y, Nakaharai K, Yaita K, Hoshina T, et al. Four Cases of Autochthonous Dengue Infection in Japan and 46 Imported Cases: Characteristics of Japanese Dengue. Intern Med. 2015;54(23):3005–8.

156. Yuan B, Lee H, Nishiura H. Assessing dengue control in Tokyo, 2014. PLoS Negl Trop Dis. 2019 Jun;13(6):e0007468.

157. Yuan B, Nishiura H. Estimating the actual importation risk of dengue virus infection among Japanese travelers. PLoS One. 2018;13(6):e0198734.

*Chikungunya (47)*

1. Akanuma M. Chikunguya fever [in Japanese]. Nihonikadaigakuigakkai. 2012;8(1):58–58.

2. Chang Kweng Lim. Chikungunya virus expanding its endemic area [in Japanese]. Rinsho to Virus. 2016 Mar;44(1):29–36.

3. Eimoto A, Nakata T, Aoki M, Oiwa T, Adachi M, Takeuchi M. A case of Chikungunya fever after traveling to India [in Japanese]. Vol. 13, Nihon Kyukyu Igakukai Chubu Chihokaishi (Chuubu Journal of Acute Medicine). 2017. p. 22–4.

4. Eshita Y, Raweewan Srisawat, Lucky Ronald Runtuwene, Hidano S, Hayashida K, Orba Y. Mosquito-borne viruses [in Japanese]. Rinsho To Biseibutsu (Clinical Microbiology). 2016 Nov;43(6):701–7.

5. Fukushima shinji, Hamada A. Mosquito-borne infection [in Japanese]. Shonika Shinryo (The Journal of Pediatric Practice). 2019 Jun;82(6):775–81.

6. Fukushima S, Mizuno Y. Infectious Diseases Increasing with Internatinalization: Trends in Infectious Diseases of Foreign Origin in Japan - Focusing on Diseases Relevant to Children [in Japanese]. 2016 Apr;19(4):238–42.

7. Furuichi M, Makie T, Honma Y, Isoda T, Miyake S. Laboratory-Confirmed Dengue Fever and Chikungunya Fever Cases at the Narita Airport Quarantine Station in 2013. Jpn J Infect Dis. 2015;68(2):142–4.

8. Furuya H. Estimating Vector-borne Viral Infections in the Urban Setting of the 2020 Tokyo Olympics, Japan, Using Mathematical Modeling. 2020;5.

9. Hayano M, Endo H. Infectious Diseases (1) Emerging Travel-related Infections [in Japanese]. Tokyojoshiikadaigakuzasshi (Tokyo Women’s Medical University Journal). 2016;86(1):1–8.

10. Imai K, Nakayama E, Maeda T, Mikita K, Kobayashi Y, Mitarai A, et al. Chikungunya Fever in Japan Imported from the Caribbean Islands. Jpn J Infect Dis. 2016;69(2):151–3.

11. Isoda T, Shimada T. Current status of imported infectious diseases detected in quarantine [in Japanese]. Shonika Rinsho (Japanese Journal of Pediatrics). 2017;70:2109–16.

12. Kasai S, Ugajin T, Shimizu I, Muto A, Sasaki H, Ikeda F, et al. An emergency drill for controlling dengue mosquito vector toward Tokyo 2020 Olympic and Paralympic games [in Japanese]. Med Entomol Zool. 2020;71(2):67–71.

13. Kasai S, Ugajin T, Shimizu I, Muto A, Sasaki H, Ikeda F, et al. Outline of an emergency drill for controlling the mosquito vector of dengue fever held at Shinjuku Gyoen National Garden in 2019. Med Entomol Zool. 2020 Jun 25;71(2):67–71.

14. Kato Y. Mosquito-borne infection [in Japanese]. Yakkyoku. 2018 Jun;69(7):2532–6.

15. Katsuda Y. Survey of religious leaders’ awareness of countermeasures against mosquitoes carrying dengue fever, chikungunya fever [in Japanese]. Nihon Iji Shimpo (Japan Medical Journal). 2015 Aug;(4764):44–9.

16. Kobashi K, Kobayashi T, Nakamura-Uchiyama F, Onishi K. A Japanese Patient with Chikungunya Fever Returning from Flores Island, Indonesia [in Japanese]. Kansenshogaku Zasshi (The Journal of the Japanese Association for Infectious Diseases). 2010;84(4):457–9.

17. Kobayashi K, Onishi K. Nortable imported infectious diseases. Nihonijishinpo (Japan Medical Journal). 2013;(4664):27–32.

18. Kobayashi M. Mosquito-borne Infectious Diseases: Focus on Dengue Fever [in Japanese]. Dokkyo Journal of Medical Sciences. 2015;42(3):179–85.

19. Kondo M, Akachi S, Ando K, Nomura T, Yamanaka K, Mizutani H. Two Japanese siblings affected with Chikungunya fever with different clinical courses: Imported infections from the Cook Islands. J Dermatol. 2016 Jun;43(6):697–700.

20. Kurane I. Arbovirus diseases [in Japanse]. Rinsyo to Biseibutsu. 2012 Sep;39(5):456–70.

21. Kurimura T. Chikungunya Fever and its surroundings [in Japanse]. Rinsyo to Biseibutsu. 2011 Jan;38(1):78–78.

22. Kutsuna S. Chikungunya fever [in Japanese]. Shonika Rinsho (Japanese Journal of Pediatrics). 2017;70:2154–8.

23. Kutsuna S. Differences in the Clinical Presentation of Mosquito-borne Infections [in Japanese]. Rinsho to Virus. 2017;44(5):221–6.

24. Kutsuna S. Current Situation and Control Measure of Mosquito–borne Diseases [in Japanese]. Saishinigaku. 2017 Apr;72(4):541–5.

25. Kutsuna S. Emerging and Re-emerging Infectious Diseases [in Japanese]. Nihon Naikagakugakkaizasshi. 2018;107(11):2276–81.

26. Kutsuna S, Kato Y, Katanami Y, Yamamoto K, Takeshita N, Hayakawa K, et al. A Retrospective Single-center Analysis of 16 Cases of Imported Chikungunya Fever in Japan. Intern Med. 2018;57(3):325–8.

27. Liu Y, Lillepold K, Semenza JC, Tozan Y, Quam MBM, Rocklöv J. Reviewing estimates of the basic reproduction number for dengue, Zika and chikungunya across global climate zones. Environmental Research. 2020 Mar;182:109114.

28. Meng Ling Moi. Dengue and chikunguya: Then & Now [in Japanese]. Jikken igaku. 2015;33(17):2720–6.

29. Meng Ling Moi, Takasaki T. Recent advances in arbovirus diagnostics [in Japanese]. BMSA journal (The Journal of Biomedical Science and Biosafety). 2016;28(3):104–9.

30. Mishima N. Children’s Travel Medicine: Notes for children after living abroad and returning to Japan [in Japanese]. Child Health. 2018 May;21(5):380–3.

31. Miyamoto T. Infection Control for the Tokyo 2020 Olympic and Paralyrnpic Games [in Japanese]. BMSA journal (The Journal of Biomedical Science and Biosafety). 2020;32(1):24–8.

32. Mizuno Y, Kato Y, Takeshita N, Ujiie M, Kobayashi T, Kanagawa S, et al. Clinical and radiological features of imported chikungunya fever in Japan: a study of six cases at the National Center for Global Health and Medicine. Journal of Infection and Chemotherapy. 2011;17(3):419–23.

33. Mori Y, Otsuki N, Sakata M, Okamoto K. Virology of the family Togaviridae [in Japanese]. Uirusu. 2011;61(2):211–9.

34. Nabeshima T, Morita K. How do mosquito-borne viruses spread around the world? [in Japanese]. Rinsho to Virus. 2017 Jan;44(5):209–15.

35. Nakatani I, Kuroda T, Tabuchi K. An imported case of chikungunya fever with prolonged arthralgia [in Japanese]. 2012;6(1):45–7.

36. Nakatsubo N. Infectious disease prevention measures by the Tokyo Metropolitan Government [in Japanese]. Rinsho to Virus. 2019 Dec;47(5):409–14.

37. Nakayama E, Tajima S, Kotaki A, Shibasaki K, Itokawa K, Kato K, et al. A summary of the imported cases of Chikungunya fever in Japan from 2006 to June 2016. Journal of Travel Medicine [Internet]. 2018 Jan 1 [cited 2021 Feb 25];25(1). Available from: https://academic.oup.com/jtm/article/doi/10.1093/jtm/tax072/4763690

38. Nakayama E, Takasaki T. Chikungunya fever [in Japanese]. Shonika Shinryo (The Journal of Pediatric Practice). 2018 Apr;81(4):449–54.

39. Ohnishi K. Notable imported infectious diseases [in Japanese]. Kansenshogaku zasshi The Journal of the Japanese Association for Infectious Diseases. 2011;85(2):139–43.

40. Sasaki Y, Manda S, Sato T, Maeda T, Miyazaki T, Nakanishi K, et al. Chikungunya Virus Infection Presenting with Persistent Arthralgia without Fever. Journal of General and Family Medicine. 2015 Oct;16(3):204–7.

41. Takasaki T. Dengue fever: After 70 years [in Japanese]. Rinsho to Virus. 2015 Oct;43(4):194–7.

42. Takaya S, Kutsuna S, Nakayama E, Taniguchi S, Tajima S, Katanami Y, et al. Chikungunya Fever in Traveler from Angola to Japan, 2016. Emerg Infect Dis. 2017 Jan;23(1):156–8.

43. Tsuboi M, Kutsuna S, Kato Y, Nakayama E, Shibasaki K ichi, Tajima S, et al. Autochthonous Chikungunya Fever in Traveler Returning to Japan from Cuba. Emerg Infect Dis. 2016 Sep;22(9):1683–5.

44. Tuboi M, Kutsuna S. Recent Topics on Mosquito-borne Infectious Diseases Chikungunya fever [in Japanese]. Rinsyo to Biseibutsu. 2017 May;44(3):253–6.

45. Yamamoto K, Matumoto K, Lim CK, Moi ML, Kotaki A, Takasaki T. Chikungunya Fever from Malaysia. Intern Med. 2010;49(5):501–5.

46. Yoshikawa MJ, Kusriastuti R. Surge of Dengue Virus Infection and Chikungunya Fever in Bali in 2010: The Burden of Mosquito-Borne Infectious Diseases in a Tourist Destination. TropMedHealth. 2013;41(2):67–78.

47. Yoshikawa MJ, Tang CS, Nishibuchi M. Incidence of Chikungunya Fever in Singapore: Implications of Public Health Measures and Transnational Movements of People. TropMedHealth. 2010;38(1):39–45.

**Dracunculiasis (0)**

**Echinococcosis (74)**

1. Adachi Y, Makita K. Time series analysis based on two-part models for excessive zero count data to detect farm-level outbreaks of swine echinococcosis during meat inspections. Prev Vet Med. 2017;148:49–57.

2. Angoin T. Studies of echinococcosis and animal experiments. LABIO 21. 2019. p. 18–21.

3. Asano K. Becoming a zoonosis detective Ayaka Imai - Yuto going around the north - echinococcosis. Vol. 26, as: animal specialist. 2014. p. 70–6.

4. Chida K, Kamiyama T, Kakisaka T, Yokoo H, Hatanaka K, Taketomi A. A Case of Hepatic Alveolar Echinococcosis with Complete Obstruction of the Inferior Vena Cava. Nihon Rinsho Geka Gakkai Zasshi (Journal of Japan Surgical Association). 2015;76(5):1124–9.

5. Fukumoto S. Immunological diagnosis of infectious diseases - non-viral infections - echinococcosis. Vol. 68, Nihon Rinsyo (Japanese Journal of Clinical Medicine). 2010. p. 292–5.

6. Fukumoto S, Yamada S, Fushikida M, Toyoda S, Nishikawa T, Higuchi H, et al. Natural larval Echinococcus multilocularis infection in a Norway rat, Rattus norvegicus, captured indoors in Hokkaido, Japan. Vol. 79, The Journal of Veterinary Medical Science. 2017. p. 1857–60.

7. Goto Y, Sato K, Yahagi K, Komatsu O, Hoshina H, Abiko C, et al. Frequent isolation of Echinococcus multilocularis from the livers of racehorses slaughtered in Yamagata, Japan. Jpn J Infect Dis. 2010;63(6):449–51.

8. Guo ZH, Kubo M, Kudo M, Nibe K, Horii Y, Nonaka N. Growth and genotypes of Echinococcus granulosus found in cattle imported from Australia and fattened in Japan. Vol. 60, Parasitology International. 2011. p. 498–502.

9. Hata R, Toyama M, Ichimura Y, Sawada Y, Kaneko M. A case of echinococcosis of the liver involving parent and child. Japanese Journal of Medical Technology. 2014;63(1):69–73.

10. Hifumi T, Ikeda K, Etoh Y, Ikawa K, Nishimura K, Ogawa T, et al. The Relationship between Hepatic Grayish White Solid Nodules Found in Horses Brought to a Slaughterhouse in Fukuoka Prefecture and Larval Echinococcus multilocularis Infection. Journal of the Japan Veterinary Medical Association. 2015;68(4):253–7.

11. Hisai H, Miyazaki E, Tanaka I, Okuda T, Kikuchi S. Parasitic diseases in the liver - hepatic alveolar echinococcosis. Vol. Suppl, Nihon Rinsyo (Japanese Journal of Clinical Medicine). 2010. p. 103–9.

12. Horiguchi I, Ishikawa N, Feng Q, Kigawa M, Marui E. Which Zoonoses should the General Population be More Awareness of? -Qualitative Research Targeted at Veterinarians-. Vol. 66, Nihon Eiseigaku Zasshi (Japanese Journal of Hygiene). 2011. p. 741–5.

13. Ikeda T, Yoshimura M, Onoyama K, Oku Y, Nonaka N, Katakura K. Where to deliver baits for deworming urban red foxes for Echinococcus multilocularis control: new protocol for micro-habitat modeling of fox denning requirements. Parasit Vectors. 2014;7:357.

14. Imai T. Infectious and parasitic diseases - teniasis. Vol. Suppl, Nihon Rinsho (Japanese Journal of Clinical Medicine). 2013. p. 912–6.

15. Irie T, Mukai T, Yagi K. Echinococcus multilocularis Surveillance Using Copro-DNA and Egg Examination of Shelter Dogs from an Endemic Area in Hokkaido, Japan. Vector Borne Zoonotic Dis. 2018;18(7):390–2.

16. Irie T, Yamada K, Morishima Y, Yagi K. High probability of pet dogs encountering the sylvatic cycle of Echinococcus multilocularis in a rural area in Hokkaido, Japan. Vol. 81, The Journal of Veterinary Medical Science. 2019. p. 1606–8.

17. Ishida T. Zoonosis (2). Vol. 27, CAP: Companion Animal Practice. 2012. p. 60–5.

18. Ishida T. JBVP Lecture Series - infectious diseases of dogs and cats. Vol. 31, CAP: Companion Animal Practice. 2016. p. 66–74.

19. Isogai T. Future of One Health around the control of zoonotic diseases. Vol. 71, Nihon Zyuishikai Zasshi (Journal of the Japan Veterinary Medical Association). 2018. p. 276–7.

20. Ito R, Sako Y, Ishikawa Y. Protozoan diseases, parasitic diseases, taeniasis, and echinococcosis. Vol. Suppl, Nihon Rinsyo (Japanese Journal of Clinical Medicine). 2013. p. 723–7.

21. Ito T. Echinococcosis. In: Byori to Rinsho (Pathology and Clinical Medicine) [Internet]. Bunkodo; 2018 [cited 2021 Jan 15]. p. 281–5. Available from: http://mol.medicalonline.jp/library/journal/abstract?GoodsID=ag9brysh/2018/0036s1/051&name=0281-0285j&UserID=1100001448-00

22. Kataoka R, Kojima K. A case of alveolar echinococcosis of the liver presented without associated history. Vol. 62, Rinsho Houshasen (Japanese Journal of Clinical Radiology). 2017. p. 571–4.

23. Kawamoto T, Yoshida K. Pericardial diseases - protozoa and parasitic pericarditis. Vol. Suppl, Nihon Rinsho (Japanese Journal of Clinical Medicine). 2019. p. 523–6.

24. Kimura M, Toukairin A, Tatezaki H, Tanaka S, Harada K, Araiyama J, et al. Echinococcus multilocularis Detected in Slaughtered Pigs in Aomori, the Northernmost Prefecture of Mainland Japan. Vol. 63, Japanese Journal of Infectious Diseases. 2010. p. 80–1.

25. Kuchikata T. A case of 13-year-old girl with look-alike hepatophyma. Vol. 77, Shonika Shinryo (The Journal of Pediatric Practice). 2014. p. 553–6.

26. Kurai H. Record of endemic diseases and parasitic infections - tumor mass of the liver. Vol. 28, Infection Control. 2019. p. 1144–5.

27. Maru S, Shinohara N. A case of suspected echinococcosis with the history of residing in Hokkaido. Vol. 69, Rinsho Hinyokika (Japanese Journal of Clinical Urology). 2015. p. 394–7.

28. Matsudaira S, Ishizaki Y, Yoshimoto J, Imamura H, Fukumura Y, Kawasaki S. A Case of Hepatic Alveolar Echinococcosis Treated Radically by Hepatectomy 29 Years after Infection by Echinococcus multilocularis. Nihon Rinsho Geka Gakkai Zasshi (Journal of Japan Surgical Association). 2018;79(10):2145–9.

29. Matsumoto J. One Health and parasites: echinococcosis. Vol. 59, Twig’s. 2013. p. 1–10.

30. Matsuyama N, Serizawa K, Oshiro Y, Kamitani M. Fungal and parasitic infections of the lung. Vol. 32, Rinsyo Gazo (Clinical Imagiology. 2016. p. 60–9.

31. Mishima N, Lamaningao P, Nishiyama T. How much are Japanese infected with parasitic diseases and what kind of? Vol. 27, Medical Practice. 2010. p. 1444–50.

32. Miura S. Recent topics on parasitic diseases spreading over the world. Vol. 43, Tokyoto Igaku Kensa (Tokyo Metropolitan of Medical Technology). 2015. p. 289–93.

33. Morishima Y. Current topics on zoonosis: echinococcosis. Vol. 71, Nihon Zyuishikai Zasshi (Journal of the Japan Veterinary Medical Association). 2018. p. 333–7.

34. Morishima Y, Ichimura S, Yamazaki H, Sugiyama H. Cystic echinococcosis in a Napalese person - infection of heart with Echinococcus ortleppi. Vol. 25, Clinical Parasitology. 2014. p. 99–101.

35. Morishima Y, Tomaru Y, Fukumoto S ichiro, Sugiyama H, Yamasaki H, Hashimoto C, et al. Canine Echinococcosis Due to Echinococcus multilocularis: a Second Notifiable Case from Mainland Japan. Jpn J Infect Dis. 2016;69(5):448–9.

36. Morishima Y, Yamazaki H, Omae H, Sugiyama H. Cystic echinococcosis in Japan - the current situation and accuracy of commercially available diagnostic tools. Vol. 27, Clinical Parasitology. 2016. p. 69–71.

37. Nakamuma Y, Sato Y. Hepatic granuloma and parasitic diseases of the hepatobiliary system. Vol. Suppl, Nihon Rinsyo (Japanese Journal of Clinical Medicine). 2010. p. 348–53.

38. Nakamura F. Key infectious diseases - helminthiases. Vol. 143, Nihon Ishikai Zashi (The Journal of the Japan Medical Association). 2014. p. S410–4.

39. Nakamura F. Respiratory infections and pathogens - lung parasitic diseases. Vol. 73, Nihon Kyobu Rinsho (The Japanese Journal of Chest Diseases). 2014. p. S82–9.

40. Nakamura F, Onishi K. Parasitic diseases that have a small number of infected people but cannot be overlooked - cases of parasitic infections in foreign countries. Vol. 43, Kansensyo (The Infection). 2013. p. 33-39,29-32.

41. Nakamura K, Ito A, Yara S, Haranaga S, Hibiya K, Hirayasu T, et al. A case of pulmonary and hepatic cystic Echinococcosis of CE1 stage in a healthy Japanese female that was suspected to have been acquired during her stay in the United Kingdom. Am J Trop Med Hyg. 2011 Sep;85(3):456–9.

42. Nishiura H, Oishi K, Yoshii K, Uraguchi K. Current topics on infectious diseases. Vol. 93, Hokkaido Igaku Zasshi (The Hokkaido Journal of Medical Science). 2018. p. 5–12.

43. Ochiai A. Diagnostics for animals that are available in Japan - overview of infectious diseases in dogs and their diagnostics - Canine echinococcosis. Vol. 66, Nihon Zyuishikai Zasshi (Journal of the Japan Veterinary Medical Association). 2013. p. 663–4.

44. Oikawa E, Shimura R, Nishimura M, Furuoka H. First Case of Echinococcus multilocularis Infection in a Zoo-Housed Flying Squirrel (Pteromys volans orii). Vol. 75, The Journal of Veterinary Medical Science. 2013. p. 659–61.

45. Ojima H, Kanai Y. Alveolar echinococcosis - hint for diagnosis. In: Byori To Rinsho (Pathology and Clinical Medicine) [Internet]. Bunkodo; 2019 [cited 2021 Jan 15]. p. 203–5. Available from: http://mol.medicalonline.jp/library/journal/abstract?GoodsID=ag9brysh/2019/0037s1/058&name=0203-0205j&UserID=1100001448-00

46. Okada F, Kumai Y, Sato H, Ohbayashi H, Ono A, Ando Y, et al. Image diagnosis of infectious diseases - image findings of parasitic diseases in lung. Vol. 73, Nihon Kyobu Rinsho (The Japanese Journal of Chest Diseases). 2014. p. 1451–9.

47. Oku Y. Diagnosis of final hosts of Echinococcus multilocularis and control of sources of infections. Vol. 11, Zyui Kiseichu Kaishi (Jpn. J. Vet. Parasitol.). 2012. p. 8–14.

48. Oku Y. Echinococcosis. Vol. 33, Kagaku Ryoho no Ryoiki (Antibiotics & Chemotherapy). 2017. p. 428–35.

49. Omae H. Clinical pathology of parastics - imported parasitics such as malaria and zoonosis. Vol. 65, Nihon Zyuishikai Zasshi (Journal of the Japan Veterinary Medical Association). 2012. p. 829–33.

50. Onishi K. Review and update on treatment of echinoccosis. Nihon Iji Shimpo (Japan Medical Journal). 2020. p. 42.

51. Osawa E, Tsuchida T, Numada K, Yoneyama K, Kasahara A, Murakami A, et al. A case of alveolar echinococcosis of the liver in a resident of Kanagawa Prefecture which was difficult to diagnose. Vol. 63, Yokohama Igaku (Yokohama Medical Journal). 2012. p. 605–9.

52. Ota M, Kobayashi K, Washino T, Hikone M, Sakamoto N, et al. Two cases of hepatic multilocular cystic tumors with calcification. Vol. 26, Clinical Parasitology. 2015. p. 46–8.

53. Ota N, Ito R, Kita K. Prevention, diagnosis and treatment of emerging and re-emerging infectious diseases - parasitic infections (schistosomiasis and echinococcosis). Vol. 74, Nihon Rinsho (Japanese Journal of Clinical Medicine). 2016. p. 2036–41.

54. Saeki E. Parasitic diseases that should be recognized by clinical veterinarians: the relationship between human beings, animals, and echinococcosis. Why is the infection of dogs problematic? Vol. 10, Clinic Note. 2014. p. 87–96.

55. Sakui M, Yuuki M, Oonishi A, Nakano Y, Toyooka D, et al. Alveolar echinococcosis in the horse showing multilocular vesiculation in the liver. Vol. 14, Zyui Kiseichu Kaishi (Jpn. J. Vet. Parasitol.). 2015. p. 76–82.

56. Shibayama K. Antibody detection of special pathogens. Vol. 1, Kansensyo Naika (Infectious Diseases Medicine). 2013. p. 31–4.

57. Shimizu S. Parasitic infections from the perspective of infectious disease doctors. Vol. 86, Zin to Touseki (Kidney and Dialysis). 2019. p. 406–9.

58. Sunahara M, Kamiyama T, Sato N, Kurauchi N, Suzuki S, Kimura J. A Case of Hepatic Alveolar Echinococcosis with Peritoneal Dissemination. Nihon Rinsho Geka Gakkai Zasshi (Journal of Japan Surgical Association). 2013;74(10):2857–62.

59. Suzuki Y, Takago Y. Updates on diagnosis of multilocular hepatic echinococcosis. Vol. 141, Nihon Ishikai Zashi (The Journal of the Japan Medical Association). 2012. p. S279.

60. Tada Y, Sato H, Takayama N, Okabe N. Current situation of zoonosis by Infectious Disease Weekly Reports. Vol. 64, Zyui Chikusan Shinpo (Journal of Veterinary Medicine). 2011. p. 295–302.

61. Takahashi K, Uraguchi K, Hatakeyama H, Giraudoux P, Romig T. Efficacy of anthelmintic baiting of foxes against Echinococcus multilocularis in northern Japan. Vet Parasitol. 2013 Nov 15;198(1–2):122–6.

62. Takahashi K. Pathologies of kidney disorders - parasitic infections in the kidney. Vol. Suppl, Nihon Rinsho (Japanese Journal of Clinical Medicine). 2012. p. 683–7.

63. Takayama Y. A case of fever of unknown origin in a patient from Okinawa. Vol. 6, Gekkan Resident (Resident). 2013. p. 89–95.

64. Tanaka T, Hirata T, Aragaki S, Higashiarakawa M, Kishimoto K, et al. A case of cystic echinococcosis in an international student from Napal. Vol. 25, Clinical Parasitology. 2014. p. 95–8.

65. Vuitton DA, Wang Q, Zhou HX, Raoul F, Knapp J, Bresson-Hadni S, et al. A historical view of alveolar echinococcosis, 160 years after the discovery of the first case in humans: part 1. What have we learnt on the distribution of the disease and on its parasitic agent? Chin Med J (Engl). 2011 Sep;124(18):2943–53.

66. Yagi K. Frontline of global infectious diseases - beyond NTDs - echinococcosis - current situation of alveolar echinococcosis in Japan and its challenges as a zoonosis. Vol. 259, Igaku No Ayumi (Journal of Clinical and Experimental Medicine). 2016. p. 881–7.

67. Yagi K. The Control Measures of Alveolar Echinococcosis Conducted by Hokkaido Local Government. Report of the Hokkaido Institute of Public Health. 2017. p. 1–7.

68. Yagi K. Zoonosis - echinococcosis. Vol. 70, Shonika Rinsho (Japanese Journal of Pediatrics). 2017. p. 2415–22.

69. Yagi K, Uraguchi K, Sakui M. Epidemiological importance of detecting porcine echinococcosis. Vol. 13, Zyui Kiseichu Kaishi (Jpn. J. Vet. Parasitol.). 2014. p. 46–53.

70. Yamano K, Kouguchi H, Uraguchi K, Mukai T, Shibata C, Yamamoto H, et al. First detection of Echinococcus multilocularis infection in two species of nonhuman primates raised in a zoo: a fatal case in Cercopithecus diana and a strongly suspected case of spontaneous recovery in Macaca nigra. Parasitol Int. 2014 Aug;63(4):621–6.

71. Yamashita N, Hirai T, Ito T. A case of liver cystic echinococcosis. Journal of Gastroenterological Cancer Screening. 2010;48(1):61–6.

72. Yasunaga M, Kinoshita T. Cystic diseases in the liver - parasitic liver cysts. Vol. Supp;, Nihon Rinsho (Japanese Journal of Clinical Medicine). 2010. p. 393–7.

73. Yoshida T, Kamiyama T, Okada T, Nakanishi K, Yokoo H, Kamachi H, et al. Alveolar echinococcosis of the liver in children. Vol. 17, Journal of Hepato-Biliary-Pancreatic Sciences. 2010. p. 152–7.

74. Yoshikawa M, Sawada A, Mase F, Hirai N, Onishi T, et al. Control and prevention of pediatric parasitic diseases. Vol. 59, Shonika (Pediatrics of Japan). 2018. p. 1079–86.

**Foodborne trematodiases (74)**

*Clonorchiasis (3)*

1. Kominami Y, Aikata H, Hiramatsu K, Tanaka M, Naeshiro N. A case of clonorchiasis complicated with the expansion of liver cyst. Journal of Japanese Society of Gastroenterology. 2013;110(3):456–64.

2. Matsubayashi J, Taira K, Yogo A, Kito Y, Ura K, Toyota E, et al. Intrahepatic Bile Duct Carcinoma with Clonorchiasis. Jpn J Gastroenterol Surg. 2015;48(4):328–36.

3. Yoshida Y. Clonorchiasis--a historical review of contributions of Japanese parasitologists. Parasitol Int. 2012 Mar;61(1):5–9.

*Paragonimiasis (59)*

1. Akaba T, Takeyama K, Toriyama M, Kubo A, Mizobuchi R, Yamada T, et al. Pulmonary Paragonimiasis: The Detection of a Worm Migration Track as a Diagnostic Clue for Uncertain Eosinophilic Pleural Effusion. Internal Medicine. 2016;55(5):503–6.

2. Arai A, Tadokoro A, Kaneji T, Ishii T, Takagi T, Watanabe N, et al. A Case of Mixed Infection with *Paragonimus westermani* and *Opisthorchis viverrini* in a Laotian Resident in Japan (Japanese). Nihon Rinsho Kiseichu Gakkai shi (Clinical Parasitology). 2016;27(1):32–5.

3. Banzai A, Sasaki T, Sugiyama H, Kawakami Y. Infection status of *Paragonimus westermani* metacercariae in the Japanese freshwater crab *Geothelphusa dehaani* from the Kobado area of Chiba Prefecture, Japan. Medical Entomology and Zoology. 2018 Mar;69(1):1–5.

4. Hara T, Komeda Y, Fukuma T. Cases of nematode and trematode infection for which examination was requested to our laboratory in the last 10 years (Japanese). Nihon Rinsho Kiseichu Gakkai shi (Clinical Parasitology). 2020;31(1):23–7.

5. Harada T, Kawasaki Y, Tsukada A, Osawa Y, Takami H, Yamaguchi K, et al. Bronchodilator Reversibility Occurring during the Acute Phase of *Paragonimus westermani* Infection. Internal Medicine. 2019 Jan;58(2):297–300.

6. Hoshina T, Tamura K, Kawano S, Kato T, Sato F, Horino T, et al. Two Cases of *Paragonimiasis westermani* in a Chinese Family Diagnosed with the Ouchterlony Double Diffusion Method. Kansenshogaku Zasshi (The Journal of the Japanese Association for Infectious Diseases). 2014;88(6):866–70.

7. Ikehara M, Komase Y, Morita A, Yamaguchi H, Yamamoto T. Paragonimiasis in a person whose symptoms were shown 22 years after emigrating to Japan from Laos. Journal of Infection and Chemotherapy. 2010;16(1):49–52.

8. Ikuno H, Koyama J, Akao Nn. Considerations on encounters with parasites in 2014. Clinical Parasitology. 2016;27(1):57–9.

9. Inui A, Naito T, Sugihara E, Isonuma H. A Case of *Paragonimus westermani* Infection Diagnosed by Serological Testing. General Medicine. 2011;12(1):19–23.

10. Irie T, Yamaguchi Y, Doanh PN, Guo ZH, Habe S, Horii Y, et al. Infection with *Paragonimus westermani* of boar-hunting dogs in Western Japan maintained via artificial feeding with wild boar meat by hunters. Journal of Veterinary Medical Science. 2017;79(8):1419–25.

11. Ishihara M, Takakura A, Hiyoshi Y, Kasajima M, Kimura M, Kubota M, et al. A case of *Paragonimus westermani* Infection in a Laotian sister living in Japan. Clinical Parasitology. 2013;24(1):103–5.

12. Itoh N, Kurai H, Yamazaki H, Morisaki Y, Sugiyama H. A case of *Paragonimus westermani* infection during the follow-up period after lung cancer surgery (Japanese). Nihon Rinsho Kiseichu Gakkai shi (Clinical Parasitology). 2016;27(1):36–9.

13. Kagoshima T, Yamazaki Y, Sakaguchi K, Kubo K, Sugiyama H, Saito H. A Case of Two Female Siblings with *Paragonimus Westermani* Infection (Japanese). Nihon Naika Gakkai Zasshi (The Journal of the Japanese Society of Internal Medicine). 2014;103(4):975–7.

14. Kurai H. Summary of Parasitic Diseases Experienced at Cancer Centers (Japanese). Nihon Rinsho Kiseichu Gakkai shi (Clinical Parasitology). 2019;30(1):71–3.

15. Kuratomi S, Nakatsubo S, Sawamoto Y, Fujii A, Hidaka M, Misumi Y, et al. A Case Report of Pulmonary *Paragonimus westermani* infection with Bronchial Brushing Cytology (Japanese). Kikanshigaku (The Journal of the Japan Society for Respiratory Endoscopy). 2010;32(2):191–4.

16. Kuriyama M, Mrishita S, Araki J, Ishihara A, Maeda H. A case of *Paragonimus westermani* Infection that took 2 years from the appearance of pleural effusion to diagnosis (Japanese). Nihon Kokyuki Geka Gakkaiasshi (The Journal of the Japanese Association for Chest Surgery). 2019;8(6):391–5.

17. Mashima S, Hara S, Imase R, Jin Y, Matsubara O, Miyazaki Y. A case of *Paragonimus westermani* Infection in which immunological diagnostics were useful in differentiating from lung cancer (Japanese). Nihon Kokyuki Geka Gakkaiasshi (The Journal of the Japanese Association for Chest Surgery). 2020;9(1):38–42.

18. Matsui H, Takeda M, Asai K. A case of *Paragonimus westermani* Infection in a Chinese resident in Japan (Japanese). Nihon Kokyuki Geka Gakkaizasshi (The Journal of the Japanese Association for Chest Surgery). 2019;8(5):327–31.

19. Matsumoto H, Koyanagi A, Nakamura A. A case report of *Paragonimus westermani* infection differentially diagnosed from primary lung cancer (Japanese). Nihon Kokyuki Geka Gakkaiasshi (The Journal of the Japanese Association for Chest Surgery). 2016;30(7):821–6.

20. Matsumoto K, Ichimiya H. Pulmonary *Paragonimus westermani* infection with adjacent lobe invasion and false-positive fluorodeoxyglucose positron emission tomography mimicking primary lung cancer (Japanese). Nihon Kokyuuki Geka Gakkaizasshi (The Journal of the Japanese Association for Chest Surgery). 2018 Jul;32(5):580–6.

21. Matsuo K, Moribe J, Takashima Y, Kasuya S, Yoshida A, ABE N, et al. Possibility of Paragonimiasis Due to Consumption of Raw Deer Meat (Japanese). Nihon juuishikai Zasshi (Journal of the Japan Veterinary Medical Association). 2018 Aug;71(8):449–53.

22. Minezaki S, Hiroma T, Shiono A, Masumoto A, Mio T, Utsugi H, et al. A Case of Lung Paragonimiasis Superinfection with Hookworm Presenting Difficulty in Discrimination (Japanese). Kansenshogaku Zasshi (The Journal of the Japanese Association for Infectious Diseases). 2013;87(6):756–60.

23. Miyagawa H, Nagai H, Akagawa S, Masuda K, Tamura A, Ohta K. A Case of *Paragonimus westermani* infection with Cavity Enlargement after Praziquantel Therapy (Japanaes). Nihon Kokyuki Geka Gakkaizasshi (The Journal of the Japanese Association for Chest Surgery). 2016;5(6):356–60.

24. Miyano H, Kanzaki M, Yoshikawa T, Ohara T, Ohnuki K. *Paragonimus westermani* infection with bilateral pneumothoraces in a married couple (Japanese). Nihon Kikyou Nouhousei Haishikkan kanjagakkai Zasshi (The Journal of the Japam Society for Pneumothorax and Cystic Lung Diseases). 2010;10(2):120–3.

25. Nagano T, Noda S, Kaneko Y, Horikawa Y, Ishigami S, Matsugoe S. Peritoneal Cavity Abscess Due To *Paragonimus westermani* -A Case Report (Japanese). Nihon Rinsho Geka Gakkai Zasshi (Journal of Japan Surgical Association). 2010;71(3):823–7.

26. Nagata T, Mukae H, Futsugi Y, Sakito O, Hayashi Y, Sakamoto N, et al. A case of Paragonimus westermani Infection complicated by pyothorax due to Streptococcus pneumoniae (Japanese). Nihon Kokyuki Geka Gakkaiasshi (The Journal of the Japanese Association for Chest Surgery). 2010;48(4):288–92.

27. Nagayasu E, Yoshida A, Hombu A, Horii Y, Maruyama H. Paragonimiasis in Japan: A Twelve-year Retrospective Case Review (2001-2012). Internal Medicine. 2015;54(2):179–86.

28. Nagayasu H, Yoshida A, Honbu E, Kuroki M, Maruyama H. Recent Trends of Serodiagnosis in Paragonimiasis. Clinical Parasitology. 2013;24(1):100–2.

29. Nakamura K, Ichiyasu H, Tokunaga K, Horio Y, Tanaka H, Kohrogi H. A Report on 2 Cases of *Paragonimus westermani* Infection Caused by Eating Raw Boar Meat (Japanese). Kikanshigaku (Japan Society for Bronchology). 2012;34(6):552–7.

30. Nureki Shin-ichi, Ishii K, Fujisaki H, Torigoe M, Maeshima K, Shibata H, et al. Familial Mediterranean Fever with Rheumatoid Arthritis Complicated by Pulmonary Paragonimiasis. Internal Medicine. 2016;55(19):2889–92.

31. Ogata H, Harada E, Moriya S, Fukuyama S, Suzuki K, Shiraishi Y, et al. Pleuropulmonary Paragonimiasis with Multiple Nodules in the Pleura. Internal Medicine. 2020 Aug;59(15):1879–81.

32. Oh IJ, Kim YI, Chi SY, Ban HJ, Kwon YS, Kim KS, et al. Can Pleuropulmonary Paragonimiasis be Cured by Only the 1st Set of Chemotherapy? Treatment Outcome and Clinical Features of Recently Developed Pleuropulmonary Paragonimiasis. Internal Medicine. 2011;50(13):1365–70.

33. Ohari Y, Suzuki Y, Shibahara T, Itagaki T. First report of Paragonimus skrjabini miyazakii metacercariae in Geothelphusa dehaani (Sawagani) occurring in Iwate Prefecture, Japan. J Vet Med Sci. 2019 Aug 9;81(8):1109–12.

34. Ohuchi M, Inoue S, Ozaki Y, Fujita T, Ueda K, Hanaoka J. A case of *Paragonimus westermani* infection caused by eating raw deer meat (Japanese). Nihon Kokyuki Geka Gakkaizasshi (The Journal of the Japanese Association for Chest Surgery). 2014;28(2):170–6.

35. Okamoto H, Uramoto H, Mimura Y, Hoshida Y. Two cases of paragonimiasis verified by the presence of eggs in sputum cytology specimens (Japanese). Nihon Rinsho Saibou gakkaizasshi (The Journal of the Japanese Society of Clinical Cytology). 2014;53(4):292–7.

36. Onoe R, Komase Y, Usuba A, Hida N, Mineshita M. Two Cases of *Paragonimus westermani* Infection with Pulmonary and Brain Lesions. Journal of St Marianna University. 2017;8(2):103–9.

37. Otsuji N, Hirata H, Shiromori S, Satoh K, Sugitachi K, Arihuku H, et al. A Case of Paragonimiasis with nodular shadow and pleural effusion with Eggs in Sputum (Japanese). Nihon Rinsho Kiseichu Gakkai shi (Clinical Parasitology). 2019;30(1):95–7.

38. Satoh R, Misumi Y, Uemi Y, Shimokawa T, Hida N, Okamoto H, et al. A Case Report of *Paragonimus westermani* symptom with Multiple Cavitary Shadows in Both Lungs During Steroid Therapy for idiopathic hypereosinophilic syndrome (Japanese). Nihon Rinsho Kiseichu Gakkai shi (Clinical Parasitology). 2012;23(1):53–6.

39. Sim YS, Lee JH, Hong SC, Chang JH, Kang SR, Yang HJ, et al. *Paragonimus westermani* Found in the Tip of a Little Finger (Japanese). Internal Medicine. 2010;49(15):1645–8.

40. Sudo N, Hara Y, Yamaguchi N, Kudo M, Kaneko T. A case of *Paragonimus westermani* infection diagnosed by thoracoscopic pleural biopsy (Japanese). Nihon Kokyuki Geka Gakkaiasshi (The Journal of the Japanese Association for Chest Surgery). 2019;8(2):108–12.

41. Sueda R, Tominaga M, Koga Y, Sanada H, Sawamoto R, Hiyama J. Three Cases of *Paragonimus westermani* infection Treated in Our Hospital. Nihon Kyobu Rinsho (The Japanese Journal of Chest Diseases). 2014;73(11):1369–75.

42. Sugano K, Akai M, Katoh T, Tada T, Watanabe T, Shiozaki K. A case of *Paragonimus westermani* Infection with iatrogenic right and left pleural effusions (Japanese). Kikanshigaku (The Journal of the Japan Society for Respiratory Endoscopy). 2013;35(3):305–9.

43. Sugiyama H, Shibata K, Morishima Y, Muto M, Yamasaki H, Kawakami Y. Current Status of Lung Fluke Metacercarial Infection in Freshwater Crabs in the Kawane Area of Shizuoka Prefecture, Japan. Journal of Veterinary Medical Science. 2013;75(3):249–53.

44. Sugiyama H, Shibata K, Kawakami Y, Arakawa K, Morishima Y, Yamasaki H, et al. Paragonimiasis Due to the Consumption of Wild Boar Meat in Japan: Contamination Levels of Lung Fluke Larvae in Muscle Samples of Wild Boars Caught in Kagoshima Prefecture. Jpn J Infect Dis. 2015;68(6):536–7.

45. Sugiyama H, Shibata K, Kawakami Y, Gagawa C, Morishima Y, Yamazaki H, et al. Symptoms in Cases of Paragonimiasis Occurring in Japan and Analysis from Case Reports of Test Specimens. Nihon Rinsho Kiseichu Gakkaishi (Clinical Parasitology). 2015;26(1):65–7.

46. Sugiyama H, Shibata K, Kawakami Y, Otomoda M, Morisaki Y, Yamasaki H. Infection risk of Paragonimiasis through wild game meat (Japanese). Nihon Rinsho Kiseichu Gakkaishi (Clinical Parasitology). 2016;27(1):40–2.

47. Suzuki Y, Obata S, Shimabukuro K, Matsushima A, Mukae H. A case of a Japanese traveler infected with *Paragonimus westermani* in Zhejiang Province, China (Japanese). Nihon Kyobu Rihsho (The Japanese Journal of Chest Diseases). 2011;70(9):950–4.

48. Tabuchi K, Takahashi T, Adachi S, Tatebayashi H, Yajima T, Ohnishi R, et al. A family case of *Paragonimus westermani* Infection after eating raw deer meat (Japanese). Shonika Rinsho (Japanese Journal of Pediatrics). 2010;63(8):1798–802.

49. Takagi Y, Suganua A, Fujita A, Sakashita K, Miyamoto M, Murata K, et al. Four cases of *Paragonimus westermani* Infection in a group of Thai women living in Japan. Clinical Parasitology. 2010;20(1):40–2.

50. Tanaka K, Naoki K, Senno K, Ishioka K, Sayama K, Becchaku T. A case of *Paragonimus westermani* Infection diagnosed by sputum speculum examination (Japanese). Nihon Kokyuki Geka Gakkaiasshi (The Journal of the Japanese Association for Chest Surgery). 2013;2(5):633–6.

51. Tanimura K, Kita H, Kanemitsu S, Hushiya Y, Katayama Y, Nishihara H, et al. A case of *Paragonimus westermani* Infection with FDG-PET showing pulmonary nodule shadows and increased accumulation in the right pulmonary hilar and mediastinal lymph nodes (Japanese). Nihon Kokyuki Gakkai zasshi (The journal of the Japanese Respiratory Society). 2011;49(4):293–7.

52. Terashima G, Takeuchi E, Nishio H, Ishikawa M, Yamamoto T, Araki J, et al. A Case Report of Paragonimiasis infected with *Paragonimus miyazakii* in Lung Resection (Japanese). Nihon Rinsho Kiseichu Gakkai shi (Clinical Parasitology). 2010;20(1):43–5.

53. Todo Y, Nagata T, Takiguti T, Otsuka S, Nagayasu H, Maruyama H. A case of *Paragonimus westermani* Infection discovered by a right-sided abdominal subcutaneous mass (Japanese). Nihon Keiseigeka Gakkai Kaishi (Journal of Japan Society of Plastic and Reconstructive Surgery). 2019;39(1):30–6.

54. Tsuruoka J, Miyake T, Miyaji Y, Nakamura K, Hashimoto S, Tateyama S, et al. A case of *Paragonimus westermani* Infection led to cerebral lesions (Japanese). Shoni Kansen Meneki (The Journal of Pediatric Infectious Disease and Immunology). 2012;24(1):19–23.

55. Washino T, Kobayashi K, Ohta M, Hikone M, Sakamoto N, Iwabuchi S, et al. A case of *Paragonimus westermani* Infection in a Nepalese in Japan. Clinical Parasitology. 2015;26(1):59–61.

56. Yasukouchi Y, Kosho N, Toyoda K, Taniai H, Ogawa E, Kainuma S, et al. Four Cases of *Paragonimus westermani* Infection (Japanese). Nihon Byouin Sougousinryou Igakukai Zasshi (Journal of Hospital General Medicine). 2012;3(2):65–70.

57. Yatera K, Hanaka M, Hanaka T, Yamasaki K, Nishida C, Kawanami T, et al. A rare case of paragonimiasis miyazakii with lung involvement diagnosed 7 years after infection: A case report and literature review. Parasitology International. 2015 Oct;64(5):274–80.

58. Yoshida A, Tanaka K, Kikuchi Y, Nagayasu H, Maruyama H. Review of sources of Paragonimiasis in Japan (Japanese). Nihon Rinsho Kiseichu Gakkai shi (Clinical Parasitology). 2016;27(1):49–51.

59. Yoshimatsu Y, Nakabachi S, Sugiyama H, Tomioka E, Horio J, Satoh M, et al. A Case of *Paragonimus haterotremus* infection in a Myanmar resident in Japan (Japanese). Nihon Rinsho Kiseichu Gakkai shi (Clinical Parasitology). 2013;24(1):106–8.

*Fascioliasis (12)*

1. Kurokawa T, Ueda T, Enomoto T, Yamamoto M, Maruyama H, Ohkohchi N. A Suspected Case of Fascioliasis Preoperatively Diagnosed as Intrahepatic Bile Duct Cancer. The Japanese Journal of Gastroenterological Surgery. 2012;45(4):387–93.

2. Matsuo K, Takai N, Tanaka E, Aoki E, Takashima Y. Current status of fasciolosis among cattle in Japan, especially Japanese Black breeding cattle (Japanese). Kachiku Kansensho Gakkaishi (The Journal of Farm Animal in Infectious Disease). 2020;9(1):1–8.

3. Mori S, Mitsuhashi K, Suzuki M, Hagiwara K, Asakawa M. Distribution of the endoparasite fauna of Yezo Deer (*Cervus nippon yesoensis*) and that of Fasciola sp. parasitizing the Hokkaido Yezo Deer in Hidaka District, Hokkaido Prefecture. Hokkaido Juishikai Zasshi (The Journal of the Hokkaido Veterinary Medical Association). 2014;58(2):44–7.

4. Nakamura F, Sahara T, Tatomi A, Ohnishi K, Igarashi Y, Mizobuchi R, et al. A case of imported fasciolosis diagnosed by paired serum antibody titers. Clinical Parasitology. 2018;29(1):46–8.

5. Nakayama K, Akao N, Ohta N. Parasitic infections in foreigners living in Japan: estimation of parasitic infection history by serum antibody screening. J Med Dent Sci. 2011 Sep 30;58(3):97–102.

6. Ohari Y, Oshida T. Survey on Prevalence of Fosciola sp. in Sika Deer （*Cervus nippon yesoensis*） in Tokachi District, Hokkaido, Japan. Japanese Journal of Zoo and Wildlife Medicine. 2013;18(4):115–20.

7. Okajima J, Shibata K, Takahashi E, Nagafuchi T, Okajima K, Nonaka N. Current status and its epidemiological consideration of *Fasciola* and *Eurytrema* infections in beef cattle of Japan. Journal of Veterinary Medical Science. 2016;78(5):785–90.

8. Sakurai T, Watanabe Y, Inui A, Oshima H, Isonuma H, Dambara T, et al. A case of human fascioliasis infected from eating watercress in Japan. Nihon Byoin Sogoshinryo Igakukai Zasshi (Japanese Journal of Hospital General Medicine). 2013;5(2):62–6.

9. Sato Y, Takasaka Y. A Case of Fascioliasis Suspected of being a Malignant Tumor. Nihon Gekakei Rengo Gakkaishi (Journal of Japanese College of Surgeons). 2011;36(5):850–5.

10. Shimada M, Yamamoto D, Hayakawa S, Matsuoka H. A review of parasite-related cases experienced in the Department of Medical and Animal Science, Jichi Medical University from 2011 to 2014. Jichiikadaigaku Kiyo (Jichi Medical School Journal). 2016;38:71–7.

11. Takahashi A, Itaxazwa T, Adachi Y, Miyawaki T. A case of liver fluke Infection that was difficult to distinguish from echinococcus (Japanese). Nihon Shonika Gakkai Zasshi (The Journal of the Japan Pediatric Society). 2011;115(11):1781–5.

12. Yamanaka Y, Takeuchi M. A Case of fascioliasis. Kanzo. 2020 Nov;61(11):607–12.

*Opisthorchiasis (0)*

**Human African trypanosomiasis (1)**

1. Kita K. African sleeping sickness. Japanese Journal of Pediatrics. 2017;70:2283–8.

**Leishmaniasis (12)**

1. Ben Salah A, Ben Messaoud N, Guedri E, Zaatour A, Ben Alaya N, Bettaieb J, et al. Topical Paromomycin with or without Gentamicin for Cutaneous Leishmaniasis. New England Journal of Medicine. 2013 Feb 7;368(6):524–32.

2. Hirayama K. Parasites: trypanosomiasis and leishmaniasis. Nihon Iji Shimpo (Japan Medical Journal). 2019;(4976):22–4.

3. Imai K, Tarumoto N, Amo K, Takahashi M, Sakamoto N, Kosaka A, et al. Non-invasive diagnosis of cutaneous leishmaniasis by the direct boil loop-mediated isothermal amplification method and MinION™ nanopore sequencing. Parasitol Int. 2018 Feb;67(1):34–7.

4. Ito K, Takahara M, Ito M, Oshiro M, Takahashi K, Uezato H, et al. An imported case of cutaneous leishmaniasis caused by Leishmania (Leishmania) donovani in Japan. Vol. 41, The Journal of Dermatology. 2014. p. 926–8.

5. Kato T. Zoonosis - leishmaniasis. Shonika Rinsho (Japanese Journal of Pediatrics). 2017;70(Suppl):2272–82.

6. Kawamura Y, Yoshikawa I, Katakura K. Imported leishmaniasis in dogs, US military bases, Japan. Emerg Infect Dis. 2010 Dec;16(12):2017–9.

7. Matsuura H, Utsumi D, Fujimoto W, Mitekura H, Urishihara K, Uezato H. A case of a cutaneous leishmaniasis patient who was infected in Brazil. Vol. 72, Nishi Nihon Hifuka (The Nishinihon Journal of Dermatology). 2010. p. 116–20.

8. Okumura Y, Yamauchi A, Nagano I, Itoh M, Hagiwara K, Takahashi K, et al. A case of mucocutaneous leishmaniasis diagnosed by serology. J Dermatol. 2014 Aug;41(8):739–42.

9. Ono M, Takahashi K, Taira K, Uezato H, Takamura S, Izaki S, et al. Cutaneous leishmaniasis in a Japanese returnee from West Africa successfully treated with liposomal amphotericin B. Vol. 38, The Journal of Dermatology. 2011. p. 1062–5.

10. Sanzyoba C, Matsumoto Y. Autochthonous sand flies in Japan. Vol. 9, Zyui Kiseichu Kaishi (Jpn. J. Vet. Parasitol.). 2011. p. 104–9.

11. Tokoro M, Nakamoto K, Arai T, Kaneko Y, Hayashi N, Ueda M. A case of a visceral leishmaniasis patient coinfected with HIV. Vol. 20, Clinical Parasitology. 2010. p. 96–8.

12. Tsujimoto K, Nakagawa T, Uno K, Yamada Y, Ogawa T, Kasahara K, et al. A case of a cutaneous leishmaniasis patient who developed symptoms in Brazil. Vol. 23, Clinical Parasitology. 2012. p. 34–6.

**Leprosy (88)**

1. Baba M. A case of a former Hansen’s disease patient with mixed dementia in whom support for oral intake led to a change in the family’s concern. Vol. 22, Nihon Sesshoku Enge Rehabilitation Gakkai Zasshi (The Japanese Journal of Dysphagia Rehabilitation). 2018. p. 267–72.

2. Baba M, Nurse and Care Staff in Handicapped Person’s Ward, NST Committee, Bedsore Committee. Successful team medicine to preserve foot function in intractable ulcer complicated by diabetes and the sequelae of Hansen’s disease. Japanese Journal of Foot Care. 2017;15(3):135–8.

3. Cho S. Milestone of movement by Japan Federation of Democratic Medical Institutions: establishment of ‘Leprosy Prevention Act’ - abused patient rights (1). Miniren Iryo. 2018. p. 58–61.

4. Eto F. Temptation of history (35th): Leprosy Prevention Act after World War II. Vol. 25, Journal of Clinical Rehabilitation. 2016. p. 1119–24.

5. Fukai K, Komatsu K, Matsuo Y, Hayashi K, Kariya Y, Miyagi T, et al. Two cases of squamous cell carcinoma arising on refractory skin ulcers in patients with a history of Hansen’s disease. Nishi Nihon Hifuka. 2019;81(2):115–9.

6. Fukunishi M. Act Number 11: Prevention of leprosy and the beggining of isolation - establishement of Hokubu Sanatorium. Seminar Iryo to Shakai (Journal of Health Care, Medicine and Community). 2011. p. 3–21.

7. Fukuoka Y. What does psychiatry learn from the fight of leprosy patients? - ‘narrative of anger’ and ‘narrative of thanks’ - an interview with leprosy recoverers. Vol. 59, Byoin Chiiki Seishin Igaku (Jpn. J. Hospital & Community Psychi). 2016. p. 24–7.

8. Fuzisaki M. What does psychiatry learn from the fight of leprosy patients? Vol. 59, Byoin Chiiki Seishin Igaku (Jpn. J. Hospital & Community Psychi). 2016. p. 22–4.

9. Gidoh M. The World Health Organization (WHO) and Japan’s multiple drug therapy (MDT). Kokuritsu Hansenbyo Shiryokan Kenkyu Kiyo (Research Bulletin, National Hansen’s Disease Museum). 2010. p. 66–8.

10. Goto M, Nogam R, Okano Y, Gidoh M, Yotsu R, Ishida Y, et al. Guidelines for the treatment of Hansen’s disease in Japan (third edition). Nihon Hansenbyo Gakkai Zasshi. 2013 Dec;82(3):143–84.

11. Harada M. 100 years of the National Sanatoriums in Japan. Nihon Hansenbyo Gakkai Zasshi. 2010 Feb;79(1):11–6.

12. Hasegawa K. What does psychiatry learn from the fight of leprosy patients? - problems of psychiatry revealed from the claim for government compensation for Leprosy Prevention Act (the judgment of Kumamoto Local Court). Vol. 59, Byoin Chiiki Seishin Igaku (Jpn. J. Hospital & Community Psychi). 2016. p. 27–9.

13. Hirokawa W. Hansen’s Disease Patients in Yunosawa Village and Their Relationship with the Wider Community: 1869-1941. Nihon Hansenbyo Gakkai Zasshi. 2016 Aug;85(2):75–8.

14. Hosoda M. Hansen’s disease recoverers as agents of change: a case study in Japan. Lepr Rev. 2010 Mar;81(1):5–16.

15. Igarashi Y. Topics on medical law: arguments around the bill to protect leprosy patiens and their family members. Vol. 35, Iji Hougaku (Journal of Medical Law). 2020. p. 232–7.

16. Iha H. The structure of practices and tasks of death watch care of residents in the a national sanatorium for Hansen’s disease. Vol. 85, Nihon Hansenbyo Gakkai Zasshi (Japanese Journal of Leprosy). 2016. p. 123–32.

17. Ikai T. The Establishment of Japan’s Policy for Hansen’s Disease and the Patients. Japanese Journal of Leprosy. 2017 Dec;86(2):101–6.

18. Inaba T. Historical Overview of Leprosy Control in Japan. Kokuritsu Hansenbyo Shiryokan Kenkyu Kiyo (Research Bulletin, National Hansen’s Disease Museum). 2019. p. 1–15.

19. Inoue H. Hansen’s disease is not over: issues around ‘special court’. Miniren Iryo. 2017. p. 56–60.

20. Ishida Y, Inoue T, Tsuchiya I, Maeda M, Hirano A. Two cases of leprosy related conditions and relevant issues on medical services for ex-leprosy patients who live in communities in Japan. Nihon Hansenbyo Gakkai Zasshi. 2010 Feb;79(1):3–10.

21. Ishii N. Thalidomide-the way to be covered by health insurance when used for treatment of ENL. Vol. 79, Nihon Hansenbyo Gakkai Zasshi (Japanese Journal of Leprosy). 2010. p. 275–9.

22. Ishii N. Curernt status of Hansen’s disease. Vol. 17, Kochishi Ishikai Igaku Zasshi (Journal of Kochi Medical Association). 2012. p. 12–8.

23. Ishii N. Hansen’s disease and dermatology. Vol. 81, Nishi Nihon Hifuka (The Nishinihon Journal of Dermatology). 2019. p. 461–4.

24. Ishii N, Ishida Y, Okano Y, Ozaki M, Gidoh M, Kumano K, et al. Japanese guideline on thalidomide usage in the management of erythema nodosum leprosum. Vol. 80, Nihon Hansenbyo Gakkai Zasshi (Japanese Journal of Leprosy). 2011. p. 275–85.

25. Ishii N, Ishida Y, Okano Y, Ozaki M, Gidoh M, Kumano K, et al. Guideline for clinical use of thalidomide for management of erythema nodosum leprosum in Japan. Vol. 86, Nihon Hansenbyo Kaishi (Japanese Journal of Leprosy). 2017. p. 91–100.

26. Ishii N, Ono T. Efforts towards expanding health services for Hansen’s disease. Vol. 120, Nihon Hifuka Gakkai Zasshi (The Japanese Journal of Dermatology). 2010. p. 1673–4.

27. Ishii N, Yotsu RR, Mori S. Leprosy outpatient clinic in Aichi Prefecture, Japan. Nihon Hansenbyo Gakkai Zasshi. 2011 Sep;80(3):261–8.

28. Ishizaki T, Ogura S, Yamada M, Shiroi T, Takamori H. Experience of implant treatment in a leprosy sanatorium. Vol. 10, Japanese Journal of Maxillo Facial Implants. 2011. p. 231–5.

29. Isobe A. Topics on medical law: establishment of Act to Accelerate the Resolution of the Hansen’s Disease Problems. Vol. 25, Iji Hougaku (Journal of Medical Law). 2010. p. 210–5.

30. Iwai S. Efforts towards leprosy at Japan Medical Welfare Society. Vol. 48, Iryo to Fukushi (The Japanese Journal of Social Workers in Health Services). 2014. p. 19–22.

31. Iwata M. Women who fought against leprosy. Vol. 20, Zyosei Shinsin Igaku (Journal of Japanese Society of Psychosomatic Obstetrics and Gynecology). 2016. p. 261–7.

32. Iwata M, Matsumura Y, Ozaki M, Nakanaga K, Hoshino Y, Ishii N, et al. Relapsed Leprosy with Infiltrative Ring-shaped Erythematous Plaques Seen in a Brazilian 16 Years after Multi-drug Therapy. Hifu no Kagaku. 2014;13(6):426–30.

33. Joko S, Horie D. A case of a patient with a history of leprosy-related uveitis to whom cataract surgery was performed after phototherapeutic keratectomy. Vol. 24, Ganka Syuzyutsu (Japanese Journal of Ophthalmic Surgery). 2011. p. 199–203.

34. Kagaya N. How the Japanese people have dealt with taking measures against Hansen’s disease in Japan: from the perspective of the charity work of the Japanese royal family and some religious groups. Vol. 8, Hirosaki Gakuin Daigaku Kango Kiyou (Bulletin of Faculty of Nursing Hirosaki Gakuin University). 2013. p. 65–71.

35. Kato T. Travel medicine: symptom-based examination of health problems after returning to Japan - rash. Vol. 119, Naika (Internal Medicine). 2017. p. 987–93.

36. Kawaguchi A. Narrative of having a hard life. Vol. 16, Addiction Kango (Japanese Journal of Study in Addiction Nursing). 2019. p. 97–101.

37. Kawaguchi A, Watanabe K, Yoshimura T, Date K, Yamashita K. The current status of dementia in admitions to national leprosariums nationwide. Vol. 83, Nihon Hansenbyo Gakkai Zasshi (Japanese Journal of Leprosy). 2014. p. 117–24.

38. Kitahara M. Peculiar history to formation of ‘Freely recuperate zone’ in the National Sanatorium Kuryu Rakusen-en, Gunma, Japan (relation between the ‘Old Yunosawa-ward’ and ‘Freely recuperate zone’ in the National Sanatorium Kuryu Rakusen-en). Nihon Hansenbyo Gakkai Zasshi. 2011 Sep;80(3):249–59.

39. Koba A, Mori S, Ishii N. Survey of newly diagnosed Japanese leprosy patients. Nihon Hansenbyo Gakkai Zasshi. 2011 Feb;80(1):11–6.

40. Koike H, Hashimoto R, Tomita M, Kawagashira Y, Iijima M, Nagamatsu M, et al. The wide range of clinical manifestations in leprous neuropathy: two case reports. Intern Med. 2011;50(19):2223–6.

41. Kosaka Y. The present of social relationships between Hansen’s disease sufferers and the others: national sanatorium Oshimaseishoen and Setouchi International Art Festival 2010. Vol. 24, Hoken Iryo Shakaigaku Ronsyu (The Japanese Journal of Health and Medical Sociology). 2014. p. 27–37.

42. Kosugiyama R, Kasai N, Etani T, Oshima A. Hearing loss in leprosarium: Current status. Japanese Journal of Leprosy. 2016;84(3):125–31.

43. Makino M. Learning from the history of leprosy--looking back at one hundred years of medicine at the leprosaria. Nihon Hansenbyo Gakkai Zasshi. 2010 Feb;79(1):25–36.

44. Masubuchi Y, Takayama H, Isogai R. How did people living in the leprosy sanatoria satisfy their needs? Nihon Kango Gakkai Ronbunsyu: Seizin Kango II. 2010. p. 251–3.

45. Matsuki T, Okano Y, Aoki Y, Ishida Y, Hatano K, Kumano K. Effectiveness of thalidomide for erythema nodosum leprosum. Vol. 83, Nihon Hansenbyo Gakkai Zasshi (Japanese Journal of Leprosy). 2014. p. 111–6.

46. Matsutani Y. Implications of the modern history of Hansen’s disease in Japan. Hokkaido Igaku Zasshi. 2013 Jan;88(1):7–9.

47. Matsuura T. Segregation: problems with the national policy on Hansen’s disease (part 1). Vol. 24, Shakai Rinsho Zasshi (The Shakai Rinsho Review). 2016. p. 78–94.

48. Matsuyama M, Kawamoto S. Analysis of Leprosy stigma. Vol. 8, Fukushi Shinrigaku Kenkyu. 2011. p. 45–53.

49. Mori S, Ato M, Ishii N. A study on the entering and out-going trends at Japan’s national Hansen’s disease sanatoriums. Nihon Hansenbyo Gakkai Zasshi (Japanese Journal of Leprosy). 2019;88(2):53–75.

50. Mori S, Ishii N. A study on the entering and out-going trends at Japan’s national Hansen’s disease sanatoriums. Nihon Hansenbyo Gakkai Zasshi. 2017 Apr;86(1):69–90.

51. Mori S, Yotsu RR, Suzuki K, Makino M, Ishii N. Present situation of leprosy in Japan, 2006-2010: analysis of drug resistance in new registered and relapsed cases by molecular biological methods. J Dermatol Sci. 2012 Sep;67(3):192–4.

52. Nakagawa M, Shimizu A. The characteristic of oral status and dental treatment of leprosy sequelae patients. Vol. 83, Nihon Hansenbyo Gakkai Zasshi (Japanese Journal of Leprosy). 2014. p. 95–100.

53. Nakagawa M, Shimizu A. The originality and creativity of leprosy sequelae patients in regard to dental care. Vol. 84, Nihon Hansenbyo Gakkai Zasshi (Japanese Journal of Leprosy). 2016. p. 119–24.

54. Nakamura F. Sociological reflection on some realities and risks to accommodate the patients with Hansen’s disease from Amami and Okinawa islands. Yamaguchi Kenritsu Daigaku Gakuzyutsu Zyoho (Archives of Yamaguchi Prefectural University). 2017. p. 33–58.

55. Nakamura I, Yamaguchi S, Kariya Y, Matori S, Taira K, Yamamoto Y, et al. Hansen’s Disease of Lepromatous Type Occurring in a Micronesian US Soldier. Nishi Nihon Hifuka. 2013;75(4):326–30.

56. Namisato M. Leukoderma acquisitum centrifugum (halo nevus) developed following type 1 leprosy reaction. Japanese Journal of Leprosy. 2018;86(3):175–9.

57. Namisato M, Fujiwara T. Two cases of leprosy who had drug resistance related mutations to diaphenylsulfone, rifampicin and new quinolone. Nihon Hansenbyo Gakkai Zasshi (Japanese Journal of Leprosy). 2018;86(3):181–7.

58. Narita M. Prejudice and discrimination surrounding Hansen’s disease/leprosy. Kokuritsu Hansenbyo Shiryokan Kenkyu Kiyo (Research Bulletin, National Hansen’s Disease Museum). 2010. p. 1–10.

59. Narita M. Concerning the purpose of national Hansen’s disease museum: restoration of honor. Kokuritsu Hansenbyo Shiryokan Kenkyu Kiyo (Research Bulletin, National Hansen’s Disease Museum). 2011. p. 1–7.

60. Narita M. Why has Japan’s policies toward leprosy been able to proceed this far? Kokuritsu Hansenbyo Shiryokan Kenkyu Kiyo (Research Bulletin, National Hansen’s Disease Museum). 2012. p. 1–16.

61. Ng W, Ikeda S. The literary works of Hansen’s disease patients in Japan. Int J Dermatol. 2010 Apr;49(4):461–3.

62. Nishiguchi M, Watanabe Y, Kaminaka C, Furukawa F, Komori R, Yasui M, et al. A japanese case of newly-developed multibacillary leprosy initially diagnosed as sarcoidosis. The Japanese Journal of Dermatology. 2016;126(13):2433–9.

63. NIshiura N. The situation over reintegration into society of young residents of Leprosy sanatoria : from 1950 to 1970, in Japan. Kokuritsu Hansenbyo Shiryokan Kenkyu Kiyo (Research Bulletin, National Hansen’s Disease Museum). 2013. p. 19–41.

64. Noda M, Tsunemi Y, Masui Y, Yoshizaki A, Ohmatsu H, Takazawa Y, et al. Leprosy with spontaneous type 1 lepra reaction as an initial cutaneous manifestation: a case from Japan. Eur J Dermatol. 2018 Dec 1;28(6):843–4.

65. Nogami R. Problems left behind around leprosy. Vol. 121, Nihon Hifuka Gakkai Zasshi (The Japanese Journal of Dermatology). 2011. p. 2707–9.

66. Nogami R. The present conditions and future of National Sanatorium Kikuchi Keifuen. Nihon Hansenbyo Gakkai Zasshi. 2015 Sep;84(2):73–7.

67. Ogura T. Support for childbirth and parenting by leprosy patients under the Eugenic Protection Act: an interview-based study in Amami Wako En. Tokyo Tushin Daigaku Kiyou (Journal of Tokyo Online University). 2020. p. 187–93.

68. Ohira A, Yamaguchi S, Arakaki O, Okubo Y, Miyagi T, Takahashi K, et al. A Case Report of Borderline Lepromatous Type Leprosy in a Japanese Elderly Person. Nishi Nihon Hifuka. 2015;77(5):497–502.

69. Ohyama H, Hongyo H, Shimizu N, Shimizu Y, Nishimura F, Nakagawa M, et al. Clinical and Immunological Assessment of Periodontal Disease in Japanese Leprosy Patients. Vol. 63, Japanese Journal of Infectious Diseases. 2010. p. 427–32.

70. Okatani T. Human rights in mental health: for release from isolation at national leprosy sanatoria. Iryo Rodo. 2015. p. 18–9.

71. Oketani T. Current situation and challenges of national leprosy sanatoria. Iryo Rodo. 2014. p. 26–32.

72. Ozaki M. Challenges of medical services for leprosy: reflection on the transition of medicines for treatment. Nihon Hansenbyo Gakkai Zasshi (Japanese Journal of Leprosy). 2018;87(2):71–2.

73. Ozaki M, Tomoda M. Conjugal leprosy infection in Japan--case report and review. Nihon Hansenbyo Gakkai Zasshi. 2012 Apr;81(1–2):135–43.

74. Sano N. On the present and future of Japanese Leprosariums--the case of Oshima-seishoen Sanatorium. Nihon Hansenbyo Gakkai Zasshi. 2015 Sep;84(2):67–70.

75. Shigeyasu C, Nakashima T, Keino H, Ikeda K, Yamane みお, Horie D, et al. Clinical Science : State of Ocular Sequelae and Visual Dysfunction in Individuals with Hansen’s Disease. Vol. 123, J Jpn Ophthalmol Soc. 2019. p. 51–7.

76. Takahashi K. Current situations of diverse skin diseases in Okinawa and understanding of disease contexts. Vol. 88, Nihon Hansenbyo Gakkai Zasshi (Japanese Journal of Leprosy). 2019. p. 39–41.

77. Tanikawa T, Miyawaki H, Shinjyo H, Amano Y, Kondo M. The meaning of the experience of the everyday poverty of life for the oldest Hansen’s disease survivors. Vol. 84, Nihon Hansenbyo Gakkai Zasshi (Japanese Journal of Leprosy). 2015. p. 37–50.

78. Tsuruwaka M, Yokose R. Research concerning of nursing care for Hansen’s disease patients in Japan: based on the narratives of former Hansen’s disease sanatorium patients. Vol. 21, Rinsho Shiseigaku (japanese Journal of Clinical Thanatology). 2016. p. 25–34.

79. Ukai Y, Sugimoto T, Kawauchi Y, Ohi T, Fujii N, Nakanishi T, et al. Case reports: Filipino BL type Hansen’s disease that occurred in Japan. Vol. 59, Hifuka no Rinsho. 2017. p. 675–8.

80. Unai K. Understanding the ‘endless and invisible’ problems of Hansen’s disease. Vol. 19, Shakai Rinsho Zasshi (The Shakai Rinsho Review). 2011. p. 62–70.

81. Yamaberi M, Kondo M, Ishikawa K, Amano Y, Kondo M. Causes of and coping strategies for repeated wounds in patients with Hansen’s disease until promin treatment. Vol. 38, Nihon Kango Kenkyu Gakkai Zasshi (Journal of Japan Society of Nursing Research). 2015. p. 59–71.

82. Yamaguchi S, Takahashi K, Kamisato H. Tests of leprosy. Derma. 2014. p. 113–21.

83. Yokose R. The suffering of female ex-patients : from the narratives of women who have recovered from hansen’s disease. Bioethics. 2013;23(1):54–62.

84. Yokose R. The continued suffering of recovered Hansen’s Disease patients with regards to their future despite having achieved their rehabilitation : Narratives by those who left Hansen ’s disease sanatoriums with regards to their later abode. Bioethics. 2014;24(1):126–35.

85. Yokota Y. Leprosy and human rights: trends in japan and in the world. Nihon Hansenbyo Gakkai Zasshi (Japanese Journal of Leprosy). 2014;83(3):125–9.

86. Yotsu R. Chemoprophylaxis for preventing leprosy: a review. Vol. 84, Nihon Hansenbyo Gakkai Zasshi (Japanese Journal of Leprosy). 2015. p. 51–7.

87. Yotsu R. Tropical diseases in Japan - bacteria - leprosy. Nihon Iji Shimpo (Japan Medical Journal). 2019. p. 36–7.

88. Yotsu R, Suzuki K, Mori S, Ishii N. Diagnosis of Hansen’s disease. Nihon Hansenbyo Gakkai Zasshi (Japanese Journal of Leprosy). 2011;80(1):59–70.

**Lymphatic filariasis (2)**

1. Nagaoka F, Itoh Ma, Kadosaka T, Takagi H. Cases of suspected filarial infections in our laboratory in the past 5 years. Clinical Parasitology. 2015;26(1):68–70.

2. Ohtake S, Kurita H, Fujikawa N, Osada H, Nakashima Y, Itoh M, et al. A case of hydrocele testis associated with filarial infection (Japanese). Hinyoki Geka (Japanese Journal of Urological Surgery). 2016;29(2):165–8.

**Mycetoma (73)**

*Chromoblastomycosis (20)*

1. Fukuda T. [Skin care -from infants to the elderly-] Fungal infection and skin care. Derma. 2013. p. 62–7.

2. Hata Y. [Essential fungal disease diagnostics for the dermatologist].Diagnostic procedures for deep-seated dermatomycosis. Derma. 2011. p. 55–60.

3. Hayakawa R, Shimoda Y, Ida Y, Shimoyamada Y, Fukuda T, Oyama M. A case of black demarcation mycosis due to Exophiala dermatitidis with genital herpes-like appearance. Rinsho Hifuka (Japanese Journal of Clinical Dermatology). 2020;47(13):1071–6.

4. Hiruma M. The professionalism of cutaneous fungal infection treatment : Practice of oral antifungal therapy for cutaneous fungal infections. Vol. 124, Nihon Hifuka Gakkai Zasshi ( The Japanese Journal of Dermatology). 2014. p. 2701–2.

5. Iwasawa M. Understanding the pathogenesis and clinical presentation of granulomatous diseases Bacterial/fungal granulomas. Vol. 124, Nihon Hifuka Gakkai Zasshi (The Japanese Journal of Dermatology). 2014. p. 3099–101.

6. Kusuhara M. The Craftsmanship of dermatomycosis practice don’t forget to culture of cutaneous fungal infections. Vol. 124, Nihon Hifuka Gakkai Zasshi ( The Japanese Journal of Dermatology). 2014. p. 2694–7.

7. Kusuhara M. Current status of deep-seated cutaneous mycosis. Derma. 2018. p. 64–71.

8. Mitomo T, Ushigome Y, Fukuda T, Karino Y, Shiohara T. Two cases of chromomycosis identified as Fonsecaea monophora by molecular phylogenetic analysis. Vol. 57, Medical Mycology Journal. 2016. p. J133–9.

9. Mochiduki T, Anzai K. Dermatomycosis now and in the future deep-seated dermatomycosis up-to-date. Vol. 36, Nihon Rinsho Hifuka Ikai Zasshi (Journal of the Japan Organization of Clinical Dermatologists). 2019. p. 480–4.

10. Mochizuki T. [Infectious Disease Syndromes (2nd ed.)-Including syndromes to infectious mono-diseases-[Top]] Fungal infections Chromomycosis. Nihon Rinsho (The Japanese journal of clinical medicine). 2013. p. 612–7.

11. Mochizuki T, Anzawa K. Dermatomycosis Now and in the Future Deep-seated Dermatomycosis up-to-date. NIhon Rinho Hifuka Ikai Zasshi (Journal of the Japan Organization of Clinical Dermatologists). 2019;36(4):80–484.

12. Mochizuki T, Tsuboi Y, Igarashi K, Ishizaki J, Ushigami T, Ogawa H, et al. Japanese Society of Dermatology Guidelines for the treatment of dermatomycosis 2019. Vol. 129, Nihon Hifuka Gakkai Zasshi (The Japanese Journal of Dermatology). 2019. p. 2639–73.

13. Naka W. Educational Series Dermatomycosis : Chromomycosis. Vol. 53, Medical Mycology Journal. 2012. p. 225–31.

14. Sugiyama Y, Suzuki Y, Sugaya K, Tokura Y, Yaguchi T, Kamei K, et al. Chromoblastomycosis caused by Fonsecaea monophora. Med Mycol J. 2011;52(3):255–60.

15. Tanabe H. [How to dermatopathology] Infectious diseases of the skin: fungus and parasite. Derma. 2011. p. 117–22.

16. Taniguchi T, Tsuji M, Takei K, Takahara M, Anzai K, Matsuda T, et al. A case of chromoblastomycosis due to Fonsecaea monophora treated with surgical excision. Vol. 82, Nishi Nihon Hifuka. 2020. p. 289–93.

17. Ushigami T, Mochizuki T. [Essential fungal disease diagnostics for the dermatologist].Skin Biopsy - How to handle this specimen? -. Derma. 2011. p. 29–34.

18. Wakumono K, Yamamoto O. [All About Granulomas] Fungal disease. Derma. 2013. p. 53–7.

19. Watanabe S. [All Infectious Disease Guidelines] Other major academic societies: Japanese Dermatological Association and the Japanese Society of Medical Mycology, Guidelines for the diagnosis and treatment of dermatomycosis. Vol. 32, Kagaku Ryouhou No Ryoiki (Antibiotics & chemotherapy). 2016. p. 854–61.

20. Yanagihara S, Kobayashi H, Kamo R, Hirata C, Hiruma M, Nishimura K, et al. Chromoblastomycosis caused by Fonsecaea nubica: First report from Japan. J Dermatol. 2015 Aug;42(8):833–4.

*Paracoccidioidomycosis (14)*

1. Fujiwara H, Yamada H, Kishi Y, Kumagaya K, Nakaoka K, Hamada Y. A case of oral paracoccidioidosis that was difficult to differentiate from sarcoidosis. Vol. 61, Nihon Koukuugeka Gakkai Zasshi (Japanese Journal of Oral and Maxillofacial Surgery). 2015. p. 36–40.

2. Igami TZ, Solano F, Preti RC, Pagliari C, Takahashi WY, Hida RY, et al. Retinal involvement of Paracoccioidomycosis: A Case Report. Vol. 40, Tropical Medicine and Health. 2012. p. 149–53.

3. Izumikawa K. [Unknown fever and infection] Fungal infection. Vol. 106, Nihon Naika Gakkai Zasshi (The Journal of the Japanese Society of Internal Medicine). 2017. p. 2333–40.

4. Kamei K. [Immunological Tests: How to Read the Numbers] Immunological tests infectious disease-related tests (Including antigens and antibodies) Nonviral infectious diseases Coccidioidosis and Paracoccidioidosis. Vol. 68, Nihon Rinsho (The Japanese journal of clinical medicine). 2010. p. 267–70.

5. Kamei K. Progress in diagnosis and treatment of infectious diseases] understanding the pathogenesis of imported fungal diseases: The rise of imported mycosis. Vol. 99, Nihon Naika Gakkai Zasshi (The Journal of the Japanese Society of Internal Medicine). 2010. p. 2689–95.

6. Kamei K, Watanabe T. Infectious disease syndromes (2nd Edition)-Including syndromes to infectious mono-diseases-[1] Fungal infections Paracoccidioidosis. Vol. separate volume, Nihon Rinsho (The Japanese journal of clinical medicine). 2013. p. 583–5.

7. Kamei K.,. File No.100 Imported fungal diseases and their countermeasures. Journal of New Remedies & Clinics. 2017;66(2):187–92.

8. Kurai H, Ohmagari N, Ito K, Kawamura I, Suzuki J, Hadano Y, et al. [Case of oral paracoccidioidomycosis suspected to be pharyngeal cancer]. Med Mycol J. 2012;53(1):49–52.

9. Nishikawa T. Knowledge of dermatomycosis and medical fungi required for dermatologists. Vol. 56, Medical Mycology Journal. 2015. p. J15–21.

10. Onda H, Komine M, Murata S, Ohtsuki M. Letter: Imported paracoccidioidomycosis in Japan. Dermatol Online J. 2011 Dec;17(12):11.

11. Wanatabe T, Kamei K. [Infectious Diseases: Advances in Diagnosis and Treatment] Infectious diseases in the spotlight: Current status and countermeasures for imported mycosis. Vol. 103, Nihon Naika Gakkai Zasshi (The Journal of the Japanese Society of Internal Medicine). 2014. p. 2674–9.

12. Watanabe T, Kamei K. Import fungal disease. Vol. 31, Kokyu. 2012. p. 1106–10.

13. Watanabe T, Kamei K. Current status of imported fungal infections and countermeasures. Nihon Naika Gakkai Zasshi (The Journal of the Japanese Society of Internal Medicine). 2014. p. 2074–679.

14. Yoshimura Y, Tachikawa N, Oosawa T, Kosuge Y, Kamei K. [A case of paracoccidioidomycosis with severe adrenal insufficiency]. Kansenshogaku Zasshi. 2012 May;86(3):291–4.

*Sporotrichosis (34)*

1. Fukuchi R, NIshimura K, Tankenaka M, Nishimoto K. Two cases of sporotrichosis (pediatric and adult). Vol. 77, Nishi Nihon Hifuka. 2015. p. 138–41.

2. Fukuda T. Skin care -From infants to the elderly-] fungal infection and skin care. Derma. 2013. p. 62–7.

3. Hata Y. Diagnostic procedures for deep-seated dermatomycosis. Derma. 2011 May;179(55–60):2011.05.

4. Hata Y. [Children’s Skin Diseases -Diagnosis and Treatment-] Fungal infections. Derma. 2018. p. 41–6.

5. Hirruma M. The professionalism of dermatomycosis treatment: Practice of oral antifungal therapy for dermatomycosis. Vol. 124, Nihon Hifuka Gakkai Zasshi ( The Japanese Journal of Dermatology). 2014. p. 2701–2.

6. Iozumi K. Do not overlook granulomatous disease infectious granulomas, bacterial, fungal. Vol. 122, Nihon Hifuka Gakkai Zasshi ( The Japanese Journal of Dermatology). 2012. p. 3483–5.

7. Iwasawa M. Understanding the pathogenesis and clinical presentation of granulomatous diseases bacterial/fungal granulomas. Vol. 124, Nihon Hifuka Gakkai Zasshi ( The Japanese Journal of Dermatology). 2014. p. 3099–101.

8. Kato T. Current status and prospects of deep mycosis of the skin. Vol. 120, Nihon Hifuka Gakkai Zasshi ( The Japanese Journal of Dermatology). 2010. p. 2618–20.

9. Kato T. Skin infections common in children ：Tinea pedis, candidiasis, sporotrichosis. Derma. 2010 Apr;164:97–102.

10. Kon Y, Kimura U, Takeuchi K, Kinosita A, Hiruma M, Takamori K, et al. A case of sporotrichosis (fixed type) on the left cheek treated with local hyperthermia. Vol. 75, Nishi Nihon Hifuka. 2013. p. 432–7.

11. Kurihara Y, Matsumoto N, Hata Y, Doki T, Miyakawa S. A case of sporotrichosis with white colonies arising from a rat bite. Vol. 31, Nihon Rinho Hifuka Ikai Zasshi (Journal of the Japan Organization of Clinical Dermatologists). 2014. p. 477–80.

12. Kusuhara M. Skin diseases common in Kyushu Region sporotrichosis and chromomycosis in Kyushu and Okinawa Region. Vol. 29, Nihon Rinho Hifuka Ikai Zasshi (Journal of the Japan Organization of Clinical Dermatologists). 2012. p. 773–7.

13. Kusuhara M. The Craftsmanship of dermatomycosis practice don’t forget to culture of cutaneous fungal infections. Vol. 124, NIhon Rinho Hifuka Ikai Zasshi (Journal of the Japan Organization of Clinical Dermatologists). 2014. p. 2694–7.

14. Kusuhara M. Current status of deep-seated cutaneous mycosis. Derma. 2018. p. 64–71.

15. Kusunoki T. Shame on the dermatologist for missing it. Atypical cutaneous dermatomycosis. Vol. 29, NIhon Rinho Hifuka Ikai Zasshi (Journal of the Japan Organization of Clinical Dermatologists). 2012. p. 384–8.

16. Makino K, Ogata A, Kudo E, In H, Kamei K. 4 cases of sporotrichosis. Vol. 129, Nihon Hifuka Gakkai Zasshi ( The Japanese Journal of Dermatology). 2019. p. 1497–504.

17. Mochizuki T. Responding to dermatomycosis due to global warming and globalization. Vol. 29, NIhon Rinho Hifuka Ikai Zasshi (Journal of the Japan Organization of Clinical Dermatologists). 2012. p. 79–81.

18. Mochizuki T. My treatment sporotrichosis by experts to reorganize and update treatment methods. Nihon Iji Shinpo (Japan Medical Journal). 2020. p. 45–6.

19. Mochizuki T, Anzai K. Dermatomycosis now and in the future deep-seated dermatomycosis up-to-date. Vol. 36, NIhon Rinho Hifuka Ikai Zasshi (Journal of the Japan Organization of Clinical Dermatologists). 2019. p. 480–4.

20. Mochizuki T, Tsuboi Y, Igarashi K, Ishizaki J, Ushigami T, Ogawa H, et al. Japanese Society of Dermatology Guidelines for the treatment of dermatomycosis 2019. Vol. 129, Nihon Hifuka Gakkai Zasshi (The Japanese Journal of Dermatology). 2019. p. 2639–73.

21. Naka W. Educational Series Superficial mycosis Sporotrichosis. Vol. 53, Medical Mycology Journal. 2012. p. 163–7.

22. Ogawa A, Yoshizaki A, Kuwatsuka Y, Takenaka M, Sato S, Nishimoto K. Two cases of sporotrichosis: A Review of treatment effectiveness of cases in Nagasaki area. Vol. 52, Hifuka No Rinsho (Rinsho derma). 2010. p. 947–51.

23. Ono M, Oda Y, Ishida N, Koba S, Ryo T, Misago N, et al. A case of fixed sporotrichosis of the left upper arm. Vol. 74, Nishi Nihon Hifuka. 2012. p. 170–3.

24. Sakamoto T, Ushigami T, Anzai K, Fujii T, Abe S, Mochizuki T, et al. A case of sporotrichosis rapidly diagnosed by Direct PCR. Vol. 9, Hifu No Kagaku (Skin Research). 2010. p. 254–9.

25. Sato T. Practical management of deep cutaneous fungal infections. Vol. 58, Medical Mycology Journal. 2017. p. E71–7.

26. Shimizu T, Akita S, Harada Y, Oguro E, Okita Y, Shigesaka M, et al. Sporotrichal Tenosynovitis Diagnosed Helpfully by Musculoskeletal Ultrasonography. Vol. 56, Internal Medicine. 2017. p. 1243–6.

27. Suzuki R, Yikelamu A, Tanaka R, Igawa K, Yokozeki H, Yaguchi T. Studies in Phylogeny, Development of Rapid Identification Methods, Antifungal Susceptibility, and Growth Rates of Clinical Strains of Sporothrix schenckii Complex in Japan. Med Mycol J. 2016;57(3):E47-57.

28. Tsubomitsu T, Sawada M, Dekio G, Ninomiya A, Ishizaki J, Tanaka M, et al. A case of lymphangitic sporotrichosis in which the mechanism of spontaneous resolution was considered during the course of the disease. Vol. 35, Nihon Rinho Hifuka Ikai Zasshi (Journal of the Japan Organization of Clinical Dermatologists). 2018. p. 508–13.

29. Ushigami T, Mochizuki T. [Essential fungal disease diagnostics for the dermatologist].How to handle this specimen? Derma. 2011. p. 29–34.

30. Wakumoto K, Yamamoto O. [All About Granulomas] Mycosis. Derma. 2013. p. 53–7.

31. Watanabe S. Infectious Disease Syndromes (2nd ed.)-Including syndromes to infectious mono-diseases-[1]] Fungal infections Sporotrichosis. Nihon Rinsho (Japanese Journal of Clinical Medicine). 2013. p. 609–11.

32. Watanabe S. [All of the Guidelines for Infectious Diseases] Other major academic societies Japanese Dermatological Association and the Japanese Society of Medical Mycology Guidelines for the diagnosis and treatment of skin mycosis. Vol. 32, Kagaku Ryouhou No Ryoiki (Antibiotics & chemotherapy). 2016. p. 854–61.

33. Yamaguchi T, Ito S, Takano Y, Umeda N, Goto M, Horikoshi M, et al. A Case of Disseminated Sporotrichosis Treated with Prednisolone, Immunosuppressants, and Tocilizumab under the Diagnosis of Rheumatoid Arthritis. Vol. 51, Internal Medicine. 2012. p. 2035–9.

34. Yoshizaki A, Yoshizaki A, Kuwatsuka Y, Takenaka M, Nishimoto K, Utani A. A case of sporotrichosis of the neck in a child. Vol. 30, Nihon Shouni Hifuka Gakkai Zasshi (Journal of Pediatric Dermatology). 2011. p. 55–7.

*Mycetoma (5)*

1. Ichinomiya A, Nishimura K, Takenaka M, Utani A, Nishimoto K. Mycetoma caused by Nocardia transvalensis with repeated local recurrences for 25 years without dissemination to viscera. J Dermatol. 2014 Jun;41(6):556–7.

2. Sato T. Practical management of deep cutaneous fungal infections. Vol. 58, Medical Mycology Journal. 2017. p. E71–7.

3. Suzuki C, Shimoyama A, Itsunoi T, Sei Y. Diseases of fascia and subcutaneous tissue] Clinical case: Primary cutaneous nocardiosis (mycetoma type) on the buttocks. Vol. 38, Hifubyo Shinryou (Practical Dermatology). 2016. p. 29–32.

4. Yotsu R. [Recent Topics 2018] Recently discussed skin diseases Skin NTDs (neglected tropical diseases) Perspectives and Japan’s response. Vol. 72, Rinsho Hifuka (Japanese Journal of Clinical Dermatology). 2018. p. 31–6.

5. Yotsu R. New dermatology seminarium - Skin infectious diseases to be aware of zoonosis and tropical skin diseases. Vol. 130, Nihon Hifuka Gakkai Zasshi (The Japanese Journal of Dermatology). 2020. p. 2355–60.

**Onchocerciasis (13)**

1. Fukuda M, Otsuka Y, Uni S, Bain O, Takaoka H. Genetic evidence for the presence of two species of *Onchocerca* from the wild boar in Japan. Parasite. 2010 Mar;17(1):33–7.

2. Fukuda M, Otsuka Y, Uni S, Boda T, Daisaku H, Hasegawa H, et al. Zoonotic onchocerciasis in Hiroshima, Japan, and molecular analysis of a paraffin section of the agent for a reliable identification. Parasite. 2011 May;18(2):185–8.

3. Fukuda M, Uni S, Igari T, Utsumi Y, Otsuka Y, Nakatani J, et al. Human case of *Onchocerca dewittei japonica* infection in Fukushima, Northeastern Honshu, Japan. Parasitol Int. 2019 Oct;72:101943.

4. Fukuda M, Uni S, Otsuka Y, Eshita Y, Nakatani J, Ihara K, et al. A new case of zoonotic onchocercosis in northern Kyushu, Japan. Parasitol Int. 2015 Dec;64(6):519–21.

5. Takaoka H, Fukuda M, Otsuka Y, Aoki C, Uni S, Bain O. Blackfly vectors of zoonotic onchocerciasis in Japan. Med Vet Entomol. 2012 Dec;26(4):372–8.

6. Takaoka H. The *Simuliidae* (Diptera) in the Oriental Region: Classification, morphological characters, geographical distributions, and transmission of filariae. Medical Entomology and Zoology. 2015;66(4):143–65.

7. Takaoka H. Zoonotic onchocerciasis in Iapan: its causative *Onchocerca* species and vector black fly species. Medical Entomology and Zoology. 2015;66(2):23–30.

8. Uni S, Boda T, Daisaku K, Ikura Y, Maruyama H, Hasegawa H, et al. Zoonotic filariasis caused by *Onchocerca dewittei japonica* in a resident of Hiroshima Prefecture, Honshu, Japan. Parasitol Int. 2010 Sep;59(3):477–80.

9. Uni S, Fukuda M, Agatsuma T, Bain O, Otsuka Y, Nakatani J, et al. *Onchocerca takaokai* n. sp. (Nematoda: Filarioidea) in Japanese wild boars (*Susscrofa leucomystax*): Description and molecular identification of intradermal females. Parasitology International. 2015;64(6):493–502.

10. Uni S, Fukuda M, Ogawa K, Lim Yal, Agatsuma T, Bunchom N, et al. Zoonotic infection with *Onchocerca dewittei japonica* in an 11-year-old boy in Kansai Region, Western Honshu, Japan. Parasitol Int. 2017 Oct;66(5):593–5.

11. Uni S, Fukuda M, Otsuka Y, Hiramatsu N, Yokobayashi K, Takahashi H, et al. New zoonotic cases of *Onchocerca dewittei japonica* (Nematoda: Onchocercidae) in Honshu, Japan. Parasit Vectors. 2015 Jan 27;8:59.

12. Yotsu R. Skin NTDs (neglected tropical diseases): Perspectives and Japanese response (Japanese). Rinsho Hihuka (Japanese Journal of Clinical Dermatology). 2018;72(5):31–6.

13. Yotsu R. Zoonosis and Tropical Skin Diseases (Japanese). Nihon Hifuka Gakkai Zasshi (The Japanese Journal of Dermatology). 2020;130(11):2355–60.

**Rabies (21)**

1. Hidano A, Hayama Y, Tsutsui T. Prevalence of immunity presumed using rabies vaccination history and household factors associated with vaccination status among domestic dogs in Japan. Jpn J Infect Dis. 2012;65(5):396–402.

2. Inoue S. Rabies Guideline in Japan 2013 (Japanese). Vol. 67, Jyuui Chikusan Shinpou. 2014. p. 171–5.

3. Kadowaki H, Hampson K, Tojinbara K, Yamada A, Makita K. The risk of rabies spread in Japan: a mathematical modelling assessment. Epidemiol Infect. 2018 Jul;146(10):1245–52.

4. Kashino W, Piyaphanee W, Kittitrakul C, Tangpukdee N, Sibunruang S, Lawpoolsri S, et al. Incidence of potential rabies exposure among Japanese expatriates and travelers in Thailand. J Travel Med. 2014 Aug;21(4):240–7.

5. Kawase K, Yamaguchi S, Sano F, Konno T, Kanejima T, Tamaki, et al. A case treated with post-exposure rabies vaccination after the exposure by a wild megabat. Vol. 68, Jyuui Chikusan Shinpou (Journal of Veterinary Medicine). 2015. p. 276–8.

6. Kuroda Yuhji. Importance of Vaccines from the Viewpoint of Imported Infectious Diseases About Rabies (Japanese). Nihonijishinpo (Japan Medical Journal). 2016;(4789):27–33.

7. Kurosawa A, Tojinbara K, Kadowaki H, Hampson K, Yamada A, Makita K. The rise and fall of rabies in Japan: A quantitative history of rabies epidemics in Osaka Prefecture, 1914-1933. PLoS Negl Trop Dis. 2017 Mar;11(3):e0005435.

8. Kwan NCL, Inoue M, Yamada A, Sugiura K. Evaluating the contact rate between companion dogs during dog walking and the practices towards potential cases of rabies among dog owners in Japan. Zoonoses Public Health. 2019 Jun;66(4):393–400.

9. Kwan NCL, Ogawa H, Yamada A, Sugiura K. Quantitative risk assessment of the introduction of rabies into Japan through the illegal landing of dogs from Russian fishing boats in the ports of Hokkaido, Japan. Prev Vet Med. 2016 Jun 1;128:112–23.

10. Kwan NCL, Yamada A, Sugiura K. Benefit-cost analysis of the policy of mandatory annual rabies vaccination of domestic dogs in rabies-free Japan. PLoS One. 2018;13(12):e0206717.

11. Makita K, Kadoaki Y, Tojinbara K, Yamada A. Assessment of rabies risk in Japan. Vol. 69, Jyuichikusanshinpo (Journal of Veterinary Medicine). 2016. p. 247–51.

12. Malerczyk C, Detora L, Gniel D. Imported human rabies cases in europe, the United States, and Japan, 1990 to 2010. J Travel Med. 2011 Dec;18(6):402–7.

13. Matsumoto N, Ida M. Research on rabies virus antibody level in dogs (Japanese). Tottori ken eisei Kankyo kenkyushohou. 2010. p. 5–7.

14. Miyazu M, Kikuchi H, Goto Y, Yamamoto E. Rabies Postexposure prophylaxis (Japanese). Vol. 7, Nihon Tokouigakkaishi (Journal of Japanese Society of travel and Health). 2014. p. 35–9.

15. Nosaki Y, Maeda K, Watanabe M, Yokoi T, Iwai K, Noguchi A, et al. Fourth imported rabies case since the eradication of rabies in Japan in 1957. J Travel Med. 2021 Sep 20;taab151.

16. Ozawa Y. Review of Wildlife Rabies in the World and Actions to be Taken in Japan. Vol. 17, Jyuiekigakuzashi (The Journal of Veterinary Epidemiology). 2013. p. 132–7.

17. Saeki J, Yamamoto S, Yabe M, Nagasaki J, Hayashi K. Survey of rabies antibody prevalence in young dogs younger than 91 days (Japanese). Vol. 68, Nihon Jyuuisikai zassi. 2015. p. 135–40.

18. Takahashi-Omoe H, Omoe K, Okabe N. Regulatory systems for prevention and control of rabies, Japan. Emerg Infect Dis. 2008 Sep;14(9):1368–74.

19. Takayama N, Suganuma A, Yanagisawa N, Nakayama E. Trends of patients seeking post-exposure prophylaxis at our hospital: exposure and consultations abroad (Japanese). Vol. 143, Nihon Ishikai Zashi. 2014. p. 1529–33.

20. Watanabe I, Yamada K, Aso A, Suda O, Matsumoto T, Yahiro T, et al. Relationship between virus-neutralizing antibody levels and the number of rabies vaccinations: a prospective study of dogs in Japan. Jpn J Infect Dis. 2013;66(1):17–21.

21. Yamada A, Makita K, Kadowaki H, Ito N, Sugiyama M, Kwan NCL, et al. A Comparative Review of Prevention of Rabies Incursion between Japan and Other Rabies-Free Countries or Regions. Jpn J Infect Dis. 2019 Jul 24;72(4):203–10.

**Scabies (9)**

1. Amada D. A case of patient with autism spectrum disorder and crusted (Norweigian) scabies (Japanese). Vol. 37, Nihon Primary case Rengou Gakkaishi. 2014. p. 340–1.

2. Aragaki A, Shirono M. Review of the measures against outbreaks of scabies in the psychiatric ward in Japan (Japanese). Vol. 61, Nihon Seishinka Kango Gakujyutusyuukaishi. 2020. p. 17–21.

3. Executive Committee of Guideline for the Diagnosis and Treatment of Scabies. Guideline for the diagnosis and treatment of scabies in Japan (third edition): Executive Committee of Guideline for the Diagnosis and Treatment of Scabies. J Dermatol. 2017 Sep;44(9):991–1014.

4. Hattori M, Kanazawa N. Two epidemic outbreaks of scabies (Japanese). Vol. 33, Nihon Rinsho Hifukaikai Zasshi. 2016. p. 383–8.

5. Ichinomia A, Nishimoto K. Case of scabis at Nagasaki Ekisaikai Hospital during the two years from September 2013 to August 2015 (Japanese). Vol. 16, Nagasaki-ekisaikai-byouinkaihou. 2015. p. 1–5.

6. Kasamatsu Y, Shirano M, Goto S. A case of AIDS with exacerbation of generalized scabies throughout the body by IRIS (Japanese). Vol. 25, Clinical Parasitology. 2014. p. 68–70.

7. Mashima E, Haruyama S, Yoshioka H, Yamada Y, Nakamura M. Case report of scabies among five siblings treated as intractable eczema (Japanese). Vol. 35, Nihon Shounihifuka gakkaizassi. 2016. p. 51–5.

8. Shiroyama R, Igarashi R, Honda Y, Yoshida T, Oshida K, Higuchi S, et al. A Case of Infantile Scabies Difficult to Differentiate from Multisystem Langerhans Cell Histiocytosis (Japanese). Vol. 122, Nihon Shounika Gakkai Zasshi (The journal of the Japan Pediatric Society). 2018. p. 760–6.

9. Takai T, Kawata Y, Nakamura K, Murata Y. Scabies Infestation within the Horny Layer of a Facial Hyperkeratotic Nodule (Japanese). Vol. 124, Nihon Hifuka Gakkaizasshi. 2014. p. 2299–303.

**Schistosomiasis (43)**

1. Domen K, Tanaka H, Haruno M, Ikeda N, Soujima S. A case of cirrhosis due to schistosomiasis japonica with nonalcoholic fatty liver. Vol. 91, The Japanese Journal of Clinical and Experimental Medicine. 2014. p. 964–6.

2. Ema A, Imai S, Nakagawa M, Kumamoto Y, Nagase T, Kanai T. A case of acute appendicitis with ova of Schistosomia Japonicum observed in the resected specimen. Vol. 72, The journal of the Japan Surgical Association. 2011. p. 1773–7.

3. Hasegawa Y, Nishiii M, Masui S, Yoshio Y, Kanda H, Kanai M, et al. Urinary schistosomiasis: report of a case. Hinyokika Kiyo. 2014 Feb;60(2):91–4.

4. Hoshina K, Hosaka A, Matsumoto J. A surgical case of multiple colon cancers and adenomas associated with Japanese Schistosomiasis. Vol. 43, The Japanese Journal of Gastroenterological Surgery. 2010. p. 673–7.

5. Hoshina S, Yoshikawa K, Kirinoki M, Chigusa Y, Kanuka H, Hori S. A Japanese HIV case with suspected Mekong schistosome infection. Vol. 29, Clinical Parasitology. 2018. p. 53–5.

6. Hosho K, Ikebuchi Y, Ueki M, Nakamura K, Yashima K, Maeda N, et al. Schistosoma Japonica identified by laparoscopic and colonoscopic examination. Vol. 22, Digestive Endoscopy. 2010. p. 133–6.

7. Imai J, Ichikawa H, Mizukami H, Suzuki T, Watanabe N, Mine T, et al. Colonic High-grade Tubular Adenomas Associated with Schistosoma japonicum. Vol. 41, The Tokai Journal of Experimental and Clinical Medicine. 2016. p. 22–3.

8. Ishihara R, Hashimoto A, Saiki R, Ikenoyama Y, Tahara Y. A case of Schistosomiasis japonica with a history of living in the Philippines. Vol. 26, Clinical Parasitology. 2015. p. 52–4.

9. Iwata T, Kondo Y, Kimura O, Fujishima F, Morosawa T, Ninomiya M, et al. A case of previous infection with schistosomiasis japonica diagnosed holistically on the basis of various clinical examination findings. Nihon Shokakibyo Gakkai Zasshi. 2014 May;111(5):948–55.

10. Kajihara N, Hirayama K. The war against a regional disease in Japan: A history of the eradication of Schistosomiasis japonica. Vol. 39, Tropical Medicine and Health. 2011. p. 3–44.

11. Kano K, Tamagawa H, Sawazaki S, Yukawa N, Rino Y, Masuda M. A case of gangrenous appendicitis with ova of Schistosoma japonicum observed in the resected specimen. Vol. 76, Journal of Japan Surgical Association. 2015. p. 545–9.

12. Kato T, Kawakami T, Fukazawa K. A case of epididymal granuloma caused by schistosomiasis. Vol. 24, Japanese Journal of Urological Surgery. 2011. p. 544.

13. Kawai S, Kano M, Suzuki M, Hirano T, Kirinoki M, Chigusa Y. A case of Schistosoma japonicum and egg nodule detected in a polyp removed from the ascending colon in the general care unit. Vol. 26, Journal of Generalist Medicine. 2016. p. 968–9.

14. Kirinoki M, Hayashi N, Chigusa Y. Schistosomiasis. Vol. 42, Dokkyo Journal of Medical Sciences. 2015. p. 233–7.

15. Koizumi Y, Hirooka M, Tanaka T, Sunago K, Yukimoto A, Imai Y, et al. Schistosomiasis diagnosed using laparoscopy and colonoscopy. Vol. 58, Internal Medicine. 2019. p. 2495–9.

16. Koyama T, Sakai K, Taniguchi H, Murakami M, Ikeda K, Nakamichi I, et al. A case with old ova of Schistosoma Japonicum that presented with gangrenous appendicitis. Vol. 77, Journal of Japan Surgical Association. 2016. p. 2733–7.

17. Kumagaya Y, Tuboi K, Tanishima Y, Yoshimoto K, Kajimoto T, Kashiwagi H. Two cases of gastric cancer resection with schistosomiasis japonica. Vol. 72, Journal of Clinical Surgery. 2017. p. 86–90.

18. Machida K, Yamada T, Shimura E, Umemura M, Onoue S. Schistosomiasis japonica in a patient who emigrated from China: a case report. Vol. 115, Nihon Shokakibyo Gakkai Zasshi. 2018. p. 1094–100.

19. Makino Y, Aoki J, Okazawa Y, Takahashi R, Mizukoshi K, Kawai M, et al. A case of colon adenoma associated with Schistosomiasis japonica. Vol. 83, Progress of Digestive Endoscopy. 2013. p. 158-159,13.

20. Minai M. Oncomelania nosophora in Kofu basin,Yamanashi Prefecture, Japan. Vol. 63, Medical Entomology and Zoology. 2012. p. 257–62.

21. Miyazawa M, Miyaki A, Nishiguchi R, Kono T, Otani T, Takaoka K, et al. A case of gangrenous appendicitis with ova of Schistosoma Japonicum detected in the resected specimen  Kazuhiko Takaoka１）, Kenichi Kumazawa１）, Ryota Matsuo１） and Yoshihiko Naritaka２）. Vol. 38, Journal of Japanese College of Surgeons. 2013. p. 842–5.

22. Mizuno Y, Hayashi N, Chigusa Y. A case of bilharziasis with calcification of the seminal vesicle. Vol. 26, Clinical Parasitology. 2015. p. 49–51.

23. Mori T, Matsumoto S, Imoto Y, Shinomiya H, Wada S. Scistosomiasis Japonicum found in an area not endemic. Vol. 55, Acta hepatologica Japonica. 2014. p. 254–8.

24. Murayama S, Nomura S, Kaneko M, Honma Y, Takemura T, Tomita K. Infection with schistosomal parasites in Africa. Vol. 66, Japanese journal of clinical urology. 2012. p. 319–22.

25. Muto Y, Nabeya Y, Tanizawa Y, Hayashi H, Tanizawa T, Ochiai T, et al. A case of multiple early gastric carcinomas at five sites accompanied by old Schistosoma Japonicum infection. Vol. 71, Journal of Japan Surgical Association. 2010. p. 2021–5.

26. Nihei N. Focused on distribution and monitoring of Oncomelania nosophora in Japan. Vol. 63, Medical Entomology and Zoology. 2012. p. 249–56.

27. Nihei N, Kirinoki M, Koen H, Tsuyuguchi T, Saito Y, Taira kensuke, et al. Changes in the habitat of Oncomelania hupensis nosophora, the intermediate host snail of Schistosoma japonicum, along the Obitsu River basin, Chiba Prefecture, Japan. Vol. 69, Medical Entomology and Zoology. 2018. p. 19–29.

28. Ohata T, Nagasako Y, Sunahara M, Ogasawara K, Kusano M. A case of rectal cancer presenting with Schistosoma Japonicum eggs in an area not endemic for Schistosomiasis Japonica. Vol. 74, Journal of Japan Surgical Association. 2013. p. 735–8.

29. Okumura S, Imagawa A, Kawashima H, Toyoda S, Yasuda H, Yamamoto K, et al. A case of rectal cancer presenting with schistosomiasis japonica eggs. Gan To Kagaku Ryoho. 2017 Nov;44(12):1341–2.

30. Oya H, Koshikawa K, Sanada S, Uno Y, Sano M, Fukuoka T. Rare case of appendicitis caused by the eggs of Schistosoma japonicum treated with laparoscopic surgery. Vol. 77, Journal of Japan Surgical Association. 2016. p. 373–7.

31. Saiki Y, Iwamoto K, Tanaka M, Yamada K. A Case of rectal cancer with ova of Schistosoma Japonicum. Vol. 80, Journal of Japan Surgical Association. 2019. p. 1871–6.

32. Sakuyama N, Kobayashi A, Kojima M, Ikeda K, Matsunaga R, Kawano S, et al. A case of sigmoid colon cancer and postoperative liver metastasis with Schistosoma japonicum eggs Masaaki Ito1) and Norio Saito. Vol. 69, Journal of the Japan Society of Coloproctology. 2016. p. 170–5.

33. Shimada M, Shibahara K, Kitamura H, Moriyama H, Hada M, Takehara A, et al. A case of necrotizing ischemic colitis suspected being induced by schistosomal ova embolism. Vol. 43, The Japanese Journal of Gastroenterological Surgery. 2010. p. 572–7.

34. Takahashi K, Aoki C, Nagashima K, Moriyama A, Taniguchi T, Oji A, et al. A case of idiopathic thrombocytopenic purpura suspected during pregnancy and diagnosed as schistosomiasis japonica after delivery. Vol. 59, Tokyo Journal of Obstetrics and Gynecology. 2010. p. 378–83.

35. Tsubouchi K, Tsubouchi H, Yokoyama H, Irie S, Yoshida K, Tanaka M. Retroperitoneal fibrosis due to Schistosoma japonicum: a case report. Nihon Hinyokika Gakkai Zasshi. 2010 Jul;101(5):694–7.

36. Tsukagoshi H, Tsutsumi H, Iwazaki S, Kurabayashi M, Takeyoshi I. Numerous old Schistosoma Japonicum eggs found in a resected specimen in a case of perforated colonic diverticulum. Vol. 62, The Kitakanto Medical Journal. 2012. p. 153–7.

37. Utagawa M, Gen T, Yamaura C, Akao N. A case of transverse colon carcinoma in which Schistosoma Japonicum eggs were found in the excised specimen, leading to liver failure after a rapid course. Vol. 21, Clinical Parasitology. 2011. p. 40–2.

38. Washino T, Shida D, Tanizawa T, Nasu K, Miyamoto S, Inoue S. Synchronous multiple colon cancers associated with Japanese Schistosomiasis―Report of a case. Vol. 73, Journal of Japan Surgical Association. 2012. p. 2621–5.

39. Yamaguchi A, Kimura S, Anbai M, Shirokura T, Tanaka K, Yamaguchi S, et al. A case of amoebiasis and giardiasis complicated by old Japanese Schistosomiasis. Vol. 21, Clinical Parasitology. 2011. p. 46–9.

40. Yamauchi M, Fukuyama T, Kurata K, Nagao Y, Egami T, Nakashima H, et al. A case of ascending colon cancer associated with Japanese Schistosomiasis. Vol. 41, Journal of Japanese College of Surgeons. 2016. p. 971–4.

41. Yanagisawa S, Yuasa T, Tanaka T. Clinical Diagnosis of Schistosoma japonicum Infection Complicating Infective Endocarditis and Liver Cirrhosis. Vol. 49, Internal Medicine. 2010. p. 1001–5.

42. Yokoyama H, Koyama T, Komiyama S, Serizawa M. Detection of Schistosoma Japonicum eggs in tissues over the past 50 years-A review of 1961-2010. Vol. 39, Yamanashi Igaku. 2011. p. 15–20.

43. Yokoyama H, Koyama T, Komiyama S, Serizawa M. Detection of Schistosoma japonicum eggs in tissues in recent years (2013-2015). Vol. 44, Yamanashi Igaku. 2017. p. 81–4.

**Snakebites (75)**

1. Abe M, Kume H. Current status and prospects of infection: skin infection. Vol. 42, Nihon Bokin Bobai Gakkaishi (Journal of Antibacterial and Antifungal Agents). 2014. p. 361–7.

2. Arai H, Nishino T, Kono S. Kidney disorder by animal-borne poison: mamushi bites and bee sting. Vol. Suppl, Nihon Rinsho (Japanese Journal of Clinical Medicine). 2012. p. 728–30.

3. Chiba T, Koga H, Kimura N, Murata M, Jinnai S, Suenaga A, et al. Clinical condition and management of 114 mamushi (Gloydius blomhoffii) bites in a general hospital in Japan. Intern Med. 2018 Apr 15;57(8):1075–80.

4. Fujii K, Harada D, Kujurai D, Kase K, Miyatake S. A case of a child bitten by mamushi and treated with antivenom. Vol. 38, Nihon Kyukyu Igakukai Kanto Chihokai Zasshi (KANTO Journal of Japanese Association for Acute Medicine). 2017. p. 322–4.

5. Fujiyama Y, Shirako T, Yoshino K, Sano A, Yamada S, Ikawa A, et al. Twenty five cases of mamushi bite in our hospital. Vol. 7, Nihon Kyukyu Igakukai Chubu Chihokaishi (Chuubu Journal of Acute Medicine). 2011. p. 19–22.

6. Groneberg DA, Geier V, Klingelhöfer D, Gerber A, Kuch U, Kloft B. Snakebite envenoming - a combined density equalizing mapping and scientometric analysis of the publication history. PLoS Negl Trop Dis. 2016 Nov;10(11):e0005046.

7. Hara J. Clarifying the basis for referral: snakebites by habu and mamushi. jmed mook. 2015. p. 118–22.

8. Hatanaka W. A case of mamushi viper bite in a hand of a child. Vol. 31, Nihon Syugekagakkai Zasshi (The journal of Japanese Society for Surgery of the Hand). 2014. p. 338–41.

9. Hatanaka W. Anti-toxin and relaxation incision treatment after mamushi viper bite. Vol. 23, Nihon Shoni Seikeigeka Gakkai Zasshi (J Jpn Ped Orthop Ass). 2014. p. 285–8.

10. Hifumi T. Pathology and clinical examination of snakebite: diagnosis. Vol. 7, Thrombosis Medicine. 2017. p. 131–5.

11. Hifumi T. Pathology and clinical examination of snakebite: treatment. Vol. 7, Thrombosis Medicine. 2017. p. 217–9.

12. Hifumi T. Clinical serum therapy. Vol. 25, Nihon Syuchu Chiryo Igakukai Zasshi (Journal of the Japanese Society of Intensive Care Medicine). 2018. p. 235–42.

13. Hifumi T, Murakawa M, Sakai A, Ginnaga A, Yamamoto A, Ato M, et al. Potentially fatal coagulopathy secondary to yamakagashi (Rhabdophis tigrinus) bites that completely recovered with antivenom treatment. Acute Med Surg. 2014 Aug 8;2(2):123–6.

14. Hifumi T, Sakai A, Kondo Y, Yamamoto A, Morine N, Ato M, et al. Venomous snake bites: clinical diagnosis and treatment. Journal of Intensive Care. 2015 Apr 1;3(1):16.

15. Hifumi T, Sakai A, Yamamoto A, Murakawa M, Ato M, Shibayama K, et al. Clinical characteristics of yamakagashi (Rhabdophis tigrinus) bites: a national survey in Japan, 2000-2013. J Intensive Care. 2014;2(1):19.

16. Hifumi T, Sakai A, Yamamoto A, Murakawa M, Ato M, Shibayama K, et al. Effect of antivenom therapy of Rhabdophis tigrinus (Yamakagashi snake) bites. J Intensive Care. 2014;2(1):44.

17. Hifumi T, Yamamoto A, Morokuma K, Ogasawara T, Kiriu N, Hasegawa E, et al. Surveillance of the clinical use of mamushi (Gloydius blomhoffii) antivenom in tertiary care centers in Japan. Jpn J Infect Dis. 2011;64(5):373–6.

18. Hifumi T, Yamamoto A, Morokuma K, Okada I, Kiriu N, Ogasawara T, et al. Clinical efficacy of antivenom and cepharanthine for the treatment of Mamushi (Gloydius blomhoffii) bites in tertiary care centers in Japan. Jpn J Infect Dis. 2013;66(1):26–31.

19. Iguchi A, Kitazawa K, Nishimura T, Tanese S, Toube Y, Kobayashi H, et al. Eight pediatric cases of mamushi bite with administration of antivenom. Vol. 17, Nihon Shouni Kyukyu Igakukai Zasshi (Journal of Japanese Society of Emergency Pediatrics). 2018. p. 401–7.

20. Ikegami F. Folk medicines in Japan: Sanpenzu. Vol. 61, Wakanyaku. 2011. p. 7–8.

21. Ikemura K, Yoshioka N, Nagayama Y. A case of mamushi bite. Vol. 34, Nihon Keiseigeka Gakkai Kaishi (Journal of Japan Society of Plastic and Reconstructive Surgery). 2014. p. 766–70.

22. Imamura T, Takemoto M, Kouno H, Ito T. Case report: a case of edema of the right upper extremity after a Mamushi bite that was treated using Goreisan. Vol. 64, Bouei Eisei (National Defense Medical Journal). 2017. p. 275–9.

23. Ishikawa H. Emergency manual for dermatologists: mamushi bites. Derma. 2016. p. 55–8.

24. Ishikawa K, Ohsaka H, Omori K, Obinata M, Mishima K, Oode Y, et al. Pregnant woman bitten by a Japanese mamushi (Gloydius blomhoffii). Intern Med. 2015;54(19):2517–20.

25. Ishikawa T, Soejima O, Nakagawa H, Kitamura T, Naito M. Five cases that developed compartment syndrome in hands and arms after mamushi bites. Vol. 26, Nihon Syugekagakkai Zasshi (The Journal of Japanese Society for Surgery of the Hand). 2010. p. 453–5.

26. Izumi Y, Terada K, Morine N, Kudaka J. Epidemiology of venomous snakebite in Okinawa prefecture in 2014. Okinawaken Eiseikankyo Kenkyuzyoho (Annual Report of Okinawa Prefectural Institute of Health and Environment). 2015. p. 81–3.

27. Jin K, Munakata T, Akita N. Clinical reports of twenty six cases of mamushi bites in our hospital department. Vol. 41, Hifubyo Shinryo (Practical Dermatology). 2019. p. 608–13.

28. Kawamoto K, Miyamoto K, Harada N, Yamashita N. A case of acute renal failure due to viper bite and clinical characteristics of 64 cases over 8 years. Vol. 1, Journal of Japan Society for Blood Purification in Critical Care. 2010. p. 141–5.

29. Kawamura Y, Nagai M, Kuraishi N, Tamura M. A case of cerebral infarction associated with Japanese mamushi (Gloydius blomhoffii blomhoffii) bite. Journal of the Japanese Association of Rural Medicine. 2014;63(1):57–60.

30. Kimura N, Okabe N, Futamura-Nakagawa R, Koda F, Izumo A, Furue M. Clinical study of 81 cases of “mamushi” viper bite during the past 11 years. Nishi Nihon Hifuka. 2015;77(6):584–8.

31. Kinoshita H, Komei N, Yamazoe M, Tsubakibara H, Miyamoto A, Mori K, et al. Considerations on initial treatment of 23 cases of mamushi bites. Vol. 31, Nihon Kyukyu Igakukai Zasshi. 2020. p. 366–71.

32. Kotake M, Suzuki S. Two cases that developed purulent osteomyelitis after mamushi bites. Vol. 35, Nihon Syugekagakkai Zasshi (The journal of Japanese Society for Surgery of the Hand). 2019. p. 1244–8.

33. Kuda T, Yamashiro K, Umetani K, Watanabe S, Tamura K. Study of 65 patients with habu bites in Kumejima, Okinawa Prefecture - special reference to the related symptoms, dry anti-habu equine toxin, first-aid measures and prognosis. An Official Journal of the Japan Primary Care Association. 2018;41(4):143–7.

34. Kushibe I, Kawai H, Takano D. Experience of hand therapy in a case of mamushi bite. Vol. 9, Nihon Hand Therapy Gakkaishi (The Journal of Japanese Hand Therapy Society). 2017. p. 96–100.

35. Kushida N, Wada T, Nangaku M. Kidney disorder observed in different clinical conditions: serum diseases. Vol. Suppl, Nihon Rinsho (Japanese Journal of Clinical Medicine). 2012. p. 761–4.

36. Masui D, Fukahori S, Kurahachi T, Sakamoto S, Higashidate N, Naoki H, et al. A case of focal lesion after a viper bite successfully treated with herbal medicine. Nihon Shouni Kyukyu Igakukai Zasshi (Journal of Japanese Society of Emergency Pediatrics). 2020;19(2):190–4.

37. Matori S, Miyagi T, Hanashiro H, Sonosaki T, Hayashi K, Awasawa T, et al. Early onset of acute compartment syndrome diagnosed by a simple needle manometer technique. Nishi Nihon Hifuka. 2013;76(5):454–8.

38. Matsuda M. Fang marks and edematous swelling in distal portion of the extremities with severe pain: mamushi bites. Derma. 2018. p. 63–6.

39. Miike T, Sakamoto Y. Perfect cure of diseases caused by summer animals - snakebites. Derma. 2018. p. 66–76.

40. Miura S, Oikawa Y, Ueda Y, Murakami M, Sakata N. Cases of mamushi bite patients hospitalized and treated in Noto Region, Ishikawa Prefecture. Vol. 27, Clinical Parasitology. 2016. p. 75–7.

41. Mogi M, Murate W, Sakai R, Suzuki K, Matsunaga K. Clinical study of 22 viper bite cases at our hospital. Hifu No Kagaku. 2017;16(2):113–9.

42. Murata R, Kobayashi N, Watanabe Y, Kawamata T, Echizenya Y. Three cases of mamushi bites. Vol. 6, Otaru Shirtsu Byoinshi (The Journal of Otaru Municipal General Hospital). 2018. p. 73–6.

43. Nakae H. Two cases of viper bite envenomation treated with Saireito. Kampo Medicine. 2013;64(4):216–21.

44. Nakae H, Igarashi T. A case of lymphedema of the arm after viper venom successfully treated with traditional Japanese medicines. Vol. 61, Japanese Society of Occupational Medicine and Traumatology. 2013. p. 204–7.

45. Nakagawa K. What should you do when your patient was bitten by something in mountains? Vol. 13, Resident Note. 2012. p. 2980–5.

46. Nakamura K, Ideno N, Murakami M, Ogawa Y, Momii S. A case of acute renal and respiratory failure due to mamushi bite. Nihon Kyukyu Igakukai Zasshi. 2010;21(10):843–8.

47. Nakamura T. Watch your step! Troublesome insects hidden close to you: ticks, Latrodectus mactans, stink bugs, or mamushi? Vol. 34, Nihon Rinsho Hifukaikai Zasshi (Journal of the Japan Organization of Clinical Dermatologists). 2017. p. 684–7.

48. Okamoto O, Nakashima R, Yamamoto S, Hashimoto T, Takasaki T, Tokuda H, et al. A lethal case of mamushi (Gloydius blomhoffii) bite: severe bowel symptoms as a lethal sign. Acute Med Surg. 2017 Jan;4(1):135–9.

49. Okamoto O, Sato S, Sakai T, Uehara M, Takenaka R, Ito A, et al. Comparative analysis of mamushi (Gloydius blomhoffii) bite patients indicates that creatinine kinase levels/white blood cell count trends reflect severity. Acute Med Surg. 2016 Apr;3(2):120–7.

50. Sakai A. Snakes and snake venom. Vol. 74, Koushueisei (The Journal of Public Health Practice). 2010. p. 377–81.

51. Sakai A. Diagnosis and treatment of snakebite by Mamushi and Yamakagashi. Chudoku Kenkyu. 2013 Sep;26(3):193–9.

52. Sakai J. Adverse effects and poisonous diseases by metal or chemical materials - natural poison - snake venom. Vol. Suppl, Nihon Rinsho (Japanese Journal of Clinical Medicine). 2014. p. 696–9.

53. Sakai J. Snakebite antivenom. Vol. 30, Chudoku Kenkyu (The Japanese Journal of Toxicology). 2017. p. 41–5.

54. Sakai J. Snakebite envenoming. Vol. 43, Kyukyu Igaku (The Japanese Journal of Acute Medicine). 2019. p. 985–90.

55. Sato Y. Four canine cases of snake bites by mamushi (Gloydius blomhoffii). Vol. 19, Zyui Rinsho Hihuka (The Japanese Journal of Veterinary Dermatology). 2013. p. 217–23.

56. Sato Y. Two feline cases of snake bites by mamushi (Gloydius blomhoffii). Vol. 20, Zyui Rinsho Hihuka (The Japanese Journal of Veterinary Dermatology). 2014. p. 85–9.

57. Shimada M, Komatsumoto S, Kirinoki M, Chigusa Y, Matsuoka H. Characteristics of patients with bee sting, centipede bite, or viper bite treated at Ashikaga Red Cross Hospital in Tochigi Prefecture, Japan between 2009 and 2011. Vol. 63, Eisei Dobutsu (Medical Entomology and Zoology). 2012. p. 103–7.

58. Shimamoto T, Miyachi M, Kuwahara Y, Ogawa H, Nishimura A, Imai M. A case of pit viper bite complicated by wound infection. Kyotofuritsu Yosanoumi Byoinshi. 2012;9(1):59–63.

59. Shinohara M, Muguruma T. Manual of clinical examination for pediatric and adolescent emergency cases of animal bites. Vol. 141, Nihon Ishikai Zashi (The Journal of the Japan Medical Association). 2012. p. S351–2.

60. Shirokawa M, Seki K, Nakajima Y, Koyama S, Mitsusada M. Patient without neurotoxic symptom after being bitten by an eastern green Mamba (Dendroaspis angusticeps). 日本救急医学会雑誌. 2011;22(9):777–81.

61. Tajika M. Main points of diagnosis and treatment of snakebites. Thrombosis Medicine. 2014;4(3):286–90.

62. Takahashi K. Clinical lecture: a sligtly different skin disease in Okinawa. Vol. 62, Hifuka no Rinsho. 2020. p. 175–82.

63. Taki K, Ariyoshi K, Sakai A, Ishikawa H, Nakashima K, Endoh Y. Analysis of viper bites by the national survey. Journal of Japanese Society for Emergency Medicine. 2014;17(6):753–60.

64. Tamehiro K, Shima H, Taki K. A survived case of serious viper bite complicated with acute renal failur. Vol. 15, Nihon Rinsho Kyukyu Igakukai Zasshi (Journal of Japanese Society for Emergency Medicine). 2012. p. 546–9.

65. Terashita S, Murakami M, Saito Y, Hashimoto I, Igarashi N, Tsuhata S. Clinical analysis of childhood pit viper bite in Toyama area. Vol. 70, Shonika Rinsho (Japanese Journal of Pediatrics). 2017. p. 1308–11.

66. Terazumi K, Yanagikawa Y, Honda Y, Sakamoto T. Case report: a case of viper (Agkistrodon halys blomhoffii ‘Mamushi’) bite. Vol. 58, Bouei Eisei (National Defense Medical Journal). 2011. p. 37–40.

67. Tomihara Y. Habu. Vol. 8, Nihon Rinsyo Kokiatsu Sanso Sensui Igakukai Zasshi (Journal of Japanese Association for Clinical Hyperbaric Oxygen and Diving). 2011. p. 4–6.

68. Tsubokura Y, Kume N, Kobe N, Yamazoe T, Masuda N, Yamauchi S, et al. A pediatric case of mamushi bite diagnosed five days after the injury. Vol. 36, Nihon Shouni Hifuka Gakkai Zasshi. 2017. p. 65–9.

69. Tsujimoto T, Chishiro T, Kotani Y, Kamei J, Yamada Y. Analysis of 38 Mamushi pit viper bite cases where patients did not receive serum antitoxin. Vol. 28, Journal of Japanese Association for Acute Medicine. 2017. p. 48–54.

70. Uezu Y, Mahoe Y, Terada T, Morine N, Kudaka J. Epidemiology of venomous snakebite in Okinawa Prefecture in 2012. Okinawaken Eiseikankyo Kenkyuzyoho (Annual Report of Okinawa Prefectural Institute of Health and Environment). 2013. p. 79–81.

71. Uezu Y, Terada K, Morine N, Kudaka J. Epidemiology of venomous snakebite in Okinawa Prefecture in 2013. Okinawaken Eiseikankyo Kenkyuzyoho (Annual Report of Okinawa Prefectural Institute of Health and Environment). 2014. p. 75–7.

72. Yasunaga H, Horiguchi H, Kuwabara K, Hashimoto H, Matsuda S. Short report: venomous snake bites in Japan. Am J Trop Med Hyg. 2011 Jan;84(1):135–6.

73. Yokoi H, Sakai A, Kodama T, Magome S, Nagayasu T, Tawara M, et al. Severe hypofibrinogenemia in patients bitten by Gloydius tsushimaensis in Tsushima Island, Nagasaki, Japan, and treatment strategy. Toxicon. 2020 Dec;188:142–9.

74. Yoneda M, Nakamura T, Mori S, Kurai M, Yoshino A, Ito M, et al. Five cases of mamushi bites and their treatment. Nihon Shounikaikai Kaiho (The Journal of the Japan Pediatric Association). 2017. p. 197–201.

75. Yoshimine S, Seyama A, Suga A, Murakami M, Hayashi M, Inoue T, et al. Clinical study of 67 cases of Japanese mamushi viper (Gloydius blomhoffii) bite. Journal of the Japanese Association of Rural Medicine. 2019;68(4):468–74.

**Soil-transmitted helminthiases**

Ascariasis (11)

1. Fujimori S, Muto M, Yamazaki H, Sakamoto N. Two cases of small intestinal parasites encountered by capsule endoscopy. Clinical Parasitology. 2012;23(1):20–2.

2. Hasegawa M, Pilotte N, Kikuchi M, Means AR, Papaiakovou M, Gonzalez AM, et al. What does soil-transmitted helminth elimination look like? Results from a targeted molecular detection survey in Japan. Parasit Vectors. 2020 Jan 8;13(1):6.

3. Hatanaka H, Umemoto H, Arai H, Iide T, Yamada M, Tou N. Parasitic infections in persons from abroad. Clinical Parasitology. 2015;26(1):71–3.

4. Ikuta S, Mizuguchi Y, Tsuji Y, Tsukamoto S, Oba N, Nishinakagawa S, et al. A case of ascariasis in the gallbladder successfully treated with an anthelmintic injection into the gallbladder via a percutaneous transhepatic catheter. Nihon Shokakibyo Gakkai Zasshi. 2010 May;107(5):768–74.

5. Inoue K, Eshita Y, Kobayashi T, Hasegawa H, Murakami K. A case of mixed infection with whipworm, hookworm, and roundworm, discovered by anemia and observed endoscopy. Clinical Parasitology. 2014;25(1):27–9.

6. Matuoka H, Shimada M, Yamamoto D, Hayakawa E. First case of ascariasis in 7 years referred to the Department of Medical and Zoological Sciences. Clinical Parasitology. 2015;26(1):138–40.

7. Ogawa M, Ueno A, Tanabe M, Raina Angeli Abad M, Tsukuda G, Matsuo K, et al. A case of human ascariasis accidentally detected during comprehensive colonoscopic examination. Prog dig Endosc. 2019 Dec 20;95(1):107–8.

8. Sugiyama H, Morishima Y, Kagawa C, Araki J, Iwaki T, Ikuno H, et al. Current Incidence and Contamination Sources of Ascariasis in Japan. Shokuhin Eiseigaku Zasshi. 2020;61(4):103–8.

9. Sugiyama H, Arakawa kyoko, Shibata K, Kawakami Y, Morishima Y, Yamazaki H, et al. Soil-transmitted Helminth Infections in Japan : Current Ascariasis Case Numbers and an Attempt to Identify Infection Sources. Food sanitation research. 2015;65(4):37–41.

10. Suzuki J, Sasahara T, Houzuki S, Okabe T, Oonishi T, Sotojima M, et al. A case of human ascariasis discovered in the Northern Kanto region in 2015. Clinical Parasitology. 2015;26(1):135–7.

11. Umetsu S, Sogo T, Iwasawa K, Kondo T, Tsunoda T, Oikawa-Kawamoto M, et al. Intestinal ascariasis at pediatric emergency room in a developed country. World J Gastroenterol. 2014 Oct 14;20(38):14058–62.

*Trichuriasis (7)*

1. Hasegawa M, Pilotte N, Kikuchi M, Means AR, Papaiakovou M, Gonzalez AM, et al. What does soil-transmitted helminth elimination look like? Results from a targeted molecular detection survey in Japan. Parasit Vectors. 2020 Jan 8;13(1):6.

2. Inoue K, Eshita Y, Kobayashi T, Hasegawa H, Murakami K. A case of mixed infection with whipworm, hookworm, and roundworm, discovered by anemia and observed endoscopy. Clinical Parasitology. 2014;25(1):27–9.

3. Ishii A, Tanaka S, Nakayasu S, Yamada M. A case of Trichiuriasis. Vol. 28, Clinical Parasitology. 2017. p. 42–4.

4. Narasaka T, Suzuki H, Mizokami H, Miura M. A case of whipworm disease complicated by tuberculous peritonitis and detected by colonoscopy. Clinical Parasitology. 2012;23(1):29–31.

5. Seto T, Hikita N, Abe J, Teramoto I, Uni S, Shintaku H. A review of parasitic infections in our pediatric department. Clinical Parasitology. 2012;23(1):112–5.

6. Suzuki, Kohei R, Katukura N, Watanabe T, Wada Y, Akao N, et al. Diagnosis and treatment experiences of gastrointestinal parasitic infections. Clinical Parasitology. 2014;25(1):9–12.

7. Yamada M, Ishida N, Hottuta K, Imamoto E, Kino H, Nakaya T, et al. Five cases of Trichuriasis detected and diagnosed during colonoscopy and species identification by genetic diagnosis using excised parasites. Clinical Parasitology. 2015;26(1):28–31.

*Necatoriasis (5)*

1. Hasegawa M, Pilotte N, Kikuchi M, Means AR, Papaiakovou M, Gonzalez AM, et al. What does soil-transmitted helminth elimination look like? Results from a targeted molecular detection survey in Japan. Parasit Vectors. 2020 Jan 8;13(1):6.

2. Inoue K, Eshita Y, Kobayashi T, Hasegawa H, Murakami K. A case of mixed infection with whipworm, hookworm, and roundworm, discovered by anemia and observed endoscopy. Clinical Parasitology. 2014;25(1):27–9.

3. Minezaki S, Hirama T, Shiono A, Masumoto A, Mio T, Utsugi H, et al. A case of lung paragonimiasis superinfection with hookworm presenting difficulty in discrimination. Kansenshogaku Zasshi. 2013 Nov;87(6):756–60.

4. Mizuno Y, Takeshita N, Kato Y, Morishima Y, Yamazaki H. A case of American necatoriasis resistant to anthelmintic treatment with a single dose of pyrantel pamoate. Vol. 22, Clinical Parasitology. 2011. p. 65–7.

5. Shimada M, Komatumoto satoru, Kirinoki M, Chigusa yuuichi, Matuoka H. A review of cases in parasite and -hygenic animal related disease at a regional core hospital in Tochigi Prefecture. Vol. 34, Jichi Medical University Journal. 2012. p. 141–8.

*Strongyloidiasis* (45)

1. Adachi, Takayuki, Machida, Shinji, Uehara, Keita, Sekiya, Shusuke, Shibagaki, Yugo. A case of strongyloidiasis detected while searching for the cause of eosinophilia in a patient undergoing hemodialysis. Nihon Toseki Igakkai Zasshi. 2015;48(11):657–62.

2. Hasegawa H, Sato H, Fujita S, Nguema PPM, Nobusue K, Miyagi K, et al. Molecular identification of the causative agent of human strongyloidiasis acquired in Tanzania: dispersal and diversity of Strongyloides spp. and their hosts. Parasitol Int. 2010 Sep;59(3):407–13.

3. Higashiarakawa M, Tetsuo H, Teruhisa T, Gretchen P, Tetsu K, Hidekatsu N, et al. Normal Serum IgE Levels and Eosinophil Counts Exhibited during Strongyloides Stercoralis Infection. Parasitology International. 2017;66(1):807–12.

4. Higashishinkawa M, Hirata T, Oshiro M, Ishikawa M, Tanaka T, Kishimoto K. A case of active strongyloides sterocolaris from pancreatic juice. Clinical Parasitology. 2013;24(1):84–6.

5. Hosoda T, Sakamoto M, Orikasa H, Kubomura A, Misaki T, Okabe N. Septic Meningitis and Liver Abscess due to Hypermucoviscous Klebsiella pneumoniae Complicated with Chronic Strongyloidiasis in a Human T-lymphotropic Virus 1 Carrier. Intern Med. 2020 Jan 1;59(1):129–33.

6. Ikuno H, Ishikawa T, Norose K. Status of Strongyloidiasis in Japan, 2000-2017. Am J Trop Med Hyg. 2020 Aug;103(2):727–34.

7. Ishikawa S, Maeda T, Hattori K, Watanabe T, Kuramoto T, Ueno S, et al. A case of adenocarcinoma developed in the small intestine with chronic strongyloidiasis. Clin J Gastroenterol. 2017 Dec;10(6):519–23.

8. Iwashita Y, Suzuki K, Masui A, Kawamoto E, Yokoyama K, Yamamoto A, et al. A case of Strongyloides hyperinfection associated with tuberculosis. Journal of Intensive Care [Internet]. 2013;1(7). Available from: https://doi.org/10.1186/2052-0492-1-7

9. Kameyama S, Isa T, Motonari H, Taniguchi H, Koami H, Saio M. A case of intrahepatic cholangiocarcinoma in situ associated with strongyloidiasis. Journal of Japan Surgical Association. 2012;73(1):116–20.

10. Kanazawa S, Yamaguchi K, Yamagata N, Shimada R, Yoneda K, Sakurai S. A case of Strongyloides infection discovered during treatment for aspiration. Clinical Parasitology. 2014;25(1):16–9.

11. Kikuchi T, Hino A, Tanaka T, Aung MPPTHH, Afrin T, Nagayasu E, et al. Genome-Wide Analyses of Individual Strongyloides stercoralis (Nematoda: Rhabditoidea) Provide Insights into Population Structure and Reproductive Life Cycles. PLoS Negl Trop Dis. 2016 Dec;10(12):e0005253.

12. Kikuta K, Hayama Y, Watanabe K, Yara T, Shiroma I. A Case of Glucocorticoid-Induced Severe Strongyloidiasis That Occurred in an HTLV-1Carrier With Type 2 Diabetes Mellitus. J Japan Diab Soc. 2016;59(5):369–75.

13. Kinjo T, Nabeya D, Nakamura H, Haranaga S, Hirata T, Nakamoto T, et al. Acute respiratory distress syndrome due to Strongyloides stercoralis infection in a patient with cervical cancer. Intern Med. 2015;54(1):83–7.

14. Kishimoto K, Hirata T, Sotoma A, Fujita J, Tanaka T, Kinjo N, et al. Gastrointestinal endoscopic findings of severe strongyloidiasis. Clinical Parasitology. 2012;23(1):14–6.

15. Kohno Y, Kondo A, Kanazawa Y, Hara K, Izumikawa kinichi, Izumikawa K. A case of strongyloidiasis with hypereosinophilia. Nagasaki Igakkkai zasshi. 2011;86(3):129–33.

16. Kosuga N, Saio Y, Tamaki T, Aoyama H, Hayashi A, Matuzaki A. A case of disseminated strongyloidiasis identified at autopsy of lung cancer. Japanese journal of diagnostic pathology. 2014;31(2):108–13.

17. Kuraoka, Sakiko, Sakuma Takahashi, Kagawa, Ichiro, Junki Toyosawa, Masaya Ishida, Tomo Kagawa, et al. A case of severe strongyloidiasis diagnosed by esophagogastroduodenoscopy and treated successfully with anthelmintic therapy. Gastroenterological Endoscopy. 2018;60(3):237–42.

18. Kurimoto T, Yamauchi A, Tanisaka Y, Sudo K. An autopsy case of respiratory failure due to Strongyloides stercoralis infection. Nihon Naika Gakkai Zasshi. 2014 Apr 10;103(4):969–71.

19. Kurose, Yamagishi T, Kodaka N, Watanabe K, Oshio T, Matsuse. Successful Treatment of Ivermectin in Serologically Diagnosed Pulmonary Strongyloidiasis. Journal of the Japan Society for Respiratory Endoscopy. 2016;38(6):521–5.

20. Kushima A, Takahashi M, Takahashi K, Hachisuka H, Nunomura M. A case of perforation of the intestine caused by Strongyloides Sterocoralis. Journal of Japan Surgical Association. 2010;71(11):2855–9.

21. Mikami S, Maruo M, Yamashita Y, Hirakawa A, Hoshi M, Ide M. A case of strongyloidiasis with a specific colonoscopic picture. Clinical Parasitology. 2016;27(1):9–11.

22. Minematsu H, Hokama A, Makishi T, Arakaki K, Kinjo F, Fujita J. Colonoscopic findings and pathologic characteristics of Strongyloides colitis: a case series. Digestion. 2011;83(3):210–4.

23. Miyazaki M, Tamura M, Kabashima N, Serino R, Shibata T, Miyamoto T, et al. Minimal change nephrotic syndrome in a patient with strongyloidiasis. Clin Exp Nephrol. 2010 Aug;14(4):367–71.

24. Mukaigawara M, Nakayama I, Gibo K. Strongyloidiasis and Culture-Negative Suppurative Meningitis, Japan, 1993-2015. Emerg Infect Dis. 2018 Dec;24(12):2378–80.

25. Mukaigawara M, Narita M, Shiiki S, Takayama Y, Takakura S, Kishaba T. Clinical Characteristics of Disseminated Strongyloidiasis, Japan, 1975-2017. Emerg Infect Dis. 2020 Mar;26(3):401–8.

26. Nabeya D, Haranaga S, Parrott GL, Kinjo T, Nahar S, Tanaka T, et al. Pulmonary strongyloidiasis: assessment between manifestation and radiological findings in 16 severe strongyloidiasis cases. BMC Infect Dis. 2017 May 2;17(1):320.

27. Nagahama M, Inomata H, Nori E, Shimochi H, Samura H, Nishimaki T. A case of disseminated strongyloidiasis after transverse colon lipoma surgery. Journal of Japan Society for Surgical Infection. 2011;8(4):383–6.

28. Nakamura N, Matsushita M, Tahashi Y, Shimatani M, Aoi K, Yanagawa M, et al. Diagnostic yield of double-balloon enteroscopy with intestinal juice analysis for intestinal strongyloidiasis. Dig Endosc. 2010 Oct;22(4):370–2.

29. Nishi Y, Fukushima T, Nomura S, Tomoyose T, Nakachi S, Morichika K, et al. Characterization of patients with aggressive adult T-cell leukemia-lymphoma in Okinawa, Japan: a retrospective analysis of a large cohort. Int J Hematol. 2016 Oct;104(4):468–75.

30. Nishio H, Ochiai T, Tochigi M, Togo K, Tani M, Sunakawa K. Severe drug eruption resulting in death from hemorrhagic pneumonia due to disseminated strongyloidiasis. Hifuka no Rinsho. 2010;52(12):1855–8.

31. Niwa T, Kaneda K, Kobayashi M, Nakajima K, Takamoto M, Kanai S. Disseminated Strongyloidiasis among Steroid Therapy for Dermatomyositis. Nihon Naika Gakkai Zasshi. 2010;99(7):1659–61.

32. Pakdee W, Thaenkham U, Dekumyoy P, Sa-Nguankiat S, Maipanich W, Pubampen S. Genetic differentiation of strongyloides stercoralis from two different climate zones revealed by 18S ribosomal DNA sequence comparison. Southeast Asian J Trop Med Public Health. 2012 Nov;43(6):1333–8.

33. Requena-Méndez A, Buonfrate D, Gomez-Junyent J, Zammarchi L, Bisoffi Z, Muñoz J. Evidence-Based Guidelines for Screening and Management of Strongyloidiasis in Non-Endemic Countries. Am J Trop Med Hyg. 2017 Sep;97(3):645–52.

34. Sasaki T, Yamashita H, Mimori A. A Case of Polymyositis with Severe Gastrointestinal Bleeding Due to Strongyloidiasis. Kansenshogaku Zasshi. 2017;91(3):420–4.

35. Sasaki Y, Taniguchi T, Kinjo M, McGill RL, McGill AT, Tsuha S, et al. Meningitis associated with strongyloidiasis in an area endemic for strongyloidiasis and human T-lymphotropic virus-1: a single-center experience in Japan between 1990 and 2010. Infection. 2013 Dec;41(6):1189–93.

36. Sunagawa K, Nishio H, Kinukawa N, Yamada T, Nemoto N, Ochiai T. An autopsy case of disseminated strongyloidiasis combined with cytomegalovirus infection. Jpn J Infect Dis. 2011;64(2):150–2.

37. Takahashi K, Ogawa M, Ohashi T, Matsuno T, Morioka M. An Immunocompetent Patient with Strongyloides Hyperinfection Syndrome. The Journal of the Japan Society for Respiratory Endoscopy. 2014;36(6):627–31.

38. Takeshita T, InoueKiyohiko, OkazakiNinoi, HirotoshiTeruhisa, HashimotoShigeo, MikiYukio. Pneumobilia, an unusual presentation of strongyloidiasis: a case report. Japanese Journal of Diagnostic Imaging. 2014;34(2):119–24.

39. Tamarozzi F, Martello E, Giorli G, Fittipaldo A, Staffolani S, Montresor A, et al. Morbidity Associated with Chronic Strongyloides stercoralis Infection: A Systematic Review and Meta-Analysis. The American Journal of Tropical Medicine and Hygiene. 2019 Jun 5;100(6):1305–11.

40. Tanaka T, Hirata T, Parrott G, Higashiarakawa M, Kinjo T, Kinjo T, et al. Relationship Among Strongyloides stercoralis Infection, Human T-Cell Lymphotropic Virus Type 1 Infection, and Cancer: A 24-Year Cohort Inpatient Study in Okinawa, Japan. Am J Trop Med Hyg. 2016 Feb;94(2):365–70.

41. Tanaka T, Hirata T, Higashiarakawa M, Kishimoto kazuto, Sotoma A, Kinjo F. A case of Strongyloides hyperinfection syndrome relieved by continuous ivermectin administration. Clinical Parasitology. 2013;24(1):87^90.

42. Tanaka T, Hirata T, Sotoma A, Fujita J, Kishimoto K, Kinjo N. A study of overlapping infections between strongiloides sterocolaris and other intestinal parasites at the University of the Ryukyus Hospital. Clinical Parasitology. 2011;21(1):61–3.

43. Yamanashi H, Kanbara S, Murase K, Maeda T. Eosinophilia, a marker of asymptomatic Strongyloides infection, in a young patient with extrapulmonary tuberculosis. BMJ Case Rep. 2018 Jan 4;2018.

44. Yoshikawa M, Itshiki A, Oji Y, Hirai N, Kitamura T, Misu M, et al. Current status of Strongiroidiasis in Japan based on literature survey, a review of literature reported cases from June 2013 to February 2020. Clinical Parasitology. 2020;31(1):28–33.

45. Zuikeran Y, Miyagi M, Chinen H, kamichi H, Nagayama K, Saito M. A case of strongyloideasis detected by cytology of aspirated sputum. The Journal of the Japanese Society of Clinical Cytology, Kyushu Branch. 2010;41:67–71.

**Teniasis and cysticercosis (11)**

1. Braae UC, Hung NM, Satrija F, Khieu V, Zhou XN, Willingham AL. Porcine cysticercosis (Taenia solium and Taenia asiatica): mapping occurrence and areas potentially at risk in East and Southeast Asia. Parasit Vectors. 2018 Nov 29;11(1):613.

2. Del Brutto OH. Neurocysticercosis among international travelers to disease-endemic areas. J Travel Med. 2012 Apr;19(2):112–7.

3. Hara W, Ohji S, Mitsui T, Fukahori H, Nomura K. A nepalese case in Japan: neurocysticercosis with a solitary nodular brain lesion that had difficulty distinguishing it from cerebral tuberculoma (Japanese). Vol. 30, Shinkeichiryogaku. 2013. p. 45–50.

4. Kawajiri S, Yamauchi T, Kitai R, Arai T, Higashino Y, Tsunetoshi K, et al. A case of cysticercosis showing peculiar MRI image changes (Japanese). Vol. 41, CIkenkyu. 2019. p. 37–40.

5. Maeda T, Ito A, Sako Y, Yamasaki H, Oyaizu N, Odawara T, et al. Neurocysticercosis case with tuberculoma-like epithelioid granuloma strongly suspected by serology and confirmed by mitochondrial DNA. BMJ Case Rep. 2011 Jul 15;2011.

6. Sako Y, Takayanagui OM, Odashima NS, Ito A. Comparative Study of Paired Serum and Cerebrospinal Fluid Samples from Neurocysticercosis Patients for the Detection of Specific Antibody to Taenia solium Immunodiagnostic Antigen. Vol. 43, Tropical Medicine and Health. 2015. p. 171–6.

7. Sato A, Nakamura I, Fujita H, Fukushima S, Mizuno Y, Fujii T, et al. Neurocysticercosis with Diplopia Responds Well to Albendazole. Vol. 55, Internal Medicine. 2016. p. 1219–22.

8. Shoji H, Hirai T, Ishikura T, Takuma T, Okino T, Wakatsuki Y, et al. A case of cysticercosis with multiple lesions in the brain and femoral muscles (Japanese). Vol. 87, Kansenshogaku Zasshi. The Journal of the Japanese Association for Infectious Diseases. 2013. p. 608–12.

9. Yamasaki H, Sugiyama H, Morishima Y, Ohmae H, Shiinogi S, Okuyama K, et al. A case of racemose neurocisticercosis Racemose (Japanese). Vol. 21, Clinical Parasitology. 2011. p. 29–32.

10. Yanagida T, Sako Y, Nakao M, Nakaya K, Ito A. Taeniasis and cysticercosis due to Taenia solium in Japan. Parasit Vectors. 2012 Jan 17;5:18.

11. Yokota K, Furukawa K. A Case of Cysticercosis with Multiple Intracerebral and Intramuscular Nodular Lesions (Japanese). Vol. 86, Kansenshogaku Zasshi. The Journal of the Japanese Association for Infectious Diseases. 2012. p. 27–30.

**Trachoma (0)**

**Yaws (0)**
