# Supplementary material for: The current status of neglected tropical diseases in Japan: A scoping review
Source: PLoS Negl Trop Dis. 2024 Jan 2;18(1):e0011854. doi: 10.1371/journal.pntd.0011854 (PMC10786391; doi:10.1371/journal.pntd.0011854)
Supplement: S3 Document — (DOCX) [file pntd.0011854.s003.docx]

Supplementary Document 3: Map of Japan showing geographical locations of prefectures and regions that are mentioned in this review.


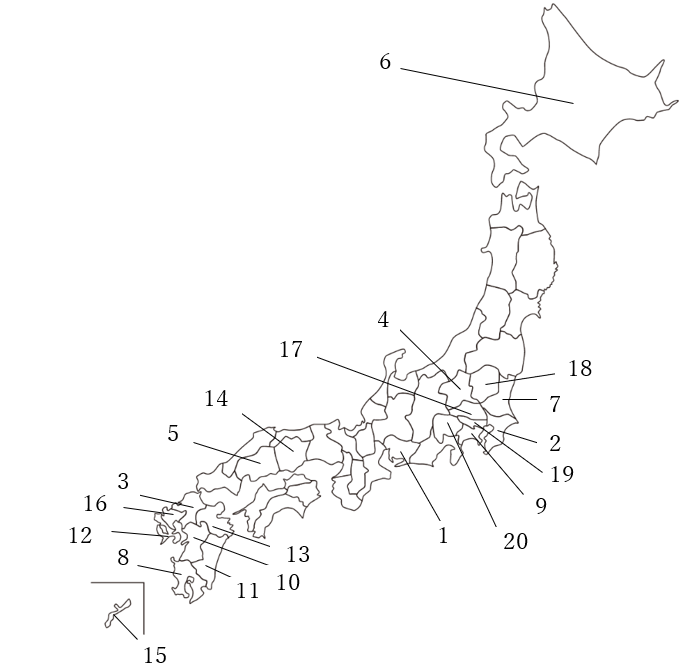


| 1. Aichi Prefecture 2. Chiba Prefecture 3. Fukuoka Prefecture 4. Gunma Prefecture 5. Hiroshima Prefecture 6. Hokkaido Prefecture 7. Ibaraki Prefecture 8. Kagoshima Prefecture 9. Kanagawa Prefecture 10. Kumamoto Prefecture | 1. Miyazaki Prefecture 2. Nagasaki Prefecture 3. Oita Prefecture 4. Okayama Prefecture 5. Okinawa Prefecture 6. Saga Prefecture 7. Saitama Prefecture 8. Tochigi Prefecture 9. Tokyo 10. Yamanashi Prefecture |
| --- | --- |
| 1. Kanto Region (2. Chiba Prefecture, 4. Gunma Prefecture, 7. Ibaraki Prefecture, 9. Kanagawa Prefecture, 17. Saitama Prefecture, 18. Tochigi Prefecture, and 19. Tokyo) 2. Kyushu Region (3. Fukuoka Prefecture, 8. Kagoshima Prefecture, 10. Kumamoto Prefecture, 11. Miyazaki Prefecture, 12. Nagasaki Prefecture, 13. Oita Prefecture, and 16. Saga Prefecture) | |

A copyright-free map was provided by the website Hakuchizu nurinuri (<https://n.freemap.jp/>). Direct link to the base layer of the map: <https://n.freemap.jp/tp/Japan>. Link to the terms of use: <https://www.freemap.jp/about_use_map.html>.
